# Human learning of probability distributions is biased toward moderate structural complexity

**DOI:** 10.1038/s41467-026-76247-3

**Published:** 2026-08-08

**Authors:** Tianyuan Teng, Li Kevin Wenliang, Hang Zhang

**Affiliations:** 1 Center for Life Sciences, Peking University, Beijing, China; 2 Max Planck Institute for Biological Cybernetics, Tuebingen, Germany; 3 Gatsby Computational Neuroscience Unit, University College London, London, UK; 4 School of Psychological and Cognitive Sciences, Peking University, Beijing, China; 5 PKU-IDG/McGovern Institute for Brain Research, Peking University, Beijing, China; 6 Key Laboratory of Machine Perception (Ministry of Education), Peking University, Beijing, China; 7 Beijing Key Laboratory of Brain-Computer Interface and Mental Health Modulation, Peking University, Beijing, China; 8 Present Address: Google DeepMind, London, UK

**Keywords:** Human behaviour, Learning and memory

## Abstract

Inferring hidden environmental structures, which commonly involves learning arbitrary probability distributions from limited samples, is essential to optimal and adaptive behaviors across various cognitive domains. However, it remains largely unknown how the internal representations constructed by humans may deviate from actual probabilistic structures, and what computational processes, operated under inherent cognitive limitations, give rise to these representations. We first develop a structured distribution report task to reveal human participants’ internal representations, with findings verified in a further distribution recognition task. Across eight behavioral experiments (including one pre-registered study) in two modalities, participants estimate the overall probability density reasonably well, but exhibit a systematic bias toward moderate structural complexity, reporting too many clusters when the true distribution is a single Gaussian and too few when it contains many clusters. We then build a series of learning models in the framework of approximate Bayesian inference fit to our behavioral data. Through model comparisons, we reconstruct the prior beliefs guiding the evolution of participants’ internal representations. The best-fitting model for human reports reduces structure growth rate as complexity increases, effectively constraining the complexity of internal representations within memory limitations.

## Abstract (web summary)

Humans build internal models from online observations to adapt to new environments. Here, the authors show that individuals are biased towards building models with moderate structural complexity, regardless of true complexity.

## Introduction

Probabilistic models learned from experience allow the brain to handle uncertainty efficiently, interpreting ambiguous sensory signals^1,2^, making quick predictions about the uncertain environment^3^, and enabling flexible decision making^4–6^.

There are two quintessential aspects of learning probabilistic models to represent uncertainty: the probability distribution function of specific stimulus features and the structures of other variables that are not observed but directly influence the feature probability. While the former specifies how frequently the observable features occur, the latter describes a series of plausible mechanisms or rules that generate the features, internalized and likely simplified by the brain to understand the world.

For example, legal documents tend to have higher frequencies of long words and sentences than novels, reflecting the underlying writing style and content topics. As another example, adjectives in different languages may precede or follow the modified nouns, reflecting the latent structure imposed by different grammatical rules. To highlight the duality of uncertainty representation, we will henceforth refer to internal probabilistic models in the brain as structured distributions, or structured densities.

How does the brain learn structured densities^7^? From the learner’s perspective, the difficulty lies in the potential vastness of the hypothesis space compared to the finiteness of the available data, since the limited observed samples can be explained by many alternative structures equally well. It makes sense for the brain to approach this inductive problem with its prior knowledge of how data are produced from structured densities, subject to biases arising from inevitable cognitive constraints (prior beliefs and inductive biases are analogous concepts from different fields^8^). The importance of inductive bias in learning probabilistic models is exemplified in modern artificial intelligence methods, such as latent variable models built from artificial neural networks, where choices of model architecture and training objectives lead to different compressed probabilistic representations of the data and hidden structures^9,10^. In human cognition, the seminal work of John Anderson on category learning^11^ transforms the inductive problem into Bayesian inference based on a non-parametric prior that allows incremental and potentially infinite expansion of structures. His algorithm is further generalized to explain a variety of behavioral phenomena^12–18^. These models of structure learning in humans, although statistically elegant^12^, can exceed human cognitive limitations (e.g., memory capacity required for storing potentially infinite structures), even with efficient sampling algorithms (see ref. ^19^, for theoretical properties of such a non-parametric prior).

From the experimenter’s perspective, measuring and modeling the virtually infinite-dimensional densities, while inferring the associated hidden structures, is doubly challenging. Most previous studies rely on indirect measures, such as behavioral patterns that would arise given a learned structured density, including biases in the reconstruction/classification of a noisy observation^14,20–23^, speed of adaptation in learning^5,16,24,25^ and motor^18^ tasks, and distribution-based gambling biases^26,27^. These measures essentially project high-dimensional densities into low-dimensional (often uni-dimensional) decision spaces, with the projection constrained by different task-dependent loss functions. Moreover, different combinations of uncertain beliefs and loss functions can trade off with each other and produce similar behavioral predictions^20,28^. Thus, identifying the representation of high-dimensional uncertainty based on participants’ low-dimensional reports is intrinsically difficult. These measurement limitations leave the literature inconclusive about humans’ inductive biases in representing distributional structures. Some studies favor simpler, unimodal representations^22,29,30^, others find both unimodal and multimodal structures^31–34^, while still others support more complex, multimodal representations^26,35^.

In this study, we developed a novel task to reveal more detailed and previously overlooked patterns in the structured densities people learn from samples. In each trial, participants observed sequential samples and reported the parameters of the hidden clusters that best describe the densities learned. This encourages participants to report finer structures of the acquired density that would be lost had they only reported its coarse statistics of the mean and variance. Surprisingly, participants consistently reported 2–4 barely overlapping clusters, even when the true distribution was a single Gaussian, and this bias became more prominent with increased sample size. Across three experiments, we found such structure illusion to be invariant across different scales, across visual and numeric modalities.

To model how this illusory structure perception could arise from an online, sample-by-sample learning process, we constructed a set of computational models, including Bayesian models with non-parametric priors, to explore possible inductive biases of humans in processing probabilities under limited cognitive resources. Despite the challenges posed by the indefinite dimensionality of the structured report data and the inherent stochasticity of the learning process, we successfully estimated all parameters for each model to fit each participant’s data, using novel modeling and computational techniques. Our best-fitting model featured several deviations from previously conjectured algorithms of the mind, such as a slower increase in the number of clusters when more clusters existed, which conforms to memory constraints. Five additional forced-choice experiments, including one pre-registered experiment, validated our model-agnostic and modeling findings.

## Results

**Fig. 1:**
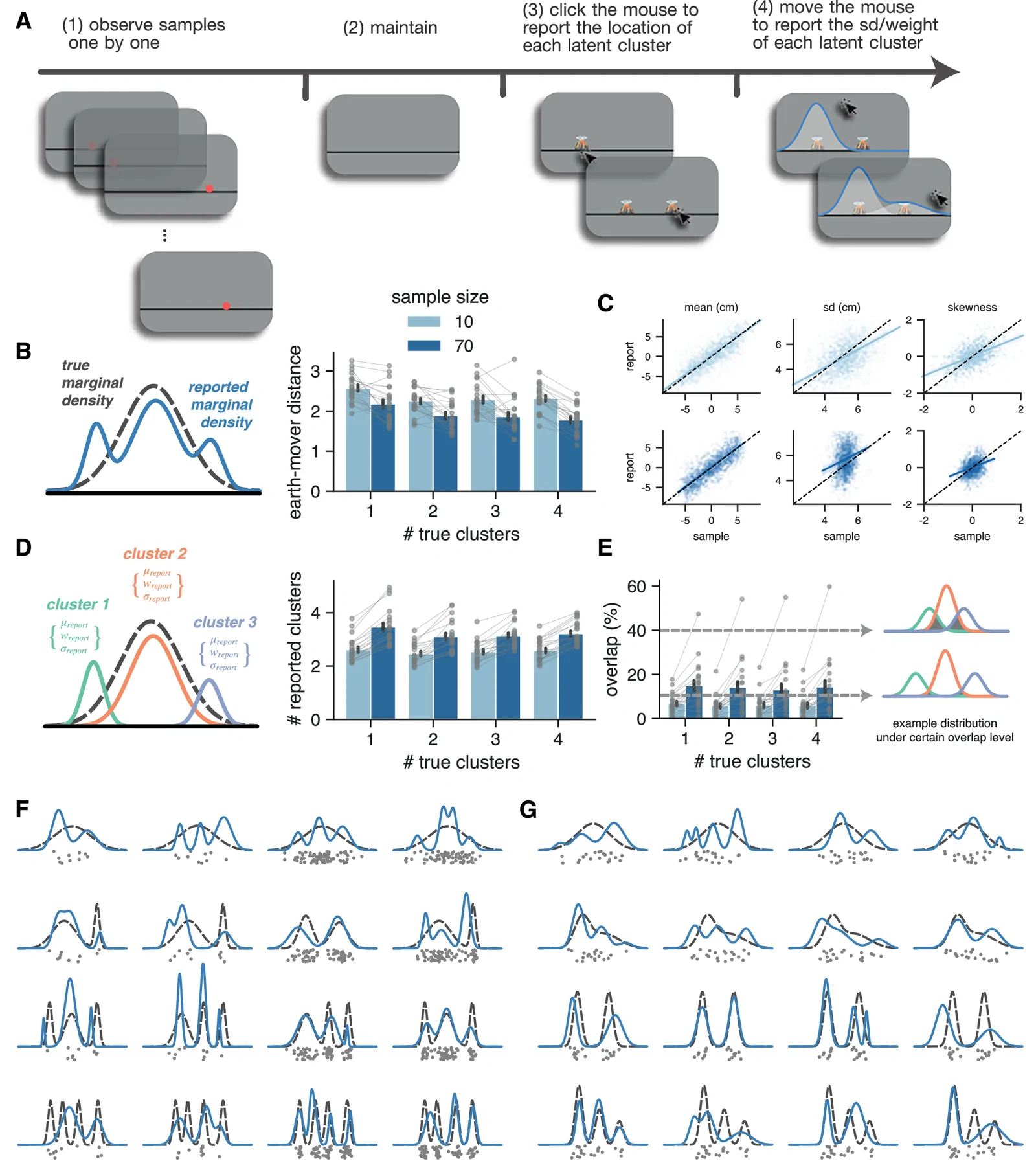
The structured density estimation task and behavioral results. **A** Trial procedure. Participants observed a set of samples ("magic lava stones'') drawn from a true generative distribution. They then estimated the number of latent clusters ("invisible volcanoes'') and reported the properties of each cluster, essentially performing a probability density estimation by inferring the underlying structure. The cumulative effect of these latent causes was visualized as a blue line, which was updated in real-time based on the participant’s input. **B–F** Results of Experiment 1. **B** The earth-mover distance between the true and the reported densities decreased with increasing sample size, indicating continual learning. **C** The mean, standard deviation, and skewness of the reported density were correlated with those of the presented samples. Top and bottom rows are respectively for sample size 10 and 70. **D** The number of reported latent clusters under different conditions. Although the true number of clusters varied (1 to 4), participants typically reported 2–3 clusters, failing to accurately learn the true structure. With more samples, participants increased the number of reported clusters rather than converging to the true structure. **E** Low overlap between adjacent clusters caused the reported marginal density to appear bumpy. **F** Examples of participants' reported structured densities (blue solid) in response to observations (gray dots) drawn from hidden underlying distributions (gray dashed) in individual trials. To enhance visualization, each panel is centered at the mean of the true generative distribution. **G** Examples of participants' reports in Experiment 2. Error bars indicate SEM across participants (*n* = 21, 20 in experiments 1 and 2, respectively).

To investigate how humans learn structured densities, we designed a behavioral task (Fig. 1A) to let participants freely and naturally report the high-dimensional parameters of their internal distribution. In each trial, participants observed samples drawn from a true distribution, described as “observing magic lava stones emitted from invisible volcanoes”. The true distribution could be a single Gaussian or a mixture of Gaussians with a varying number of clusters. Participants predicted the distribution of future samples by detailing the underlying causes, i.e., the number of volcanoes, their locations, widths, and relative emission frequencies. That is, participants were effectively asked to identify the means, standard deviations, and weights of a Gaussian mixture. No feedback was provided so that we could measure innate preferences in human density estimation.

### Illusory perception of latent structures

In the first experiment (*n* = 21), the true generative distribution (see dashed lines in Fig. 1F for examples and Fig. S2A for the whole set) varied across trials in the number of clusters (i.e., number of Gaussian components, from 1 to 4) as well as in its center (i.e., mean). On each trial, participants observed either 10 or 70 samples, thus providing their estimates under two strengths of distributional evidence. By varying the sample size, we avoided interrupting each trial to query for a complex distributional report and also allowed us to observe convergence patterns, if any.

Before analyzing the reported structures, we examined the reported density as a sanity check. Participants’ density reports followed the variation of the true density, whose estimation error, as quantified by the earth mover distance between the true and the reported densities, was lower than a permutation baseline constructed from randomly shuffled pairs by 47.8% ± 5.3% (permutation test for each participant, *p*s < 0.001,  ± denotes SD across participants). Moreover, this error decreased with the number of observed samples (Fig. 1B, repeated-measures ANOVA, effect of sample size, *F*(1,20) = 83.21, *p*  < 0.001, ${\eta }_{\,{\mbox{G}}\,}^{2}$ = 0.25; effect of true cluster number, *F*(3,60) = 15.12, *p*  < 0.001, ${\eta }_{\,{\mbox{G}}\,}^{2}$ = 0.12; negligible interaction, *F*(3, 60) = 0.89, *p* = 0.449, ${\eta }_{\,{\mbox{G}}\,}^{2}$ = 0.01), indicating continuing learning from samples. Besides, regression analyses of the moments, i.e., mean, standard deviation (sd), and skewness (skew) of the reported density against those of samples show significantly greater-than-0 slopes (Fig. 1C, *p*s  < 0.001, see Methods for details of testing regression), providing further evidence for successful learning of the true density.

These regression analyses also suggest systematic deviations of the reported density from the true density. In particular, the regression slope was further from 1 for higher-order moments (*β*_mean_ > *β*_sd_, *t*(20) = 6.29, *p*  < 0.001, 95%CI = [0.18, 0.42], *d* = 1.53; *β*_sd_ > *β*_skew_, *t*(20) = 3.21, *p* = 0.012, 95%CI = [0.03, 0.25], *d* = 0.71), suggesting that participants are less sensitive to higher-order moments. The deviations from ground truth were more evident in the raw density reports (Fig. 1F), showing as multimodal bumps, even when the true distribution was as smooth as Gaussian.

Further analysis on the reported structures reveals that this bumpiness partly resulted from participants’ illusory perception of latent structures—they tended to report 2–3 clusters (79.0% ± 18.0% among all trials), and rarely reported 1 cluster (2.0% ± 2.5% among all trials). As participants observed more samples, instead of closing in on the true latent structure, they reported more clusters (Fig. 1D, *F*(1, 20) = 124.31, *p*  < 0.001, ${\eta }_{\,{\mbox{G}}\,}^{2}$ = 0.35). The effects of the true cluster number (*F*(3, 60) = 12.05, *p*  < 0.001, ${\eta }_{\,{\mbox{G}}\,}^{2}$ = 0.04) and its interaction with sample size (*F*(3, 60) = 6.23, *p*  < 0.001, ${\eta }_{\,{\mbox{G}}\,}^{2}$=0.01) were also significant, but their directions were surprising: for 70 samples, participants reported more clusters in the 1-cluster condition than the 2, 3, and 4-cluster conditions (post-hoc tests, 1- vs. 2-cluster condition, *t*(20) = 5.39, *p*  < 0.001, 95%CI = [0.17, 0.54], *d* = 0.74; 1- vs. 3-cluster condition, *t*(20) = 4.12, *p* = 0.003, 95%CI = [0.10, 0.54], *d* = 0.67; 1- vs. 4-cluster condition, *t*(20) = 3.11, *p* = 0.025, 95%CI = [0.02, 0.47], *d* = 0.51). The reported number of clusters was on average 3.03  ± 0.55 when the true distribution was a single Gaussian (Fig. 1D, see Fig. S2B for the frequency of reported cluster numbers). Notably, the reported clusters were almost non-overlapping (Fig. 1E). The tiling of the non-overlapping clusters is associated with the bumpiness of the marginal density.

In Experiment 2 (*n* = 20), we tested the generality of these findings using a representative set of generative distributions from previous studies (Fig. S3A)^14,17,31,32,34,36^, whose number of local maxima (i.e., bumps) ranged from 1 to 3. The major results of Experiment 1 were replicated: participants preferred to report the distribution as a mixture of 2–3 non-overlapping clusters (Fig. 1G, Fig. S3BC), even when the true distribution was a Gaussian distribution.

In Experiment 2, each sample sequence (jittered as a whole) was presented twice to test the consistency of human density learning^37,38^. The reported cluster number of a specific trial was closer to its “twin” trial than to that of a different sequence sampled from the same (*t*(19) = 2.25, *p* = 0.036, 95%CI = [0.00, 0.05], *d* = 0.50) or a different (*t*(19) = 5.19, *p*  < 0.001, 95%CI = [0.05, 0.12], *d* = 1.16) generative distribution (Fig. S3D). Meanwhile, participants’ structure learning was noisy: for the same sample sequence, they reported the same number of clusters in only 54.1%  ± 10.3% of the trials.

### Modality generality and scale invariance

**Fig. 2:**
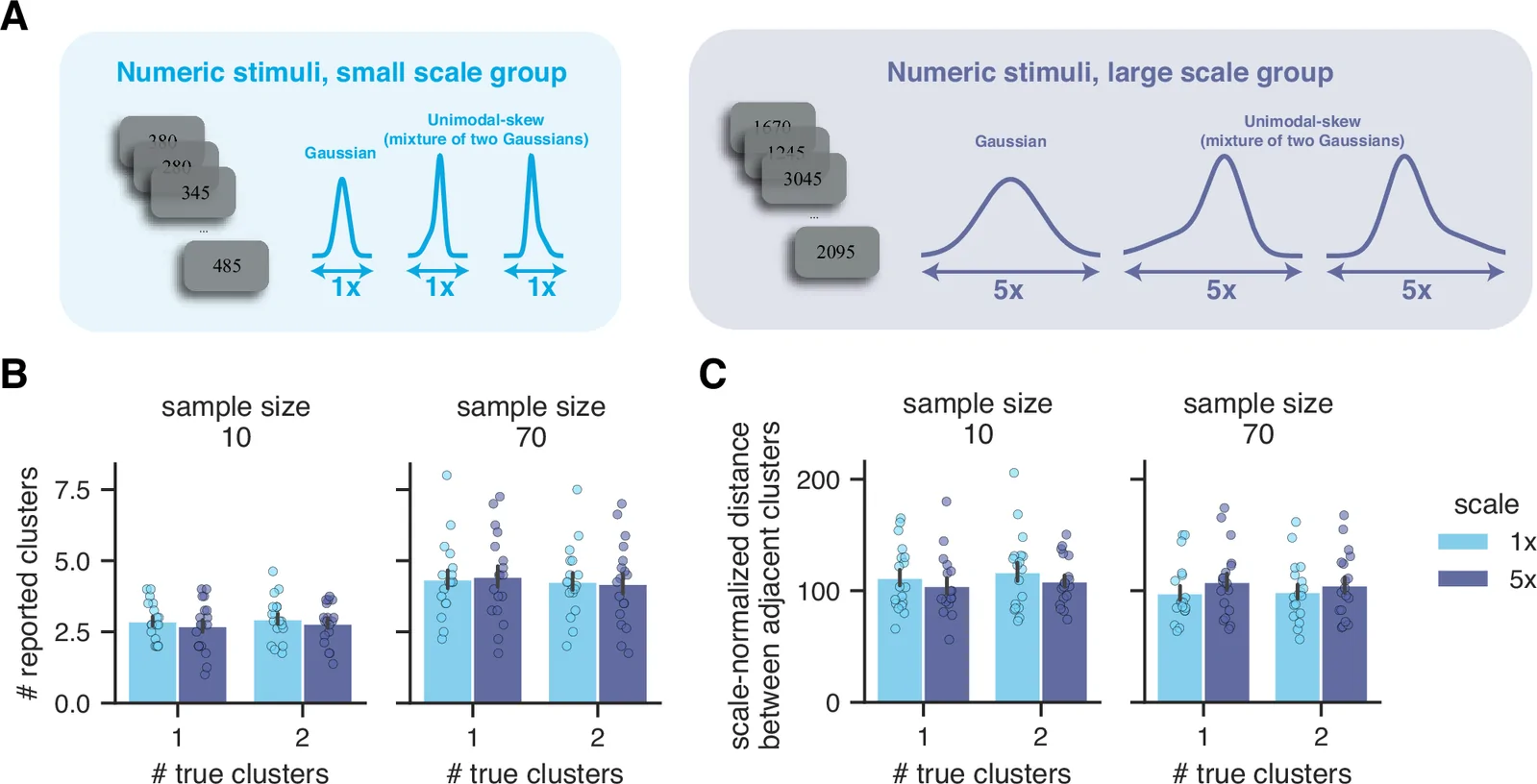
Learning a numeric distribution. **A** Two groups of participants learned numeric distributions with different scales (sd 100 vs. 500, denoted by 1x vs. 5x). The reporting procedure was similar to that of Experiments 1 and 2, except that participants did not report the widths of the latent clusters. The true generative distribution was either a Gaussian distribution or a mixture of two Gaussian clusters. **B** Participants in both scale groups reported a similar number of clusters. **C**: The distance between adjacent reported clusters increased linearly with the scale of the distribution. Error bars indicate SEM across participants, *n* = 18 per group.

Humans can handle uncertainty over quantities from different modalities at different scales. To verify that our findings above are not specific to the visual modality and the particularly tested scale, we performed Experiment 3, using sequences of Arabic numbers as stimuli (illustrated in Fig. 2A). Scale was manipulated between groups: the large-scale group observed distributions five times wider than the small-scale group. As in Experiments 1 and 2, participants reported more complex distributional structures for ground truth with simple structures (i.e., reported more clusters than the true number of clusters, *p*  < 0.001 for all conditions), and this effect was stronger when observing more samples (Fig. 2B, *F*(1, 34) = 120.12, *p*  < 0.001, ${\eta }_{\,{\mbox{G}}\,}^{2}$ = 0.31). Unlike previous experiments, Experiment 3 exclusively used unimodal distributions (Gaussian or skewed), eliminating the possibility that participants’ over-complex structure reports merely reflected environmental priors. The distribution scale hardly affected latent structure learning, with negligible group differences in reported cluster numbers (Fig. 2B, *F*(1, 34) = 0.05, *p* = 0.831, ${\eta }_{\,{\mbox{G}}\,}^{2}$ = 0.00) or in scale-normalized distances between adjacent clusters (Fig. 2C, *F*(1, 33) = 0.06, *p* = 0.808, ${\eta }_{\,{\mbox{G}}\,}^{2}$ = 0.00, one participant was excluded from this ANOVA due to missing reports, see Methods for details).

These findings suggest that participants’ representation of latent structures was adaptive and invariant to the scale of the true distribution. Participants reported more clusters in the numeric modality compared to the visual-spatial modality (*F*(1, 55) = 4.96, *p* = 0.030, ${\eta }_{\,{\mbox{G}}\,}^{2}$ = 0.07, only the matched condition of Experiment 1 included in the test), echoing differences in working memory capacity across feature modalities.

To summarize, in both visual and numeric modalities, we found systematic over-complication in the structured distributions learned by humans when the true distribution was simple. Rather than converging to the ground truth, participants reported more clusters when increasing the observations from 10 to 70 samples. However, participants did not produce arbitrarily complex structures, reporting fewer clusters when the true distribution exceeded three clusters. The reported clusters were well-separated, resulting in distribution densities that are bumpier than the true generative distributions. Given the complexity of the task and these behavioral patterns, it is difficult to infer the inductive biases directly from summary statistics. We therefore built computational models to simulate such behavior and identify key parameters that underlie online density estimation by humans.

### Modeling structure learning as a bounded-rational expansion process

Given the sequential nature of observations in our task and also real life, a reasonable learner should adjust properties of the existing clusters (e.g., locations and widths) when the current structure covers the new observation, or else expand the structure with a new cluster when new observations are sufficiently surprising under the current structure. In fact, one type of observation-driven expansion process was mathematically proven to yield over-clustering when the true structure is a single Gaussian^19^, closely aligning with our key empirical finding—humans’ illusory structure perception. This conceptual alignment forms the theoretical foundation of our model.

We designed the observation-driven expansion process as Bayesian inference with two structure-controlling inductive biases: one specifies the tendency of structure expansion and cluster assignment for new observations, extended from the Chinese restaurant process (CRP); and the other controls the prior over cluster center and width before seeing any observation. We give an overview of the model here, and leave precise mathematical details in Methods and Supplementary Materials.

First, structure expansion must be economical to conserve humans’ limited memory, which is realized by a decay rate parameter *r*  ≥ 0 that slows down model expansion as the internal model becomes large. Second, cluster weights may be distorted following Stevens’ power law^39–41^ controlled by the distortion rate parameter *β*, a form of distortion similar to that applied to probabilities in decision under risk (e.g., ref. ^42^).

**Fig. 3:**
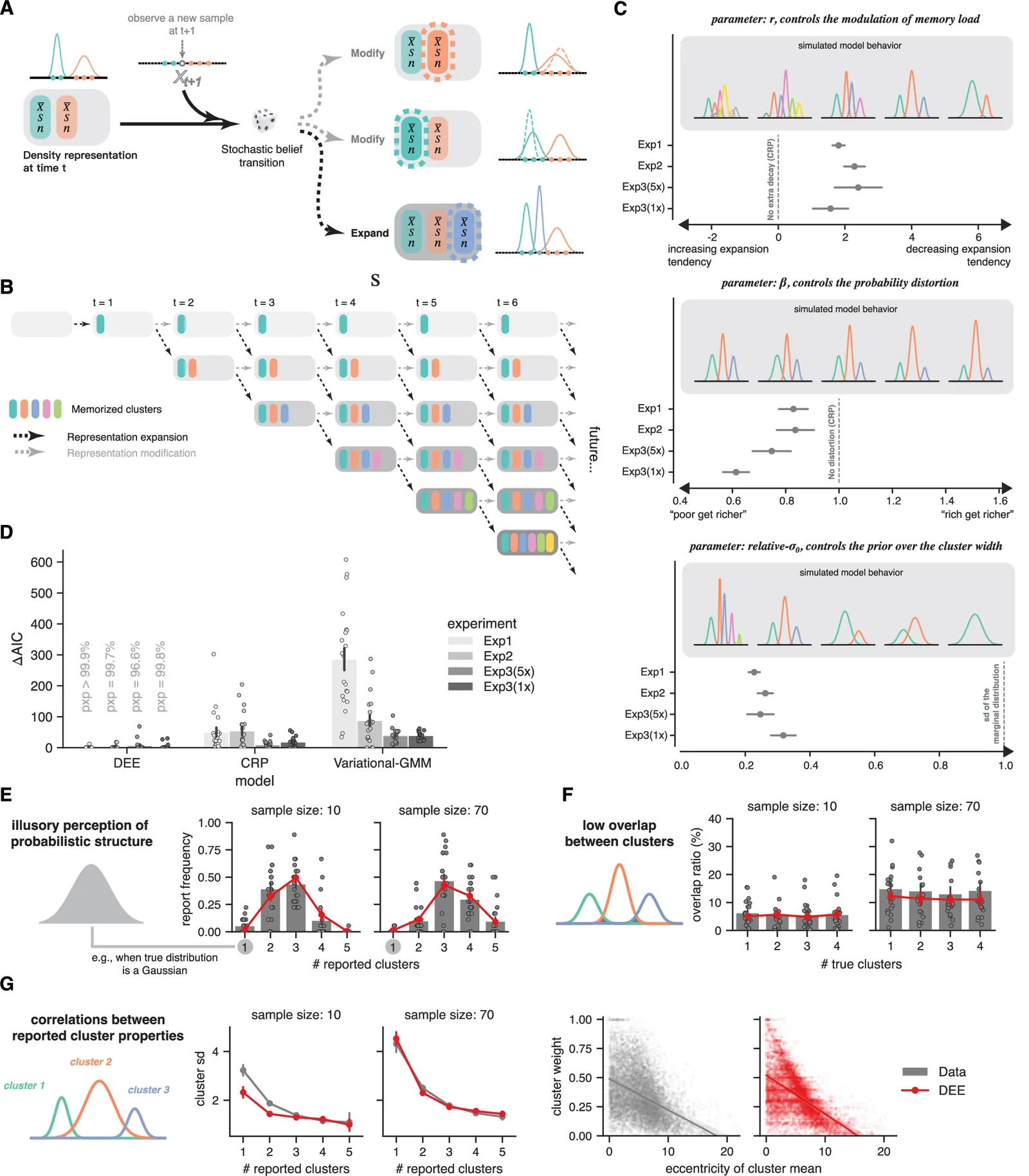
Modeling human distribution learning as a Distorted Economical Expansion (DEE) process. **A** The model assumes that after observing a new sample, the learner either (1) updates the properties of an existing cluster by assigning the observation to that cluster, or (2) expands their represented probabilistic structure by creating a new cluster. **B** Belief transition map. The model assumes that as participants observe more samples, their beliefs about the structure transition through one path of the expansion tree. The grayscale of the rounded rectangle (memory space) indicates the working memory load required to represent such beliefs. By assuming that expansion probability is modulated by working memory load, DEE avoids memory-taxing dark-gray area. **C** Three fitted model parameters that characterize the DEE process: the expansion rate exponent *r*, distortion rate *β*, and the relative prior cluster width *σ*_0_ (see equations (1) and (2)) Each panel’s upper part illustrates the simulated DEE representation after observing 50 samples from a Gaussian distribution under different parameters, while the lower part shows the fitted parameters. **D** DEE outperformed both the Chinese Restaurant Process (CRP) and the non-sequential-learning variational Gaussian mixture model (variational-GMM). ΔAIC represents each model’s AIC relative to the best-fitting model for each participant, then averaged over participants. Protected exceedance probabilities (pxp) for each model were computed by group-level Bayesian model selection and are shown above the bars. We also fitted 11 CRP-based alternative models using different cognitive constraints, but none surpassed DEE; see Fig. S6 for full model comparison results. **E–G** Simulated behavior of the fitted DEE models in Experiment 1. The model successfully captured the inconsistency in learning probabilistic structures (**E**), the low overlap between adjacent clusters (**F**), and the correlations between reported cluster properties (**G**, the eccentricity is calculated as the distance between the reported cluster center and the mean of the presented samples). See Figs. S2, S3, S4 for comprehensive simulation results across all three experiments. Error bars indicate SEM across participants (*n* = 21, 20, 18, 18 in experiments 1, 2, 3 (5x), and 3 (1x), respectively).

The effects of these two parameters are shown in Fig. 3C and Fig. S10, together with the effect of the prior cluster width *σ*_0_. We thus refer to this process that generates structured density as the Distorted Economical Expansion (DEE) process.

DEE builds a structured density step-by-step from sequentially presented observations. We assume that participants keep a single possible cluster assignment for each past observation. Each new observation could trigger one of two internal events (Fig. 3A): structure modification or structure expansion. The probability of each event follows the posterior over cluster assignment given the incoming observation, essentially implementing a single-particle approximation of Bayesian clustering^12,43^. We compared DEE with a range of alternative models, including the following two representative baselines: a non-sequential-learning Bayesian agent (named the variational-GMM) that memorizes all observations before clustering them; and CRP, a widely-used prior for modeling human and animal learning in structured environments^12,43–45^; it can be seen as a special case of DEE with no economical expansion (*r* = 0) or probability distortion (*β* = 1).

To account for additional errors during the reporting stage after density learning, we introduced a structured noise process termed the aleatoric component, which is similar to the common Gaussian residual noise (or lapse rate) in modeling fixed-dimensional report quantities, but is far more sophisticated to cover the additional constraints on reported cluster properties (i.e., domain of GMM parameters) and the flexibility seen in our participants. Further, the single-particle inference emulates the stochasticity in cluster assignment by the participants (Fig. 3B).

Taken together, our overall framework is a modular, flexible, and cognitively plausible forward process that produces structured densities given sequential observations, allowing us to test diverse hypotheses about how participants perform the task; this enables the discovery and validation of deeper cognitive insights from more complicated forms of report.

Evaluating the likelihood on participants’ reported cluster properties requires averaging over a large number of simulations (runs of the forward process) to marginalize out variables unobservable by the experimenter. Similar computational challenges had often constrained previous modeling work to fit only a subset of the free parameters. We overcame this challenge by using graphics processing units (GPUs) to perform fast and parallel simulations and reliable likelihood estimates, allowing us to fit *all* model parameters by maximum likelihood. Our parameter recovery analysis showed that the parameters could be reliably recovered (Fig. S7), supporting the robustness and identifiability of our modeling approach.

The DEE model accurately reproduces patterns of human density estimation across multiple dimensions, not only in the metrics for which the loss function was optimized (e.g., reported cluster number, Fig. 3E) but also in the moments of the structured densities (mean, standard deviation, and skewness, Fig. S2C), the overlap between clusters (Fig. 3F), and the covariation of pairs of features (Fig. 3G). Examples of these behavioral dimensions in Experiment 1 are shown in Fig. 3E–G, with full simulations presented in Figs. S2, S3,S4. Further, across all three experiments, the fitted values of the important parameters *r* and *β* align with our theoretical expectations: *r* is greater than zero, indicating a reduced tendency toward creating new clusters, while *β* is less than one, suggesting that cluster weights are distorted to be more similar.

The prior cluster width *σ*_0_ also significantly influences the learning of latent structures. For example, when learners strongly believe that cluster widths are narrow, they tend to learn more clusters (Fig. 3C). After normalizing *σ*_0_ relative to the overall distributional scale (termed *relative*-*σ*_0_), the fitted parameter showed high consistency across modalities and experiments, with values roughly matching one-third of the standard deviation of the marginal density. This finding aligns with statisticians’ data-dependent heuristics in Bayesian clustering^46^, which are often justified as “natural on the basis of symmetry and scale invariance”^47^.

Model comparisons reveal that the DEE model outperforms notable alternatives—CRP and non-sequential variational-GMM (Fig. 3D; see also Fig. S11 for the predicted behavior of alternative models). We also attempted to introduce alternative cognitive constraints to CRP, giving a total of 11 CRP-based variants (including CRP), none of which outperformed DEE (Fig. S6). This further supports DEE as the best model.

To summarize, a Bayesian learner with a DEE prior best describes how humans construct a structured representation from random samples. The tendency to increase the representation complexity along with each new observation causes the learner to fail in learning a simple structure as a Gaussian. Moreover, model fitting suggests that such an increasing tendency is modulated by limited memory resources, reflecting the balance between representation complexity and cognitive cost.

### Validation in distribution recognition tasks

**Fig. 4:**
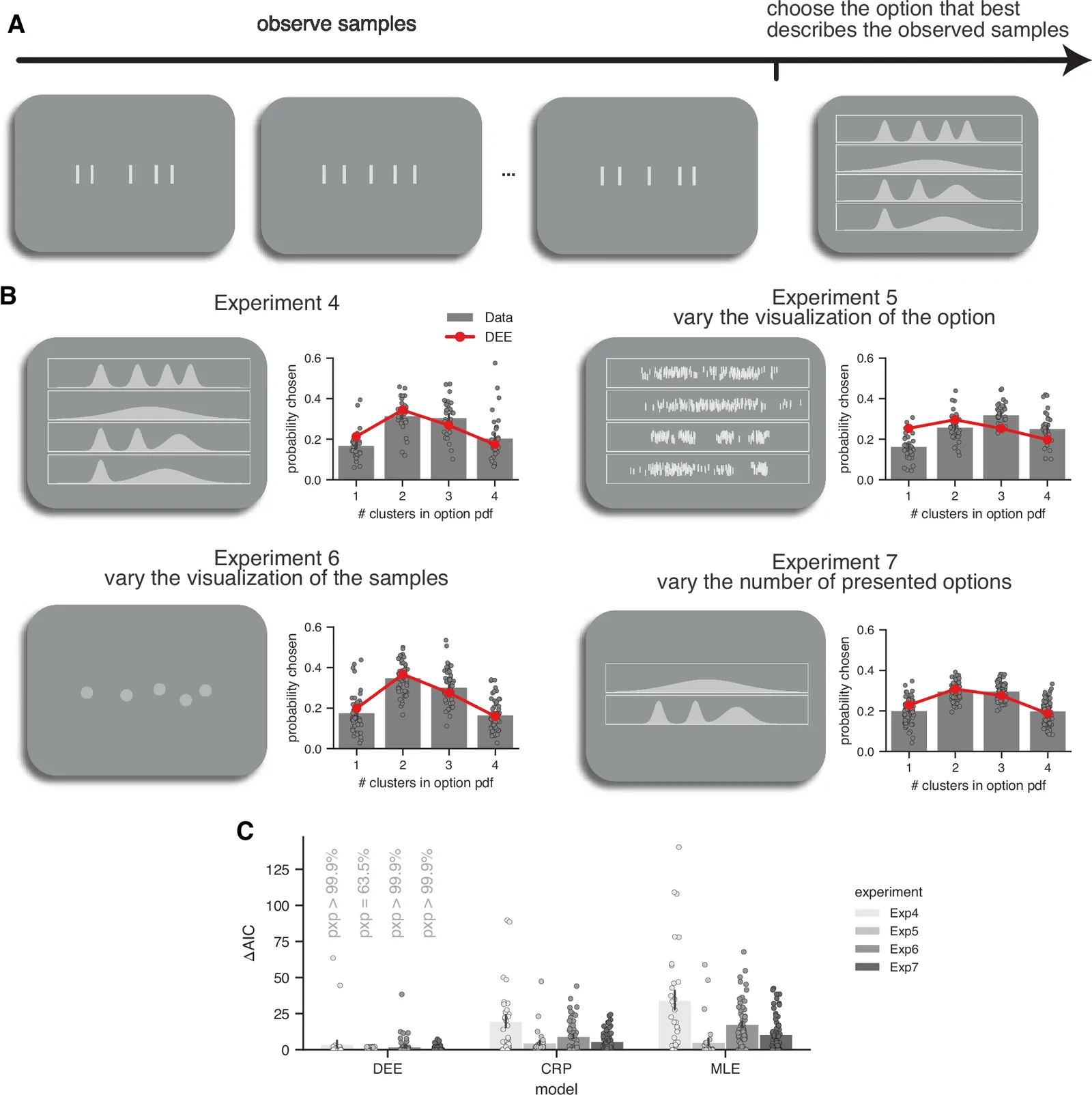
The probability density recognition task. **A** Trial procedure. Participants observed a set of samples drawn from a true generative distribution and then chose which of the presented options best described the pattern of the samples. The true generative distribution varied by trial, with the true number of clusters equally likely to be 1, 2, 3, or 4. The presented options had the same overall mean and variance, preventing participants from relying on these obvious density properties. **B** A recognition bias toward options with 2 or 3 clusters was consistently observed across four experiments. Experiment 4 served as the baseline for Experiments 5-7, which varied in visualization and presentation format: Experiment 5 used samples instead of a density curve, Experiment 6 presented samples as dots instead of vertical bars, and Experiment 7 reduced the number of options from 4 to 2. **C** Model comparisons show that participants' recognition bias was better explained by Distorted Economical Expansion (DEE) compared to Chinese Restaurant Process (CRP) and the Maximum Likelihood Estimation (MLE) models. Error bars indicate SEM across participants (*n* = 31, 33, 55, 67 in experiments 4–7, respectively).

In the structured density estimation task, participants were required to report the latent structure of the distribution. To exclude the possibility that our findings of illusory structures and economical expansion process are artifacts of the explicit requirement of structural learning, we performed Experiments 4–8, where participants saw similar sample sequences but their task was to choose among two or four distribution options the one that best matched the distribution of the observed samples (Fig. 4A). Learning of latent structures (i.e., cluster properties) was neither explicitly required by the task itself nor implied in the cover story.

For Experiments 4–7 (*n* = 31, 33, 55, 67, respectively), the distribution set in Experiment 1 was used as generative distributions. Participants observed 20 to 160 samples, presented in groups of five. To prevent participants from using mean or variance as cues for recognition, we set all distribution options in the same trial to identical means and variances (Fig. S5). The number of clusters in the options could vary from 1 to 4. Experiments 4–7 show converging results, despite their differences in the visualization of options or samples, or the number of options (Fig. 4B). In these experiments, although the true distribution was equally likely to be 1, 2, 3, or 4, participants exhibited a clear bias toward options with 2 to 3 clusters, with the frequency of choosing 2 or 3 clusters greater than that of 1 or 4 clusters (*t*-tests, *p*  < 0.001 for all four experiments, Fig. 4B), which is consistent with our findings of illusory structures of 2–3 clusters in Experiments 1 and 2.

With more samples, participants were more likely to correctly identify the target option (Fig. S8A, all *p*  < 0.002 for Experiments 4–7, see Methods for test details). This finding does not necessarily conflict with our finding of the failure of convergence to the ground truth in the reported cluster number in structured density reports (Fig. 1D). As shown in Experiment 1, participants’ overall density representation improved with sample size (Fig. 1B), which may outweigh its discrepancy with the target option in cluster number.

Given only 20 samples, participants demonstrated a striking perception of illusory structures for the Gaussian distribution. For Experiment 7 (two-alternative forced choice), participants had a larger probability of choosing the lure option than the target option (bimodal lure vs. Gaussian, *t*(66) = 3.24, *p* = 0.002, 95%CI = [0.08, 0.33], *d* = 0.40; trimodal lure vs. Gaussian, *t*(66) = 3.13, *p* = 0.003, 95%CI = [0.07, 0.33], *d* = 0.39). Similar illusory patterns were found for experiments with four alternative options: Participants were more likely to choose the trimodal lure over the correct Gaussian option in Experiment 5 (*t*(32) = 3.42, *p* = 0.002, 95%CI = [0.04, 0.15], *d* = 0.60) and the bimodal lure over the Gaussian in Experiment 6 (*t*(54) = 2.50, *p* = 0.015, 95%CI = [0.02, 0.17], *d* = 0.34). The probability of choosing the bimodal lure was also higher than the Gaussian in Experiment 4, though the difference did not reach statistical significance (Fig. S8B–E).

To test whether the structure biases observed in Experiments 4–7 align with a memory-constrained structure expansion process analogous to that of the DEE model, we evaluated three alternative models for the distribution recognition task (see Supplementary Materials for details). All models account for participants’ overall density representation by calculating likelihoods of samples generated by each option distribution, but differ in their assumed structure biases. The DEE and CRP models parallel their counterparts from the structured density estimation task, implementing structure biases based on memory-constrained structure expansion and unbounded structure expansion with samples, respectively. The MLE model assumes no structure biases. Importantly, we used DEE parameters fitted in Experiment 1 to cross-predict structure biases, aiming to explore whether parameters derived from the structured density estimation task can qualitatively describe participants’ choices in the density recognition task. A similar cross-prediction procedure was applied to the CRP model. We found that the DEE model successfully captured participants’ recognition biases toward distributions with 2–3 clusters (Fig. 4B) and provided better fitting of recognition choices compared to the CRP and MLE models (Fig. 4C).

**Fig. 5:**
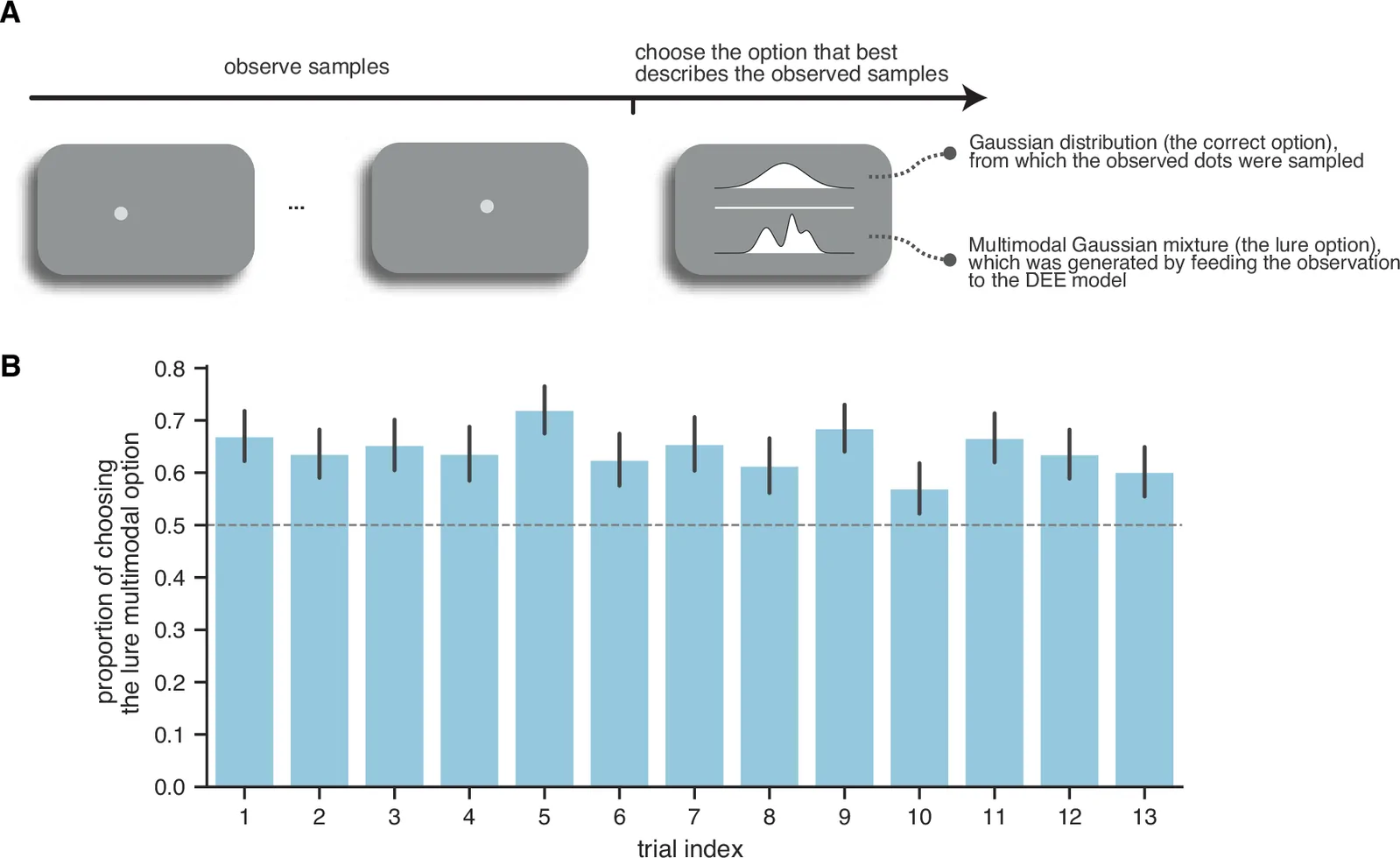
The pre-registered probability density recognition experiment based on DEE-generated options. **A** In Experiment 8, the true generative distribution was always Gaussian. The option distributions were either a single Gaussian or a Gaussian mixture lures generated by the Distorted Economical Expansion (DEE) model based on the presented samples. **B** Participants consistently preferred the DEE-generated lure option over the correct Gaussian option throughout the experiment. Error bars indicate SEM across participants, and the number of participants summarized in each bar varies (from left to right, *n* = 97, 110, 98, 88, 100, 96, 87, 88, 108, 107, 102, 107, 108).

To further test DEE’s predictive power, we performed a pre-registered Experiment 8 (*n* = 216), confronting participants with a DEE-generated lure option alongside the Gaussian distribution that generated the samples (Fig. 5A). To minimize the influence of overall density while highlighting potential structure biases, we matched the likelihoods of samples under the two options to be roughly equal (Fig. S9AB). As hypothesized, participants significantly preferred the DEE-generated lure over the ground truth (*t*(215) = 8.71, *p*  < 0.001, 95%CI = [0.22, 0.35], *d* = 0.59), despite the two having approximately equal likelihoods to generate the samples. This preference was significantly stronger in experimental trials (with carefully likelihood-matched options) compared to control trials where the multimodal lure had substantially lower likelihood (*t*(215) = 5.03, *p*  < 0.001, 95%CI = [0.06, 0.13], *d* = 0.34, Fig. S9C, see Methods for details). Unlike Experiments 4–7, the true generative distribution was always Gaussian in Experiment 8, eliminating the possibility that the observed recognition bias stemmed from exposure to multimodal distributions in the generative distribution set. This bias showed up even in the first trial (Fig. 5B), which adds to the evidence that its origin lies outside the laboratory.

## Discussion

Using a novel structured density task, we revealed the illusory structures in the probability distributions perceived by humans. While participants could approximate the overall density, they often failed to identify the true generative structure, and seeing more observations resulted in an increase in the reported number of clusters. Our results across eight experiments consistently showed that participants favored moderately complex representations, regardless of feature scales, modalities, and downstream tasks. Human performance is best modeled by a distorted economical expansion (DEE) process, where an individual’s internal world model grows incrementally with new observations but at a decreasing rate when the model becomes increasingly complicated. It is as if a conservation mechanism is gradually activated when cognitive resources approach exhaustion.

One striking finding is that humans persistently misinterpret Gaussian distributions as more complex structures, typically seeing them as mixtures of barely overlapping clusters. Though seemingly counterintuitive, this finding does not necessarily conflict with previous studies that suggest humans prefer simpler, unimodal representations^22,29,30^, where multimodal representations (especially those deviating from the ground truth) were not systematically tested, even when the visualized data hint multimodality^30^.

In fact, our findings of overcomplication in learned structures are hinted at in seminal modeling works on category learning^11,12^ and align with findings from a wide range of behavioral paradigms. Some studies^26,35^ ask participants to make decisions based on the integral of probability distributions and use computational models to infer participants’ internal representations, revealing that participants form multimodal representations even for samples from Gaussian-like distributions. Another study^4^ uses computational modeling to infer how participants estimate the mean and variance of samples in dynamic environments and finds that humans overestimate the probability that changes occur—a tendency toward overcomplication since each changepoint introduces a new latent cause. Two recent studies^48,49^ use histogram-like reports to obtain more model-free and richer measures of participants’ probability distribution representations. Through spectral analysis^48^ or classification analysis^49^ on the high-dimensional reports, both studies indicate multimodal features in prior beliefs, at least for some participants. In contrast to these findings that rely on indirect behavioral measures, auxiliary model assumptions, or complicated data analysis methods, our structured density estimation task provides more direct evidence for humans’ illusory perception of latent probabilistic structures. Converging evidence from the distribution recognition task further demonstrates that such illusory structure perception is automatic, not depending on explicit requirements of structure learning.

The biases in the reported structured density more likely stem from the learning process rather than from subsequent task-specific decision-making processes. Participants reported more clusters as the sample size increased, indicating that the bias evolves during learning, which cannot be explained by a single post-learning decision policy. The illusory perception of clusters is observed across both the structured density estimation and distribution recognition tasks, suggesting that the biases have arisen before being revealed in different report formats. In particular, the distribution recognition task tested in Experiments 4–8 required only simple forced-choice judgments that are less affected by report biases. Further, in Experiment 5, the options provided were presented as raw samples comparable to those of the stimulus, making judgments further immune to potential distortions in the decision phase. What is more, our aleatoric component can be regarded as a model for sequential cognitive decisions, and is the best among a few different cluster manipulation strategies. Meanwhile, other rational components could not account for the data as well as the DEE when they were augmented with this aleatoric component. In future studies, the aleatoric component could be extended to include other cognitive processes to further investigate any sequential decision biases, possibly under a task where participants are shown a target density for which they then need to sequentially report.

At first sight, the behavior that the human learners fail to converge to the true latent structure and instead increasingly over-complicates the representations with more observations seems to violate the normative Bayesian learning (i.e., Bernstein-von Mises theorem states that the posterior can converge to the truth with an infinite number of observations^50^). However, this theorem assumes that the observations are drawn from the learner’s prior (well-specifiedness). When there is a mismatch, as in our experiments and likely in real-life scenarios, Bayesian learners may fail to converge to the true structure, as proven in statistical settings similar to our experiments^19,51^.

The DEE model builds on the seminal works on category learning^11,12^. We contribute to this line of research in three aspects. First, we show that structural inconsistencies in online and efficient model learning also manifest in the representation of structured density, a fundamental aspect of cognition that underlies human judgment and decision making^52^, regardless of stimulus scale and modality. Second, we extend the structure extension process to reflect the consequences of limited cognitive resources, by introducing probability distortion^41,42,53^ and economical expansion. Third, our modeling framework addresses the challenge of capturing variable and high-dimensional behavioral reports. It incorporates two methodological innovations: an aleatoric modeling component that validates the likelihood of participants’ responses even when there is a dimensional mismatch between the simulated belief and the observed report, and a GPU-based implementation that enables efficient parallel simulation of noisy belief updates. These features allow us to apply maximum likelihood estimation in an open-ended task without predefined report dimensions, enabling the identification of individual cognitive biases in a highly flexible behavioral task. This modeling framework may also be extended to hypothesis-driven investigations of complex behaviors beyond standard density estimation.

We chose sequential item presentation in our task because it closely mirrors online learning under uncertainty in real-world settings^11^. An intriguing question is whether our major findings—the illusory perception of probabilistic structures and the learning process characterized by the DEE model—can be generalized to simultaneous presentation of samples, where working memory demands are reduced and deliberation on the structure is possible. The findings from a recent study make us conjecture that simultaneous presentation of samples may yield similar illusory perception: when estimating the mean position of a cloud of Gaussian-distributed dots, participants seem to group the dots into multiple clusters^54^, resembling findings for sequentially-presented samples from multimodal distributions in earlier studies^34^. It should be noted that simultaneous presentation does not necessarily entail parallel processing of all samples. For example, even estimating the statistics of a cloud of dots—commonly assumed to involve parallel processing—involves integrating information from sequential eye fixations^55^, with sequential processing being even more likely for non-spatial features (e.g., shapes or numbers). Even when simultaneous presentation involves truly parallel processing, the DEE model could still apply, with minor algorithmic modifications—reframing the sequential random processes into equivalent parallel forms and replacing the particle filter with batch sampling methods such as Gibbs sampling^12^. Our key model assumptions, including economical model expansion with sample size, should still apply because cognitive constraints would continue to limit the number of clusters used to represent distributions for future use, even when individual samples need not be memorized. Future studies are needed to test our conjectures.

One limitation of our study is that we did not manipulate participants’ cognitive capacities, and the decaying expansion of the DEE describes a simple conservation strategy as the internal model complexity grows. This leaves open the question of whether humans can rationally meta-control the "hyperparameter" involved in structure learning. For instance, when learning capacity is severely constrained—such as under stress or limited processing time^56,57^—might the system adapt by prioritizing more general features of the stimulus (e.g., mean and standard deviation), thereby producing a simpler representation? Alternatively, structural expansion might be inherently rigid, leading individuals to maintain a similar number of clusters for representing stimulus density, but at the cost of reduced precision of each cluster property. Future studies that systematically manipulate cognitive resources and task demands are needed to address this question. Such work would help build a more complete mechanistic account of structure learning, one that bridges normative and algorithmic levels of cognitive modeling^58^.

Our computational modeling approach applied to structured density estimation also raises interesting questions for future work. As Sanborn et al.^12^ already noted, single-particle inference on the DEE (or CRP) only allows the model to grow for each new observation, but there is no explicit, step-wise merging operation. We had tested two mechanisms to introduce reduction in the number of clusters, but neither provided a better fit to the data: the first was to allow clusters to merge in the aleatoric component, and the second was to increase the number of particles, which amounts to keeping more than one possible cluster assignment in memory (multiple *z*_1:*t*_ for a single *x*_1:*t*_), leading to more or fewer clusters. On capturing within-participant behavioral variability, although our model could reproduce some of the variability exhibited in human structure learning reasonably (Fig. S3D), residual mismatches remain. For example, the DEE model exhibited greater trial-to-trial variability in cluster number reports than our participants when presented with identical stimulus sequences, suggesting that additional perceptual or cognitive mechanisms may be needed to account for the consistency observed in sequential structure learning. One possibility is that the brain infers structural information by integrating multiple recent observations held in another memory buffer^59^, rather than updating beliefs based solely on the most recent observation. Such multi-observation integration could stabilize the structure learning process and reduce inferential variability. Future studies could query more reports per sequence from participants and vary the sequence length more systematically (e.g., refs. ^37,38^) to differentiate and model these noise components more precisely. To better predict human behavior in our tasks, one could optimize a more flexible but black-box model to the data, such as neural networks potentially with interpretable constraints (e.g., refs. ^60–62^). There, similar aleatoric components as we proposed in this work can be used as the structured noise process for maximum-likelihood training.

Illusory perceptions of hidden causal structures are common in daily life, from harmless superstitions in sports (e.g., rituals believed to influence outcomes^63^) to harmful beliefs with real-world consequences, such as the poaching of rhinoceroses driven by humans’ false medicinal usage. While the scientific recognition of such statistical illusions dates back centuries, a formal understanding of their origins has remained elusive^64–68^. Our empirical findings and modeling point to one perspective: people tend to expand their internal world models to accommodate a drastically open-ended hypothesis space. This tendency may often yield satisfactory predictions within reasonable computation and memory constraints, but not necessarily ensure a truthful understanding of the world.

## Methods

### Participants

Eight experiments were conducted in total. The study was approved by the Institutional Review Board at the School of Psychological and Cognitive Sciences at Peking University. All experiments were conducted in accordance with the terms and regulations specified by the ethical approval. Informed consent was obtained from all participants prior to their participation in the experiment. Experiments 1 to 5 were conducted on site at Peking University. Experiments 6 to 8 were conducted online, with participants recruited from the Prolific platform. All experiments were programmed using PsychoPy^69^. Each experiment recruited a different set of participants. Experiment 1 recruited 21 participants (11 females, aged 18–25). Experiment 2 recruited 21 participants (13 females, aged 18–25). Experiment 3 recruited 36 participants (21 females, aged 18–26), with 18 participants assigned to each scale condition. Experiment 4 recruited 31 participants (21 females, aged 18-28). Experiment 5 recruited 33 participants (18 females, aged 18–26). Experiment 6 recruited 80 participants (20 females, 11 participants did not disclose their gender, aged 20–56). Experiment 7 recruited 80 participants (27 females, 3 participants did not disclose their gender, aged 20–62). Experiment 8 recruited 245 participants (102 females, 1 participant did not disclose their gender, aged 18–76). Participants received monetary compensation (in-person experiments, approximately 50 CNY per hour; online experiments, approximately 8.5 GBP per hour).

Data exclusion: One participant was excluded from Experiment 2 due to their reported distribution mean being insensitive to the true mean, evidenced by a slope less than three standard deviations below the mean slope across participants. For Experiments 6 and 7, three comprehension check questions were included before the experiment began, and participants failing any of these were excluded. As a result, 24 participants were excluded in Experiment 6, and 13 participants were excluded in Experiment 7. Additionally, one participant in Experiment 6 was excluded due to the absence of a confidence report. Experiment 8 followed the pre-registered exclusion criteria (https://doi.org/10.17605/OSF.IO/4SHQD) and excluded 27 participants. Data from 2 participants in Experiment 8 failed to reach the server due to technical errors.

### Experiments 1–3: structured density estimation task

Participants observed a sequence of samples presented on the screen, and were asked to predict the future density by detailing its underlying latent structure. The true distribution varied across trials. The set of true distributions varied across experiments. To ensure that we were assessing participants’ innate inductive biases in distribution learning, no feedback was provided in these experiments. See the Supplementary Materials for details of the instructions used in Experiments 1 and 2.

#### Experiment 1

We created 12 distributions (columns 1–3 of Fig. S2A) using a “merge-from-four” procedure inspired by the split-merge method in Bayesian clustering (see Fig. S5 for details). Each distribution has a standard deviation of about 5.3 cm and an equal probability of containing 1, 2, 3, or 4 clusters. We jittered the distribution mean by a uniform range of -5.3 cm to +5.3 cm for each trial. In half of the trials, we flipped the distributions horizontally (shown as columns 4–6 of Fig. S2A) to balance the overall skewness across the experiment.

On each trial, participants saw either 10 or 70 samples drawn sequentially from one of these distributions. Each sample was visualized as a red dot on screen for 167 ms, followed by a blank screen for 167 ms.

After viewing all the red dots, participants saw a 1-s blank screen before entering the cluster center reporting phase. In this phase, they identified cluster centers by clicking on the horizontal line (step 3 in Fig. 1A). Each click placed a marker at the corresponding location, represented visually by a small volcano-shaped icon. Once participants had marked all perceived cluster centers, they pressed the “F” key to proceed to the report phase. In this phase, participants selected a previously marked cluster by clicking on its icon and then adjusted its weight and standard deviation by moving the mouse. Participants could revisit any previously placed cluster by clicking its volcano icon and readjust both its weight and standard deviation. While cluster means could not be changed once initially placed, participants could effectively remove a cluster by setting its weight to zero. As participants adjusted cluster properties, a blue curve was updated in real time to show the marginal distribution of the Gaussian clusters. Participants were not restricted in the number of clusters they could report, and there were no time limits for their responses.

Each participant completed 12 (number of distributions) * 2 (horizontally flipped or not) * 2 (number of sample size conditions) * 3 (repeats) = 144 trials in total. The number of clusters in the true distribution was equally likely to be 1, 2, 3, or 4.

#### Experiment 2

Experiment 2 was intended to (1) verify our findings using a set of representative distributions from previous studies, and to (2) assess the noise level in human structure learning by examining the consistency of participants’ structured reports.

The distribution set consisted of 14 distributions across four categories–unimodal-symmetric, unimodal-asymmetric, bimodal, and trimodal–with each category containing a similar number of distributions (Fig. S3A). To ensure comparability, all distributions were rescaled to have a standard deviation of 4.8 cm.

From each of the 14 true distributions, we sampled 4 sequences, yielding a total of 56 sample sequences. To balance skewness across the experiment, half (28) of these sequences were horizontally flipped. Each sequence was presented twice, resulting in 112 trials in total. Additionally, the mean of each sequence was jittered within a uniform range of  ± 3.7 cm to introduce variability. Each sample was visualized as a red dot on screen for 581 ms, followed by a blank screen for 167 ms. The reporting procedure was the same as Experiment 1. To control the length of the experiment, we did not manipulate the number of presented samples across trials and fixed the number to 20. Each participant completed 112 trials in total.

Participants’ noise levels in structure learning were assessed by measuring the consistency of their reports when exposed to the same sample sequence twice^37,38^.

#### Experiment 3

Experiment 3 examined whether the observed over-clustering phenomenon was specific to the visual modality or the scale of the distribution. To test this, we replaced visual stimuli with numeric stimuli. Participants learned the probability distribution of these numerical values while following the same cover story as in Experiments 1 and 2.

The true distribution set included three types of unimodal distributions (Fig. 2A): a Gaussian distribution, a left-skewed Gaussian mixture (composed of two Gaussian clusters), and a right-skewed Gaussian mixture, which was created by horizontally flipping the left-skewed one. These three distributions appeared with equal frequency throughout the experiment.

On each trial, participants saw either 10 or 70 samples sequentially presented on the screen as numerical landing coordinates of magic lava stone. Each number appeared for 1.5 s, followed by a 0.5-s blank screen before the next number was shown. After all samples were presented, participants first reported the number of invisible volcanoes they believed to be in the region by entering a value (up to 9) using the keyboard and pressing Enter to confirm.

Based on their reported number of volcanoes, the screen then displayed a corresponding number of input fields, each representing one volcano. Participants entered the location and relative frequency for each reported volcano. Once all inputs were provided, they clicked a confirmation button to complete the trial. The experiment enforced a reporting constraint that the sum of the reported relative frequencies must equal 1. Participants were allowed to revisit the input fields and revise their responses before their explicit confirmation.

Unlike in Experiments 1 and 2, Experiment 3 did not require participants to report cluster width. This decision was made because, without visual information about the distribution’s shape, estimating its width is not intuitive and typically requires extensive practice with feedback to become reliable^35^. Feedback-based cluster-width report training could bring an unwanted effect of influencing participants’ inductive bias of the density, so we decided not to require participants to report the cluster width.

Participants were divided into two groups, each viewing distributions of different scales. In the small-scale group, the standard deviation of all distributions was uniformly rescaled to 100. In the large-scale group, the standard deviation was rescaled to 500. As in Experiments 1 and 2, we introduced random perturbations to the mean of each distribution. The magnitude of this perturbation was proportional to the distribution’s scale. For the large-scale group, the mean was sampled from a uniform distribution in the range [1500, 2500], while for the small-scale group, it was sampled from [300, 500].

Each participant completed 3 (number of distributions) * 2 (number of sample size conditions) * 4 (repeats) = 24 trials in total.

### Experiments 4–8: distribution recognition task

Experiments 1 to 3 revealed rich details about how humans represent uncertainty through explicit structural reports. To assess the robustness of our findings across different downstream tasks, we designed a distribution recognition task.

In this task, participants engaged in a scenario where they had to defend Earth from alien attacks. They observed a sequence of attacks and analyzed which areas were more likely to be targeted in the future. After the observation phase, they were presented with multiple distributions and asked to select the one that best matched the observed attack pattern. No feedback was provided in the main session. We hypothesized that participants would show a similar recognition bias toward multi-modal distribution, similar to the inductive bias exhibited in the structured density estimation task.

#### Experiment 4

On each trial (Fig. 4A), a total of N (20 to 160) gray vertical bars were presented on the computer screen in groups of five across multiple (i.e., N/5) displays. Each display lasted for 167 ms, followed by a blank interval of 86 ms. The horizontal locations of the vertical bars were samples randomly and independently drawn from a specific Gaussian mixture distribution.

After 1000 ms following the presentation of these samples, four options appeared on the screen, shown as shaded probability density plots, one of which corresponded to the true distribution that generated the observed samples. Participants were instructed to choose the option that best matched the distribution of the presented samples by pressing one of four keys on the keyboard that corresponded to the displayed location of the option on the screen. After their choice, participants reported by key press their confidence level of being correct (high vs. low). No feedback was provided.

The true distribution set is the same as that of Experiment 1. It contained 6 subsets (shown as 6 columns in Fig. S2A), half of which (columns 1 to 3) were simply the horizontal flip of the other half (columns 4 to 6). Each subset contains four Gaussian mixture distributions of cluster number 1,2,3, or 4, generated following the “merge-from-four” procedure (Fig. S5). Four distributions within each subset had approximately identical means and SDs. If one distribution was selected as the true distribution, only the subset that it belonged to would be presented as options. Such design of the option ensured that participants couldn’t rely on the obvious aspect of the density curve – mean and SD – to identify the correct option. Instead, they needed to rely on the shape of the density function.

The number of presented samples had 4 levels: 20,40,80, and 160. Each participant completed 4 (number of sample size conditions) * 4 (number of true-cluster-number conditions) * 6 (the subset that the true distribution belonged to) * 4 (repeats) = 384 trials in total.

#### Experiment 5

Experiment 4 presented the probability density functions using continuous curves. To rule out the possibility that participants’ perception of the density functions was influenced by this visualization format, Experiment 5 instead visualized each option using 150 randomly drawn samples from the corresponding distribution (see ref. ^31^, for a similar visualization logic). All other aspects of the experiment remained identical to Experiment 4.

#### Experiment 6

Experiments 4 and 5 presented stimulus distributions at a fixed scale, using gray vertical bars to represent visual samples. To test whether the observed effects depended on this specific visual format, Experiment 6 modified the stimuli by replacing bars with dots and conducted the task online. The online setting introduced natural variability in viewing conditions, such as screen distance and angle. This allowed us to assess whether the findings from Experiments 4 and 5 generalized beyond the controlled laboratory environment.

Since Experiment 6 was conducted online, we streamlined Experiment 4 to reduce its duration while preserving its core design. The number of sample size conditions was reduced to three (20, 60, 160). The number of repetitions per condition was reduced to two. All other aspects of the experimental design remained the same as in Experiment 4.

Each participant completed 3 (number of sample size conditions) * 4 (number of true-cluster-number conditions) * 6 (the subset that the true distribution belonged to) * 2 (repeats) = 144 trials in total.

#### Experiment 7

Experiments 4-6 consistently found that participants tended to recognize learned distributions as having two or three clusters. One possibility is that, when presented with four options (1, 2, 3, and 4 clusters), participants avoided the extremes (1 or 4 clusters) and favored the intermediate choices (2 or 3 clusters). To rule out this selection bias caused by the simultaneous presentation of multiple options, Experiment 7 presented only two option distributions per trial.

The trial procedure was the same as in Experiment 6, except that participants chose between two distribution options instead of four. The true distribution set remained the same as in Experiments 4-6.

On each trial, one distribution was selected as the true distribution, from which *N* samples (20 or 160) were independently drawn and displayed as dots positioned horizontally on the screen. This distribution was also presented as one of the two option distributions. The other lure option was randomly chosen from the same “merge-from-four" subset, which ensured that the lure distribution differed in cluster number but matched the true distribution in mean and standard deviation. As in previous experiments, such a design prevented participants from relying on basic statistical properties and instead required them to infer the shape of the density function.

Each participant completed 2 (number of sample size conditions) * 4 (number of true-cluster-number conditions) * 3 (number of lure-cluster-number conditions) * 6 (the subset that the true distribution belonged to) = 144 trials in total.

#### Experiment 8

Experiment 8 (pre-registered on March 20th 2024, https://doi.org/10.17605/OSF.IO/4SHQD) was aimed at evaluating the DEE model in a distribution recognition task using DEE-generated response options. The true generative distribution was always Gaussian, and each trial presented 50 samples drawn from it. To ensure that the log-likelihood of the presented samples was approximately equal for both the correct Gaussian option and the DEE-generated options, we followed a three-step procedure: (1) We generated 5000 sequences of 50 samples each from a Gaussian distribution. (2) We applied the DEE model to each sequence using the median parameter estimated in Experiment 1 across participants. (3) For each sequence, we computed the log-likelihood difference between the true Gaussian distribution and the corresponding DEE-generated option. We then selected 20 sample-option pairs where this difference was near zero (absolute difference  < 0.2; Fig. S9A illustrates its closeness to zero compared to the chance level). Half of the selected DEE-generated options had three clusters, while the other half had four. Figure S9B shows all selected sample-options.

Each participant completed 13 trials. In six experimental trials, they were presented with a subset of six sample-option pairs from the selected 20 and asked to choose between the multimodal DEE-generated option and the Gaussian distribution. Additionally, six control trials were included, in which the multimodal option was replaced with the one that had the lowest likelihood among the 20 generated alternatives. An instructional comprehension check trial was also included, requiring participants to determine whether the center of the presented samples was positioned on the left or right side of the screen. The order of experimental trials, control trials, and comprehension check trials was random.

We pre-registered two hypotheses. First, in the experimental trials, if participants perceive the DEE-generated multimodal distributions as more representative of the presented samples, they should favor the multimodal option over the true Gaussian. This hypothesis was tested using a one-sample t-test to assess whether the frequency of choosing the multimodal option exceeds the 50% chance level. Second, we expect participants to select the multimodal option more frequently in experimental trials than in control trials. This was tested using a paired t-test comparing choice frequencies between experimental and control trials. Both hypotheses were confirmed. All analyses followed the pre-registered procedure without deviation.

### The DEE, CRP, and likelihood computation

The DEE model comprises a rational component and an aleatoric component, which we detail here. The former expresses the inductive biases participants may have when inferring the density from sequential observations. The latter is a generic noise model over structured densities that allows random mistakes in reporting the structured density; this component is shared among different rational components, including the DEE, the CRP, and the batch-based model.

The rational component infers the structured density given sequential observations under a generative model of how structured densities can produce the observations. The generative model comprises a structure expansion prior and a cluster property prior. The structure expansion prior produces a sequence of cluster assignments denoted by **z**_*t*_:= *z*_1:*t*_, independent of the stimuli **x**_*t*_:= *x*_1:*t*_. Given *K*_*t*_ clusters at time *t*, the assignment *z*_*t*__+1_ takes on values in {1, …, *K*_*t*_ + 1}, with the *K*_*t*_ + 1’th being a newly created cluster. The probability of cluster assignment is

1 $${p}_{{{{\rm{R}}}}}({z}_{t+1}=k| {{{{\bf{z}}}}}_{t}): \! \!=\left\{\begin{array}{ll}\frac{{\tilde{n}}_{t,k}}{t+{\alpha }_{t}},\quad &k\le {K}_{t} \hfill \\ \frac{{\alpha }_{t}}{t+{\alpha }_{t}},\quad &k={K}_{t}+1\\ \quad \end{array}\right.,\quad {\alpha }_{t}: \! \!={\alpha }_{0}{e}^{-r{K}_{t}},\quad {\tilde{n}}_{t,k}: \! \!=t\frac{{n}_{t,k}^{\beta }}{\mathop{\sum }_{k'=1}^{{K}_{t}}{n}_{t,k'}^{\beta }}$$

for *t* ∈ {1, … *T* − 1}, where *z*_1_ = 1, ${n}_{t,k}: \! \!={\sum }_{\tau=1}^{t}{{\mathbf{1}}}[{z}_{\tau }=k]$ is the number of stimuli assigned to cluster *k* at time *t*, and ${K}_{t}: \! \!={\sum }_{k}{{\mathbf{1}}}[{n}_{t,k} > 0]$ is the number of clusters at time *t*. Crucially, we introduced two cognitively plausible inductive biases to the CRP prior. First, while the expansion rate *α* is a constant in CRP, resulting in potentially an infinite number of clusters, we restrict memory load by making it depend on *K*_*t*_. For *r* > 0, new clusters are less likely to be added as the model size grows. Second, while the probability of assigning to a cluster is proportional to the precise cluster size *n*_*t*,*k*_, we posit that this may be distorted following Stevens’ power law^40,41^. The distortion rate *β*≥0 controls whether the effects of the cluster size on new assignments are attenuated (*β* < 1) or exaggerated (*β* > 1), giving a form of divisive normalization. This cluster assignment prior recovers the conventional Chinese restaurant process (CRP) when *r* = 0 and *β* = 1, which has been a popular choice for modeling flexible online learning.

The prior over the initial cluster properties (mean *m* and variance *v*) is the conjugate prior of the Gaussian distribution.

2 $${p}_{{{{\rm{R}}}}}(m,v;{\mu }_{0},{\lambda }_{0},{a}_{0},{\Sigma }_{0})={{{\mathcal{N}}}}(m;{\mu }_{0},v/{\lambda }_{0}){{{\rm{Inv}}}}{\chi }^{2}(v;{a}_{0},{\Sigma }_{0}).$$

The parameters are the prior mean (*μ*_0_) and prior variance (*Σ*_0_), along with their confidences *λ*_0_ and *a*_0_, respectively. Note that we use *Σ* to represent the variance of a cluster, and *σ* the standard deviation. Given a particular sequence of observations **x**_*t*_ and cluster assignments **z**_*t*_, the posterior *p*_R_(*m*, *v*∣**z**_*t*_, **x**_*t*_) is also written in the same form as Equation (2), with parameters efficiently calculated in closed form.

While the structure expansion prior describes how, in the participant’s perspective, observations (spatial dots, numbers, etc.) could arise from a structured density, recognizing the Gaussian clusters and their cluster properties requires statistical inference to invert the generative process. Previous works have established that, for models similar to ours, a particle filter algorithm with a single particle can describe human behavior very well, with little or no improvement when using more than one particle^12,70^. This algorithm iterates over the following step: at each time step *t*, we have computed a particle of previous assignments *z*_1:*t*_  and recorded the count assigned to each *k*’th cluster (*n*_*t*,*k*_). This particle **z**_*t*_ indicates which observation from **x**_*t*_ belongs to which cluster, leading to a posterior over cluster properties for each cluster *p*_R_(*m*_*k*_, *v*_*k*_∣**x**_*t*,*k*_) (where **x**_*t,k*_ means observations assigned to the *k*-th cluster up to time *t*), summarized by posterior statistics ${\hat{{{\varphi }}}}_{t}: \! \!=\{{\mu }_{t,k},{\lambda }_{t,k},{\Sigma }_{t,k},{a}_{t,k}\}_{k=1}^{K_t}$. Now, at time *t* + 1, a new observation *x*_*t*+1_ arrives, we take a sample from the posterior:

3 $${p}_{{{{\rm{R}}}}}({z}_{t+1}| {{{{\bf{z}}}}}_{t},{{{{\bf{x}}}}}_{t},{x}_{t+1})\propto {p}_{{{{\rm{R}}}}}({z}_{t+1}| {{{{\bf{z}}}}}_{t}){p}_{{{{\rm{R}}}}}({x}_{t+1}| {{{{\bf{z}}}}}_{t},{{{{\bf{x}}}}}_{t},{{{{\bf{z}}}}}_{t+1}).$$

The first term is available from (1), and the second term is a Student’s *t* distribution given by $\int\int{{{\mathcal{N}}}}({x}_{t+1};m_k,v_k){p}_{{{{\rm{R}}}}}(m_k,v_k|{{{{\bf{x}}}}}_{t,k}){{{\rm{d}}}}m{{{\rm{d}}}}v$, where ${{{\mathcal{N}}}}$ denotes the normal density function. The new sampled *z*_*t*+1_ is concatenated with **z**_*t*_, forming an updated particle **z**_*t*+1_ for the next iteration.

The procedure above defines the rational component, leading to an estimate of the property clusters $\hat{{{\varphi }}}: \! \!=\hat{{{\varphi }}}_{T}$, as well as an estimated number of clusters $\hat{K}$. The likelihood function on a reported cluster component *φ*_*t*_ requires a non-trivial noise process, which we call the aleatoric component *p*_A_. This component first randomly samples a $\tilde{K}$ from a noisy distribution centered at the estimated $\hat{K}$. Large noise makes it less likely to have $\tilde{K}=\hat{K}$. If $\tilde{K}$ does not match the reported *K* from the participant, then this particle is given a 0 likelihood. The predicted $\hat{K}$ is thus encouraged to match the reported *K*. If $\tilde{K}\ne \hat{K}$ (the participant lapses), then the predicted cluster properties are first modified through a heuristic spliting or dropping operation until the modified structure has $\tilde{K}$ clusters. The modified cluster properties then define a valid likelihood using standard noise models: a Dirichlet distribution for the weights ${w}_{k}\propto {n}_{T,k}^{\beta }$, a normal distribution for the means, and a log-normal distribution for the variances. We averaged this likelihood over a large number of permutations of the clusters to ensure permutation invariance.

Since we do not know which stochastic sample the participants drew in their minds in all the stochastic steps (e.g., noisy observations, *φ* given observations, the lapse in ${\tilde{K}}$), we as the experimenter need to simulate the procedure above many times and average the resulting likelihoods, which is usually very slow. We expedite this by using a GPU-friendly implementation by PyTorch^71^. This then allows us to perform maximum-likelihood parameter estimation using off-the-shelf optimization routines.

### Batch model

As a baseline model, we implemented a batch learning model based on a simple prior over the number of clusters, the variational Gaussian mixture (VGMM)^72^, and model selection. We assume that the participant has a prior distribution over the number of clusters expressed by a Poisson distribution, with a trainable mean parameter, truncated to ${K}_{\max }$ for each experiment. The participant then clusters all observations using the VGMM algorithm for each possible value of $K\in \{1,\ldots,{K}_{\max }\}$, with the same prior over cluster properties as in (2). Then, the participant chooses in their mind the best model (from ${K}_{\max }$ models) according to the likelihood of the samples penalized by the number of clusters. The cluster properties of the chosen model are taken to be $\hat{{{\varphi }}}$ and sent to the aleatoric component for likelihood computation, as for the DEE model. We simulate a large number of this procedure on GPUs to average out all randomness in this forward process (cluster property initializations, noisy observations, and lapses).

### Model fitting

We fitted the model parameters for each participant on all reported data. First, we ran the Nelder-Mead optimization routine 10 times. Each run was started using the best-fitting parameters from the previous run. The parameters of the last run were recorded. We then repeated for a total of 10 rounds under different random seeds, and we took the parameters with the largest log-likelihood. The final log-likelihood was evaluated using 1 million simulations to ensure accuracy.

### Model comparison

We used the Akaike Information Criterion (AIC) for model selection^73^. To visualize model advantage, we calculated each model’s ΔAIC as the difference between its AIC and the minimum AIC among all models for each participant. Additionally, we applied group-level Bayesian model selection to calculate the protected exceedance probability (pxp) as a complementary measure of model advantage, using -AIC/2 as the model score^74–76^.

### Statistical analysis

All ANOVA tests were implemented using the afex package in R, with post hoc comparisons by the emmeans package. The *p* values in each set of post hoc *t*-tests were corrected using the Tukey method. All reported t-tests were two-tailed. Data distribution met the assumptions of the *t*-tests and ANOVA tests. Cohen’s d and generalized eta-squared (${\eta }_{\,{\mbox{G}}\,}^{2}$) were calculated to quantify effect sizes for the t-tests and ANOVA tests. In Experiment 3, one participant was excluded from the ANOVA examining the distance between reported adjacent clusters, as the participant reported only one cluster in all trials of the 10-sample condition. We did not perform formal tests on the assumptions of residuals.

For the regression analyses, we first estimated individual slopes for each participant. These slopes were then tested using *t*-tests–either against zero (for testing the relationship between the accuracy and the number of observed samples in Experiments 4-7) or against each other (for comparing the relationship between sample moments and reported moments in Experiments 1-3).

### Reporting summary

Further information on research design is available in the Nature Portfolio Reporting Summary linked to this article.

## Supplementary information

Supplementary Information

Reporting Summary

Transparent Peer Review file

## Data Availability

All human behavioral data are available on the OSF platform (https://doi.org/10.17605/OSF.IO/NK5Z7). The repository contains raw data, processed data, and fitted model parameters.

## Code availability

The code is available on the OSF platform (https://doi.org/10.17605/OSF.IO/NK5Z7). The repository contains scripts to reproduce all data analyses, model simulations, model fitting, and all figures except Fig. S1.

## References

[1] Knill, D. C. & Richards, W. *Perception as Bayesian Inference* (Cambridge University Press, 1996).

[2] Maloney, L. T. & Zhang, H. Decision-theoretic models of visual perception and action. *Vis. Res.* **50**, 2362–2374 (2010). 20932856 10.1016/j.visres.2010.09.031

[3] Battaglia, P. W., Hamrick, J. B. & Tenenbaum, J. B. Simulation as an engine of physical scene understanding. *Proc. Natl. Acad. Sci. USA*. **110**, 18327–18332 (2013). 24145417 10.1073/pnas.1306572110 PMC3831455

[4] Nassar, M. R., Wilson, R. C., Heasly, B. & Gold, J. I. An approximately Bayesian delta-rule model explains the dynamics of belief updating in a changing environment. *J. Neurosci.* **30**, 12366–12378 (2010). 20844132 10.1523/JNEUROSCI.0822-10.2010 PMC2945906

[5] Behrens, T. E., Woolrich, M. W., Walton, M. E. & Rushworth, M. F. Learning the value of information in an uncertain world. *Nat. Neurosci.* **10**, 1214–1221 (2007). 17676057 10.1038/nn1954

[6] Antonov, G. & Dayan, P. Exploring replay. *Nat. Commun.* **16**, 1657 (2025). 39955280 10.1038/s41467-025-56731-y PMC11829958

[7] Koblinger, Á, Fiser, J. & Lengyel, M. Representations of uncertainty: where art thou? *Curr. Opin. Behav. Sci.* **38**, 150–162 (2021). 34026948 10.1016/j.cobeha.2021.03.009 PMC8121756

[8] Tenenbaum, J. B., Kemp, C., Griffiths, T. L. & Goodman, N. D. How to grow a mind: statistics, structure, and abstraction. *science* **331**, 1279–1285 (2011). 21393536 10.1126/science.1192788

[9] Dayan, P., Hinton, G. E., Neal, R. M. & Zemel, R. S. The Helmhotz machine. *Neural Comput.* **7**, 889–904 (1995). 7584891 10.1162/neco.1995.7.5.889

[10] Higgins, I. et al. beta-vae: Learning basic visual concepts with a constrained variational framework. *International Conference on Learning Representations* (2017).

[11] Anderson, J. R. The adaptive nature of human categorization. *Psychol. Rev.* **98**, 409 (1991).

[12] Sanborn, A. N., Griffiths, T. L. & Navarro, D. J. Rational approximations to rational models: alternative algorithms for category learning. *Psychol. Rev.* **117**, 1144 (2010). 10.1037/a0020511 21038975

[13] Griffiths, T. L., Canini, K. R., Sanborn, A. N. & Navarro, D. J. Unifying rational models of categorization via the hierarchical Dirichlet process. *Proc. Annu. Meet. Cogn. Sci. Soc.* **29**, (2007).

[14] Gershman, S. J. & Niv, Y. Perceptual estimation obeys Occam’s razor. *Front. Psychol.* **4**, 623 (2013). 10.3389/fpsyg.2013.00623 PMC3780620 24137136

[15] Donoso, M., Collins, A. G. & Koechlin, E. Foundations of human reasoning in the prefrontal cortex. *Science* **344**, 1481–1486 (2014). 24876345 10.1126/science.1252254

[16] Collins, A. & Koechlin, E. Reasoning, learning, and creativity: frontal lobe function and human decision-making. *PLoS Biol.* **10**, e1001293 (2012). 22479152 10.1371/journal.pbio.1001293 PMC3313946

[17] Shin, Y. S. & Niv, Y. Biased evaluations emerge from inferring hidden causes. *Nat. Hum. Behav.* **5**, 1180–1189 (2021). 33686201 10.1038/s41562-021-01065-0 PMC8423857

[18] Heald, J. B., Lengyel, M. & Wolpert, D. M. Contextual inference underlies the learning of sensorimotor repertoires. *Nature* **600**, 489–493 (2021). 34819674 10.1038/s41586-021-04129-3 PMC8809113

[19] Miller, J. & Harrison, M. T. A simple example of Dirichlet process mixture inconsistency for the number of components. *Adv. Neural Inform. Process. Syst.* **26**, 199–206 (2013).

[20] Sohn, H. & Jazayeri, M. Validating model-based Bayesian integration using prior–cost metamers. *Proc. Natl. Acad. Sci. USA.* **118**, e2021531118 (2021). 34161261 10.1073/pnas.2021531118 PMC8237636

[21] Laquitaine, S. & Gardner, J. L. A switching observer for human perceptual estimation. *Neuron* **97**, 462–474 (2018). 29290551 10.1016/j.neuron.2017.12.011

[22] Flannagan, M. J., Fried, L. S. & Holyoak, K. J. Distributional expectations and the induction of category structure. *J. Exp. Psychol.: Learn., Mem., Cognit* **12**, 241 (1986). 2939181 10.1037//0278-7393.12.2.241

[23] Whiteley, L. & Sahani, M. Implicit knowledge of visual uncertainty guides decisions with asymmetric outcomes. *J. Vision* **8**, 2–2 (2008). 10.1167/8.3.2 PMC2515365 18484808

[24] Gallistel, C. R., Krishan, M., Liu, Y., Miller, R. & Latham, P. E. The perception of probability. *Psychol. Rev.* **121**, 96 (2014). 24490790 10.1037/a0035232

[25] Courville, A. C., Daw, N. D. & Touretzky, D. S. Bayesian theories of conditioning in a changing world. *Trends Cogn. Sci.* **10**, 294–300 (2006). 16793323 10.1016/j.tics.2006.05.004

[26] Zhang, H., Daw, N. D. & Maloney, L. T. Human representation of visuo-motor uncertainty as mixtures of orthogonal basis distributions. *Nat. Neurosci.* **18**, 1152–1158 (2015). 10.1038/nn.4055 PMC4487408 26120962

[27] Landy, M. S., Trommershäuser, J. & Daw, N. D. Dynamic estimation of task-relevant variance in movement under risk. *J. Neurosci.* **32**, 12702–12711 (2012). 22972994 10.1523/JNEUROSCI.6160-11.2012 PMC3477850

[28] Maloney, L. T. & Mamassian, P. Bayesian decision theory as a model of human visual perception: testing Bayesian transfer. *Vis. Neurosci.* **26**, 147–155 (2009). 19193251 10.1017/S0952523808080905

[29] Nisbett, R. E. & Kunda, Z. Perception of social distributions. *J. Personal. Soc. Psychol.* **48**, 297 (1985). 10.1037//0022-3514.48.2.297 3981397

[30] Tran, R., Vul, E. & Pashler, H. How effective is incidental learning of the shape of probability distributions? *R. Soc. Open Sci.* **4**, 170270 (2017). 28878977 10.1098/rsos.170270 PMC5579092

[31] Acerbi, L., Vijayakumar, S. & Wolpert, D. M. On the origins of suboptimality in human probabilistic inference. *PLoS Comput. Biol.* **10**, e1003661 (2014). 10.1371/journal.pcbi.1003661 PMC4063671 24945142

[32] Körding, K. & Wolpert, D. M. Probabilistic Inference in Human Sensorimotor Processing. *Adv. Neural Inform. Process. Syst.* **16**, 1327–1334 (2003).

[33] Sanborn, A. N. & Beierholm, U. R. Fast and accurate learning when making discrete numerical estimates. *PLoS Comput. Biol.* **12**, e1004859 (2016). 27070155 10.1371/journal.pcbi.1004859 PMC4829178

[34] Sun, J., Li, J. & Zhang, H. Human representation of multimodal distributions as clusters of samples. *PLoS Comput. Biol.* **15**, e1007047 (2019). 10.1371/journal.pcbi.1007047 PMC6534328 31086374

[35] Schustek, P. & Moreno-Bote, R. Instance-based generalization for human judgments about uncertainty. *PLoS Comput. Biol.* **14**, e1006205 (2018). 29864122 10.1371/journal.pcbi.1006205 PMC6002126

[36] Körding, K. & Wolpert, D. M. The loss function of sensorimotor learning. *Proc. Natl. Acad. Sci. USA*. **101**, 9839–9842 (2004). 10.1073/pnas.0308394101 PMC470761 15210973

[37] Drugowitsch, J., Wyart, V., Devauchelle, A.-D. & Koechlin, E. Computational precision of mental inference as critical source of human choice suboptimality. *Neuron*. **92**, 1398–1411 (2016). 27916454 10.1016/j.neuron.2016.11.005

[38] Findling, C., Skvortsova, V., Dromnelle, R., Palminteri, S. & Wyart, V. Computational noise in reward-guided learning drives behavioral variability in volatile environments. *Nat. Neurosci.* **22**, 2066–2077 (2019). 31659343 10.1038/s41593-019-0518-9

[39] Pitz, G. F. The sequential judgment of proportion. *Psychonomic Sci.* **4**, 397–398 (1966).

[40] Spence, I. Visual psychophysics of simple graphical elements. *J. Exp. Psychol.: Hum. Percept. Perform.* **16**, 683 (1990). 2148585 10.1037//0096-1523.16.4.683

[41] Stevens, S. S. Neural events and the psychophysical law. *Science* **170**, 1043–1050 (1970). 5475633 10.1126/science.170.3962.1043

[42] Tversky, A. & Kahneman, D. Advances in prospect theory: Cumulative representation of uncertainty. *J. Risk Uncertain.* **5**, 297–323 (1992).

[43] Lloyd, K. & Leslie, D. S. Context-dependent decision-making: a simple bayesian model. *J. R. Soc. Interface* **10**, 20130069 (2013). 23427101 10.1098/rsif.2013.0069 PMC3627089

[44] Gershman, S. J., Blei, D. M. & Niv, Y. Context, learning, and extinction. *Psychol. Rev.* **117**, 197 (2010). 10.1037/a0017808 20063968

[45] Bruijns, S. A. et al. Infinite hidden Markov models can dissect the complexities of learning. *Nat. Neurosci.* **29**, 186–194 (2026). 10.1038/s41593-025-02130-x PMC12779568 41469444

[46] Miller, J. & Harrison, M. T. Mixture models with a prior on the number of components. *J. Am. Stat. Associat.* **113**, 340–356 (2018). 10.1080/01621459.2016.1255636 PMC6035010 29983475

[47] Richardson, S. & Green, P. J. On bayesian analysis of mixtures with an unknown number of components (with discussion). *J. R. Stat. Soc. Ser. B Stat. Methodol.* **59**, 731–792 (1997).

[48] Nie, S., Wang, M. & Zhang, H. Illusory bimodality in repeated reconstructions of probability distributions. *Proc. Annu. Meet. Cogn. Sci. Soc.* 1306–1312 (2021).

[49] DiBerardino, P. A. V., Filipowicz, A. L. S., Danckert, J. & Anderson, B. Plinko: Eliciting beliefs to build better models of statistical learning and mental model updating. *Br. J. Psychol.* **115**, 759–786 (2024). 39096484 10.1111/bjop.12724

[50] Van der Vaart, A. W. *Asymptotic Statistics*, Vol. 3 (Cambridge University Press, 2000).

[51] Lawless, C., Arbel, J. & Alamichel, L. Clustering inconsistency for Pitman–Yor mixture models with a prior on the precision but fixed discount parameter. *Fifth Symposium on Advances in Approximate Bayesian Inference* (2023).

[52] Griffiths, T. L., Chater, N. & Tenenbaum, J. B. *Bayesian Models of Cognition: Reverse Engineering the Mind* (MIT Press, 2024).

[53] Zhang, H., Ren, X. & Maloney, L. T. The bounded rationality of probability distortion. *Proc. Natl. Acad. Sci. USA*. **117**, 22024–22034 (2020). 32843344 10.1073/pnas.1922401117 PMC7486738

[54] Ota, K., Wu, Q., Mamassian, P. & Maloney, L. T. Given the birds, where is the flock? visual estimation of the location of collections of points. *bioRxiv* 2025–07 (2025).

[55] He, L., Wang, H., Bian, Y., Zhang, X. & Bhatia, S. Information sampling and Bayesian belief formation in statistical judgment. *Proc. Natl. Acad. Sci.* **122**, 10.1073/pnas.2517302122 (2025). PMC12557805 41091755

[56] Otto, A. R., Gershman, S. J., Markman, A. B. & Daw, N. D. The curse of planning: dissecting multiple reinforcement-learning systems by taxing the central executive. *Psychol. Sci.* **24**, 751–761 (2013). 23558545 10.1177/0956797612463080 PMC3843765

[57] Otto, A. R., Raio, C. M., Chiang, A., Phelps, E. A. & Daw, N. D. Working-memory capacity protects model-based learning from stress. *Proc. Natl. Acad. Sci. USA*. **110**, 20941–20946 (2013). 24324166 10.1073/pnas.1312011110 PMC3876216

[58] Lieder, F. & Griffiths, T. L. Resource-rational analysis: Understanding human cognition as the optimal use of limited computational resources. *Behav. Brain Sci.* **43**, e1 (2020). 10.1017/S0140525X1900061X 30714890

[59] Radulescu, A., Niv, Y. & Daw, N. D. A particle filtering account of selective attention during learning. *Conference on Cognitive Computational Neuroscience* (2019).

[60] Dezfouli, A., Griffiths, K., Ramos, F., Dayan, P. & Balleine, B. W. Models that learn how humans learn: The case of decision-making and its disorders. *PLoS Comput. Biol.* **15**, e1006903 (2019). 10.1371/journal.pcbi.1006903 PMC6588260 31185008

[61] Miller, K., Eckstein, M., Botvinick, M. & Kurth-Nelson, Z. Cognitive model discovery via disentangled RNNs. *Adv. Neural Inform. Proces. Syst.* **36**, 61377–61394 (2023).

[62] Ji-An, L., Benna, M. K. & Mattar, M. G. Discovering cognitive strategies with tiny recurrent neural networks. *Nature* **644**, 993–1001 (2025). 10.1038/s41586-025-09142-4 PMC12390849 40604278

[63] Bleak, J. L. & Frederick, C. M. Superstitious behavior in sport: Levels of effectiveness and determinants of use in three collegiate sports. *J. Sport Behav.* **21**, 1 (1998).

[64] Skinner, B. F. ’Superstition’in the pigeon. *J. Exp. Psychol.* **38**, 168 (1948). 18913665 10.1037/h0055873

[65] Miller, J. & Gelman, A. Laplace’s theories of cognitive illusions, heuristics and biases. *Stat. Sci.* **35**, 159–170 (2020).

[66] Jin, Y., Jensen, G., Gottlieb, J. & Ferrera, V. Superstitious learning of abstract order from random reinforcement. *Proc. Natl. Acad. Sci. USA*. **119**, e2202789119 (2022). 35998221 10.1073/pnas.2202789119 PMC9436361

[67] Risen, J. L. Believing what we do not believe: acquiescence to superstitious beliefs and other powerful intuitions. *Psychol. Rev.* **123**, 182 (2016). 26479707 10.1037/rev0000017

[68] Laplace, P.-S. *Pierre-Simon Laplace Philosophical Essay on Probabilities: Translated from the Fifth French Edition of 1825 with Notes by the Translator*, vol. 13 (Springer Science & Business Media, 2012).

[69] Peirce, J. et al. Psychopy2: experiments in behavior made easy. *Behav. Res. methods* **51**, 195–203 (2019). 30734206 10.3758/s13428-018-01193-y PMC6420413

[70] Vul, E., Goodman, N., Griffiths, T. L. & Tenenbaum, J. B. One and done? optimal decisions from very few samples. *Cogn. Sci.* **38**, 599–637 (2014). 24467492 10.1111/cogs.12101

[71] Paszke, A. et al. Pytorch: an imperative style, high-performance deep learning library. *Adv. Neural Inform. Process. Syst.* **32**, 8024 (2019).

[72] Blei, D. M. & Jordan, M. I. Variational inference for Dirichlet process mixtures. *Bayesian Anal.* **1**, 121 – 143 (2006).

[73] Akaike, H. Information theory and an extension of the maximum likelihood principle. *Selected papers of Hirotugu Akaike* 199–213 (1998).

[74] Daunizeau, J., Adam, V. & Rigoux, L. VBA: a probabilistic treatment of nonlinear models for neurobiological and behavioural data. *PLoS Comput. Biol.* **10**, e1003441 (2014). 24465198 10.1371/journal.pcbi.1003441 PMC3900378

[75] Rigoux, L., Stephan, K. E., Friston, K. J. & Daunizeau, J. Bayesian model selection for group studies–revisited. *Neuroimage* **84**, 971–985 (2014). 24018303 10.1016/j.neuroimage.2013.08.065

[76] Stephan, K. E., Penny, W. D., Daunizeau, J., Moran, R. J. & Friston, K. J. Bayesian model selection for group studies. *Neuroimage* **46**, 1004–1017 (2009). 19306932 10.1016/j.neuroimage.2009.03.025 PMC2703732

