# Supplementary material for: Human learning of probability distributions is biased toward moderate structural complexity
Source: Nat Commun. 2026 Aug 8;17:9528. doi: 10.1038/s41467-026-76247-3 (PMC13547380; doi:10.1038/s41467-026-76247-3)
Supplement: Supplementary file 1 [file 41467_2026_76247_MOESM1_ESM.pdf]

Supplementary Information

Human learning of probability distributions is biased toward moderate structural complexity

Tianyuan Teng, Li Kevin Wenliang, Hang Zhang

1 Mathematical model

Here we provide a detailed description of the computational framework used to model participants' behaviors. In each trial, given a sequence of stimuli (visual locations or numeric literals) $\mathbf{x}_T := [x_1, x_2, \ldots, x_T]$, the participant reports a Gaussian mixture in terms of the mixing weights, cluster means, and cluster variances, denoted by $\boldsymbol{\varphi} := \{w_k, \mu_k, \Sigma_k\}_{k=1}^K$ where the variables are defined on mixed domains: $K \in \mathbb{N}_+$, $w_{1:K} \in \Delta^K$ (the K-probability simplex), $\mu_k \in \mathbb{R}\, \forall k \in \{1, \ldots, K\}$, and $\Sigma_k \in \mathbb{R}^+ \; \forall k \in \{1, \ldots, K\}$ are the variances. Note that the k'th component has properties described by the triplet (w_k, μ_k, Σ_k), and all K triplets of the Gaussian mixture are collected as an unordered set, since the Gaussian mixture is invariant to the order of the cluster properties. A model that captures the participant's behavior is denoted by a conditional distribution $p(\boldsymbol{\varphi}|\mathbf{x}_T)$. We fit the parameters of p to the data from each participant to maximize the log-likelihood $\log p(\{w_k, \mu_k, \Sigma_k\}_{k=1}^K|\mathbf{x}_{1:T})$.

We proceed by decomposing p into two components: a core component p_{R} that produces an estimate of the cluster properties $\hat{\boldsymbol{\varphi}}$ that is internal to the participants, and a structured noise process p_{A} that describes the noisy report process:

$$p(\boldsymbol{\varphi}|\mathbf{x}_T) = \int p_{\mathrm{A}}(\boldsymbol{\varphi}|\hat{\boldsymbol{\varphi}})\mathrm{d}p_{\mathrm{R}}(\hat{\boldsymbol{\varphi}}|\mathbf{x}_T) \tag{S1}$$

The rational component p_{R} encapsulates our hypotheses about how participants perform structured density estimation; in our approach, we consider inference processes induced by rational generative models; these rational models express our belief about how participants think $\mathbf{x}$ is sampled from Gaussian mixtures that are in turn distributed according to some prior (over Gaussian mixtures). As such, we refer to this core component as the *rational* component.

The noise process p_{A} describes the noisy process by which a participant reports their internal estimates $\hat{\boldsymbol{\varphi}}$. It also captures the variability in the data that cannot be described by the model. As in most other behavioral models, this noise process is often necessary for ensuring valid likelihood functions—it needs to be nonzero everywhere on the data points. Also, the noise model ensures smooth parameter optimization when using local search-based routines (useful when the model has a handful of parameters). For example, we often assume additive Gaussian noise for continuous reported variables. During optimization, the noise scale usually shrinks as the model better captures the data. For discrete reported variables, such as the number of clusters K,

one could assume a lapse mechanism whereby participants occasionally report a random K; the probability of lapse also decreases as the optimization proceeds. In our case, the reported $\boldsymbol{\varphi}$ is far more complicated than the two simple cases above, and the noise process must be more involved.

We now describe the rational component and the aleatoric component in more detail.

1.1 Rational component

We choose p_{R} as the posterior (inference) distribution implied by a generative model that produces samples from Gaussian mixture distributions.

Our main rational component is the distorted economical expansion (DEE) process; it assumes that the participant possesses a prior over Gaussian mixture distributions, which consists of a cluster assignment prior, and a cluster distribution prior.

The cluster assignment prior describes the process by which each stimulus is assigned to an existing or a new cluster, without knowing the values of the stimuli. If the number of clusters at time t is K_t, then the assignment z_{t+1} of the incoming stimulus takes on values in $\{1, \ldots, K_t + 1\}$, with the $K_t + 1$'th being a newly created cluster.

$$p_{\mathrm{R}}(z_{t+1} = k | \mathbf{z}_t) := \begin{cases} \frac{\tilde{n}_{t,k}}{t+\alpha_t}, & k \leq K_t \\ \frac{\alpha_t}{t+\alpha_t}, & k = K_t + 1 \end{cases}, \quad \alpha_t := \alpha_0 e^{-rK_t}, \quad \tilde{n}_{t,k} := t \frac{n_{t,k}^{\beta}}{\sum_{k'=1}^{K_t} n_{t,k'}^{\beta}} \tag{S2}$$

for $t \in \{1, \ldots T-1\}$, where $z_1 = 1$, $n_{t,k} := \sum_{\tau=1}^{t} \mathbb{1}[z_\tau = k]$ is the number of observations assigned to cluster k at time t, and $K_t := \sum_k \mathbb{1}[n_{t,k} > 0]$ is the number of clusters at time t. The expansion rate α_t depends on K_t; for $r > 0$, new clusters are less likely to be added as the model size grows. The distortion rate $\beta \geq 0$ controls whether the effects of cluster size $n_{t,k}$ on new assignments are attenuated ($\beta < 1$) or exaggerated ($\beta > 1$), a form of divisive normalization. This cluster assignment prior recovers the conventional Chinese restaurant process (CRP) when $r = 0$ and $\beta = 1$.

Our cover story effectively informed our participants that each cluster follows a Gaussian-shaped density. Given a particular assignment $\mathbf{z}_t$, a simple method to obtain a Gaussian mixture is to estimate the weights, means and variances (cluster properties) by simple averages, e.g.

$$w_{k,t} = \frac{n_{t,k}}{t}, \quad m_{t,k} = \frac{\sum_{\tau=1}^{t} \mathbb{1}[z_\tau = k] x_\tau}{n_{t,k}}, \quad v_{t,k} = \frac{\sum_{\tau=1}^{t} \mathbb{1}[z_\tau = k](x_\tau - m_{t,k})^2}{n_{t,k}}.$$

However, humans typically do not estimate such quantities accurately and may exhibit uncertainty and bias in estimating these cluster properties.

We thus posit that participants have a prior over the mean and variance of Gaussian distributions for each cluster, and a common choice is the normal-inverse-χ^2:

$$p_{\mathrm{R}}(m, v) = \mathcal{N}(m; \mu_0, v/\lambda_0) \mathrm{Inv}\chi^2(v; a_0, \Sigma_0), \tag{S3}$$

where hyperparameters with subscript 0 follow standard definitions (e.g., [1]). These hyperparameters are interpretable and can capture some aspects of the bias and uncertainty of our participants in estimating the cluster properties. Specifically, $\mu_0 \in \mathbb{R}$ is the prior cluster mean, and $\lambda_0 > 0$ is the belief strength of the prior mean (how strong the prior belief is about the cluster mean); similarly, $\Sigma_0 > 0$ is the prior cluster variance, and $a_0 > 0$ is the belief strength of the prior cluster variance. We also use σ_0 to denote the prior cluster standard deviation. Combining (S3) and the cluster assignment prior (S2), the DEE obtains a generative model of Gaussian distributions, which we take as the participant's prior belief of Gaussian distributions that can produce the observed samples (visual positions or numeric literals). The DEE can thus be seen as a hierarchical and sequential generative model: it first generates Gaussian mixture distributions and cluster assignments, and the observations are obtained by sampling from the generated Gaussian mixture according to the assignments. Once conditioned on a particular set of samples $\mathbf{x}_T$, the posterior belief about which Gaussian mixtures likely produced these samples shrinks to some candidates. The exact computation to obtain the posterior is intractable, so we employ an approximation to capture how participants build an internal model of the observations, which is detailed below.

Following previous modeling work [1–3], we assume humans use particles to approximate the Bayesian update of their density model. A particle is a concrete hypothesis of the latent variables. Unlike samples, particles are not directly drawn from the intractable posterior, but can be regarded as an "approximate samples" influenced by both the prior and observations through the Bayes rule, and eventually converge to true samples at the large-sample limit. In addition, it has also been shown that a single particle usually suffices to model human behaviors [4]. As such, we postulate that participants approximate the posterior of the cluster assignment random variable $\mathbf{z}$ given observations by a single particle. At each time step t, a particle consists of a concrete cluster assignment $\mathbf{z}_t^i = [z_1^i, \ldots, z_t^i]$, where i indicates a concrete sample drawn from the underlying random variable $\mathbf{z}_t|\mathbf{x}_t$ incurred by the particle filtering algorithm. This particle is then sequentially extended to $\mathbf{z}_{t+1}^i = [\mathbf{z}_t^i, z_{t+1}^i]$ given the next observation up to time T. Different runs of the particle filter over a given sequence may produce different cluster assignments, which are used for likelihood approximation; see Section *Monte Carlo simulation* later for details. Thus, we also use the superscript i to indicate the i'th single-particle sequence from a collection of particle filter runs $\mathbf{z}_T^1, \mathbf{z}_T^2, \ldots$.

A sequence of committed cluster assignments $\mathbf{z}_t^i$ induces a posterior over the cluster properties. Specifically, the observations $\mathbf{x}_t$ up to time t and a corresponding particle sequence $\mathbf{z}_t^i$ define a structured density model: a mixture of $K_t^i = \sum_k \mathbb{1}[n_{t,k}^i > 0]$ clusters each of which is weighted proportional to $n_{t,k}^i = \sum_{\tau=1}^t \mathbb{1}[z_\tau^i = k]$ and has posterior parameters given by the usual conjugate update formulae:

$$
\begin{aligned}
p_{\mathrm{R}}(m_k, v_k|\mathbf{x}_t, \mathbf{z}_t^i) &= \mathcal{N}(m_k; \mu_{t,k}^i, v_k/\lambda_{t,k}^i)\mathrm{Inv}\chi^2(v_k; a_{t,k}^i, \Sigma_{t,k}^i), \\
a_{t,k}^i = a_0 + n_{t,k}^i, \quad \lambda_{t,k}^i &= \lambda_0 + n_{t,k}^i, \quad \mu_{t,k}^i = (\lambda_0\mu_0 + n_{t,k}^i\bar{\mu}_{t,k}^i)/\lambda_{t,k}^i, \\
\Sigma_{t,k}^i &= \frac{1}{a_{t,k}^i}\left[a_0\Sigma_0 + n_{t,k}^i\bar{\Sigma}_{t,k}^i + \frac{\lambda_0 n_{t,k}^i}{\lambda_0 + n_{t,k}^i}(\mu_0 - \bar{\mu}_{t,k}^i)^2\right],
\end{aligned} \tag{S4}
$$

where $\bar{\mu}^i_{t,k}$ and $\bar{\Sigma}^i_{t,k}$ are, respectively, the empirical mean and the (unadjusted, biased) empirical variance of the observations assigned to cluster k at time t, according to the particle $\mathbf{z}^i_t$. (S4) shows that, given $\mathbf{z}^i_t$, the posterior of the k'th cluster is determined by $\hat{\boldsymbol{\varphi}}^i_t := [n^i_{t,k}, \mu^i_{t,k}, \Sigma^i_{t,k}]^{K^i_t}_{k=1}$, in the sense that the conditioning variables are all functions of $\hat{\boldsymbol{\varphi}}^i_t$. So, we can equivalently write $p_{\mathrm{R}}(m_k, v_k|\mathbf{x}_t, \mathbf{z}^i_t) = p_{\mathrm{R}}(m_k, v_k; \hat{\boldsymbol{\varphi}}^i_t)$ in (S4). At time step $t+1$, the new observation x_{t+1} is assigned to a cluster according to the "one-step" posterior

$$\begin{aligned} p_{\mathrm{R}}(z^i_{t+1}|\mathbf{z}^i_t, \mathbf{x}_t, x_{t+1}) &\propto p_{\mathrm{R}}(z^i_{t+1}|\mathbf{z}^i_t) p_{\mathrm{R}}(x_{t+1}|\mathbf{z}_t, z_{t+1}, \mathbf{x}_t) \\ &= p_{\mathrm{R}}(z^i_{t+1}|\mathbf{z}^i_t) p_{\mathrm{R}}(x_{t+1}|\hat{\boldsymbol{\varphi}}^i_t, z_{t+1}) \end{aligned} \tag{S5}$$

for $z^i_{t+1} \in [1, \ldots, K^i_t + 1]$, where

$$p_{\mathrm{R}}(x_{t+1}|\hat{\boldsymbol{\varphi}}^i_t, z_{t+1} = k) = \int\int \mathcal{N}(x_{t+1}|m_k, v_k) p_{\mathrm{R}}(m_k, v_k; \hat{\boldsymbol{\varphi}}^i_t) \mathrm{d}m_k \mathrm{d}v_k \tag{S6}$$

is the likelihood of x_{t+1} under cluster k, given by the density of Student's t with degrees of freedom $a^i_{t,k}$, location $\mu^i_{t,k}$ and scale $(\Sigma^i_{t,k}(1 + 1/\lambda^i_{t,k}))^{1/2}$, evaluated at x_{t+1}. If $z_{t+1} \le K^i_t$, then the z_{t+1}'th component is updated by x_{t+1}; if $z_{t+1} = K^i_t + 1$, then a new cluster is created, and the internal construct expands; in both cases, the updates follow (S4). Pausing to take stock, the inference procedure above iterates over stochastic assignment of x_t to z_t given $\boldsymbol{\varphi}^i_t$ and conjugate update of cluster properties in terms of $\boldsymbol{\varphi}^i_t$ given $\mathbf{z}_t$ and $\mathbf{x}_t$.

At the last time step T, we define the last cluster statistics as $\hat{\boldsymbol{\varphi}}^i := \hat{\boldsymbol{\varphi}}^i_T$ and $\hat{K}^i = K^i_T$, which summarize the cluster properties. The cluster weights can be computed using n^i_T. Note that a single particle $\mathbf{z}_T$ results in no uncertainty in the cluster weights. The cluster properties follow the normal-inverse-χ^2 distribution as (S4). We assume that the participant estimates the mean and variance of the k'th cluster as $\mu^i_{T,k}$ and $\Sigma^i_{T,k}$. Thus, the estimated cluster properties are $\hat{\boldsymbol{\varphi}}^i := [n^i_{T,k}, \mu^i_{T,k}, \Sigma^i_{T,k}]^{\hat{K}^i}_{k=1}$, which is regarded as a particle (approximating samples from $p_{\mathrm{R}}(\hat{\boldsymbol{\varphi}}|\mathbf{x}_T)$) passed to the aleatoric component p_{A} in (S1). Later on, we will denote the standard deviation of a cluster by $\sigma = \sqrt{\Sigma}$.

By assuming that the participant uses a single particle for $\mathbf{z}_T$, this procedure is a very coarse approximation compared to exact Bayesian inference. It generates a single hypothesis over cluster assignments of the past, while randomly assigning the incoming stimulus x_{t+1} according to (S5). As such, previous assignments cannot be modified, even if new observations may favor other sequences of assignments, as noted previously by [1]; also, the mixture model can only expand, and there is no mechanism for merging or removing existing clusters. However, this does not imply that single particle inference necessarily leads to under- or over-estimation of the number of clusters. Maintaining multiple particles allows revision of past assignments when new stimuli arrive, potentially reducing the model size; but, we did not find this to improve the fit to human data using DEE or CRP. This may not be surprising given the findings by [5], who showed a simple example where the posterior over the number

of clusters may not converge toward the ground-truth, even using exact inference (an infinite number of particles) and in the limit of infinite observations ($T \to \infty$).

1.2 Aleatoric component

The purpose of the aleatoric component is to construct a well-defined likelihood function by allowing randomness in reporting cluster properties on top of the estimate $\hat{\varphi}$ in the participant's mind. In earlier model fitting, we found that, for some participants, fitting the rational component alone can end up in a regime where the estimated number of clusters $\hat{K}$ almost never matches the reported value, despite the randomness in cluster assignment particles. The likelihood of data then became zero stochastically during optimization. It thus became crucial to have a noise process to obtain more robust and stable likelihood evaluation.

Compared to most other cognitive models, the difficulty here is the non-unique dimensionality of and inter-dependencies within the behavioral variable: the dimensionality depends on $\hat{K}$ which necessarily affects cluster weights, means and variances. Further, the cluster components are unordered sets, which require special care. As such, we incorporate several processing stages in the aleatoric component to randomly modify the estimated $\hat{\boldsymbol{\varphi}}^i$.

First, the participant "lapses" and commits to reporting $\tilde{K}^i$ clusters according to:

$$p_{\mathrm{A}}(\tilde{K}^i|\hat{\boldsymbol{\varphi}}^i) = \begin{cases} 1-\epsilon, & \tilde{K}^i = \hat{K}^i \ ; \\ \epsilon/(K_{\max}-1), & \tilde{K}^i \neq \hat{K}^i \ . \end{cases} \tag{S7}$$

where $\epsilon > 0$ is a lapse parameter, and $K_{\max}$ is the maximum number of reported clusters seen in an experiment across participants.

Second, we assume that participants modify cluster properties to $\tilde{K}$ clusters while keeping the overall distribution roughly the same, giving lapsed properties $\tilde{\boldsymbol{\varphi}}$.

If the participant has not lapsed, then they set $\tilde{\boldsymbol{\varphi}}^i = \hat{\boldsymbol{\varphi}}$. If the participant has lapsed and $\tilde{K}^i \neq \hat{K}^i$, we define cluster modification function $f : (\hat{\boldsymbol{\varphi}}^i, \tilde{K}^i) \mapsto \tilde{\boldsymbol{\varphi}}^i$ which removes or splits existing clusters recursively to obtain a set of lapsed cluster properties until there are $\tilde{K}^i$ clusters as they have committed to:

1. As long as $\tilde{K}^i < \hat{K}^i$, we remove the cluster with the smallest sample count $n^i_{T,k}$. An alternative is to merge the smallest cluster into its nearest neighbor, updating the cluster properties of the neighbor by moment matching. We found that the removal strategy produced better AIC than the merging strategy.
2. As long as $\tilde{K}^i > \hat{K}^i$, we take the cluster with the largest weight and split it into two clusters on either side of the original cluster. The new clusters are centered at equal distances from the original cluster, and their variance is a scaled version of the variance of the original cluster. Specifically, denote the properties of this cluster to be split by $[w, m, \sigma]$ ($\sigma = \sqrt{\Sigma}$ is the standard deviation), we set the cluster properties of the new clusters to be

$$\left[\frac{w}{2},\ m - b_\sigma \cdot \sigma,\ \ s_\sigma \cdot \sigma\right], \quad \left[\frac{w}{2},\ m + b_\sigma \cdot \sigma,\ \ s_\sigma \cdot \sigma\right]. \tag{S8}$$

where $b_\sigma > 0$ and $0 \leq s_\sigma \leq 1$ are free parameters to be optimized. We also tested a version of this strategy where these two parameters are fixed at $b_\sigma = \frac{\sqrt{3}}{2}$ and $s_\sigma = 1/2$, but this gave worse AIC.

The two recursive processes above ensure that $\tilde{\boldsymbol{\varphi}}^i$ now has the same number of clusters as the committed $\tilde{K}^i$. Even if the rational component cannot estimate K in the data, the lapse mechanism now puts more even probabilities on all possible reported values of K, ensuring a more robust and stable likelihood computation. If the rational component alone is sufficient to explain the data, then the optimal lapse variable ϵ is zero.

The likelihood of the reported $\boldsymbol{\varphi}$ in the data given lapsed cluster properties $\tilde{\boldsymbol{\varphi}}^i$ is defined as

$$p_{\mathrm{A}}(\boldsymbol{\varphi}; \tilde{\boldsymbol{\varphi}}^i) = \begin{cases} 0, & \text{if } \tilde{K}^i \neq K; \\ \frac{1}{|\mathcal{S}_K|} \sum_{\pi \in \mathcal{S}_K} p_w(\mathbf{w}; \pi(\tilde{\mathbf{n}}^i)) p_\mu(\boldsymbol{\mu}; \pi(\tilde{\boldsymbol{\mu}}^i)) p_\sigma(\boldsymbol{\sigma}; \pi(\tilde{\boldsymbol{\sigma}}^i)) & \text{if } \tilde{K}^i = K, \end{cases} \tag{S9}$$

where $\mathcal{S}_K$ is the set of all permutations of K elements; and the noise distributions for the three cluster properties are

$$p_w(\mathbf{w}; \tilde{\mathbf{n}}) = \mathrm{Dirichlet}(\mathbf{w}; c(\tilde{\mathbf{n}} + 1)), \tag{S10}$$

$$p_\mu(\boldsymbol{\mu}; \tilde{\boldsymbol{\mu}}) = \prod_{k=1}^{\tilde{K}} \mathcal{N}(\mu_k; \tilde{\mu}_k, \sigma_m), \tag{S11}$$

$$p_\sigma(\boldsymbol{\sigma}; \tilde{\boldsymbol{\sigma}}) = \prod_{k=1}^{\tilde{K}} \log \mathcal{N}(\sigma_k; \tilde{\sigma}_k, \sigma_v). \tag{S12}$$

where $\tilde{K}$ is implied from $\tilde{\varphi}$. The addition by 1 in (S10) ensures that the mode of the Dirichlet distribution is proportional $\tilde{\mathbf{n}}$ when $c = 1$. The scaling parameter c changes the confidence. The variances σ_μ and σ_ν are free parameters.

Note that if $\tilde{K}^i \neq K$, there is an infinite penalty for inferring the number of clusters wrong, so $\tilde{K}^i$ is encouraged to be correct. In turn, this encourages the rational component to infer the correct number of clusters and pushes the tendency of random lapse ϵ to zero.

Overall, the aleatoric component generates lapsed cluster properties $\tilde{\boldsymbol{\varphi}}$ which parametrize the likelihood function in (S9). By averaging the latent variable $\tilde{K}^i$, we arrive at the final expression of the aleatoric component,

$$p_{\mathrm{A}}(\boldsymbol{\varphi} | \hat{\boldsymbol{\varphi}}^i) = \sum_{\tilde{K}=1}^{K_{\max}} p_{\mathrm{A}}(\boldsymbol{\varphi}; f(\hat{\boldsymbol{\varphi}}^i, \tilde{K})) p_{\mathrm{A}}(\tilde{K} | \hat{\boldsymbol{\varphi}}^i) \tag{S13}$$

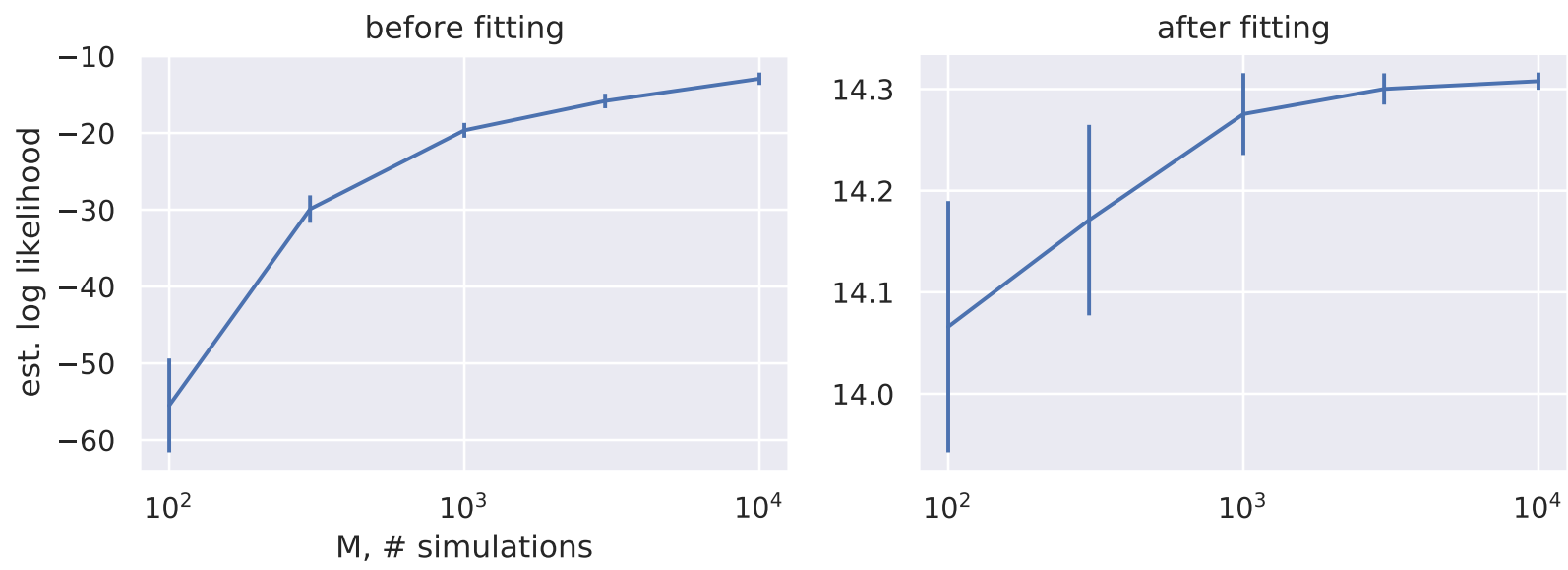

Fig. S1 Estimated log-likelihood of data for one participant given different numbers of simulations. We show the mean and standard deviations of the estimate based on 30 runs for each value of M.

1.3 Monte Carlo simulation

The likelihood in (S1) needs approximation. Given data from a single trial $\{\mathbf{x}_T, \boldsymbol{\varphi}\}$, we first sample from $p_{\text{R}}(\hat{\boldsymbol{\varphi}}|\mathbf{x}_T)$, alternating between (S4) and (S5) to obtain samples $\hat{\boldsymbol{\varphi}}^i$. Then we evaluate the samples on $p_{\text{A}}(\boldsymbol{\varphi}|\hat{\boldsymbol{\varphi}}^i)$. Since we will use many samples of $\hat{\boldsymbol{\varphi}}$, we also approximate the sum in (S13) with a single sample $\tilde{K} \sim p_{\text{A}}(\tilde{K}|\hat{\boldsymbol{\varphi}}^i)$. Putting things together, we estimate the log-likelihood of data (with a diminishing downward bias, see next section) as $\log p(\boldsymbol{\varphi}|\mathbf{x}_T) \approx$

$$\log\left(\frac{1}{M}\sum_{i=1}^{M} p_{\text{A}}(\boldsymbol{\varphi}; f(\hat{\boldsymbol{\varphi}}^i, \tilde{K}^i))\right), \tilde{K}^i \sim p_{\text{A}}(\tilde{K}^i|\hat{\boldsymbol{\varphi}}^i),\ \hat{\boldsymbol{\varphi}}^i \sim p_{\text{R}}(\hat{\boldsymbol{\varphi}}^i|\mathbf{x}_T^i),\ \tilde{\mathbf{x}}_T^i \sim p_{\text{n}}(\tilde{\mathbf{x}}_T^i|\mathbf{x}_T). \tag{S14}$$

We can alternatively view the sample $\hat{\boldsymbol{\varphi}}^i$ as the result of a simulation of the participant's mental model, which relies on a single particle of assignments sequence $\mathbf{z}_T$. This is then used to parametrize the aleatoric component, producing a valid likelihood function to be evaluated by data. As such, we will refer to drawing a sample $\hat{\boldsymbol{\varphi}}^i$ as a simulation. Under this view, we can use any other simulation-based procedure in place of p_{R} as our mental model for the participant's behavior in structured density estimation. We will introduce the batch-based model later.

1.4 Fitting algorithm

For each model, we fit all its parameters described in Table S1. We estimate the log-likelihood for numerical stability. However, the finite sample estimator above is biased. This is due to Jensen's inequality. Suppose each simulation provides a log-likelihood estimate of ℓ_i conditioned on some latent variables (such as $\mathbf{z}_T^i$ and $\tilde{K}^i$). Then the estimated marginal log-likelihood is

$$\mathbb{E}\left[\log\left(\frac{1}{M}\sum_{i=1}^{M} e^{\ell_i}\right)\right] \le \log\left(\mathbb{E}\left[\frac{1}{M}\sum_{i=1}^{M} e^{\ell_i}\right]\right) = \log(\mathbb{E}[e^{\ell_i}]), \tag{S15}$$

which is the marginal log-likelihood. Fortunately, this bias is downwards, meaning that the MC estimate provides a lower bound on the true log-likelihood; also, this bias diminishes as M increases (the estimator is consistent). This means we must

use a large M during training and evaluation. We show in Figure S1 the dependence of the estimated log-likelihood for different numbers of simulations, using data from a randomly picked participant in Experiment 3. When the model is untrained (left panel), the bias does not completely go away even at $M = 10\ 000$, but the variance is much smaller than using fewer M's. When the model is well-trained, both the bias and variance of the estimated log-likelihood are substantially reduced (right panel). Thus, the likelihood estimates become more reliable as the model fits the data better.

Thanks to the small estimation variance, we are able to fit the parameters using a gradient-free optimization routine Nelder-Mead implemented in the NLOpt package [1]. During training, we estimate the log-likelihood using $M = 10\ 000$ parallel simulations on NVIDIA GTX 1080 and A100 GPUs. The latter GPU provides a likelihood estimate within 3 seconds for Experiment 2, and 5 seconds for Experiments 1 and 3. The Nelder-Mead routine typically converges to a 0.001 relative precision on parameters within 300 iterations.

We restart Nelder-Mead 10 times with parameters found from the previous run, as this avoids premature convergence of Nelder-Mead. To further avoid local optima, we repeat this whole procedure (with 10 restarts) 10 times with different random seeds. We can then check if our algorithm has found a good solution by taking the maximum likelihood found across the 10 repeats. If this solution is reliable, then we should observe that this maximum is stable across the 10 restarts. We then take the best solution among the 10 random seeds at the last repeat as the fitted parameters.

1.5 Model simulation

To illustrate that the fitted DEE model captures the observed behavioral effects, we simulated model behavior using parameters fitted individually to each participant. We then applied the same visualization procedure to both the empirical and simulated datasets. For figures displaying correlations between reported cluster properties (Figures 3G, S2E, S3FG, and S4C), we excluded cluster reports whose means or widths exceeded five standard deviations from the overall distribution of reported cluster means or widths.

1.6 Alternative models

1.6.1 Batch-based variational GMM

We compared DEE against a non-sequential learning model. Conditioned on each possible model size $K \in \{1, \ldots, K_{\max}\}$, we placed a Dirichlet prior with uniform concentration α on cluster weights, and a normal-inverse-χ^2 distribution on each cluster, as in the DEE model. We then ran the variational Gaussian mixture algorithm, using our implementation adapted from the scikit-learn package [6]. Each simulation consists of $K_{\max}$ mixture models for $K \in \{1, \ldots, K_{\max}\}$ clusters. To decide which of the $K_{\max}$ models to report from each simulation, we first place a Poisson prior truncated

[1] https://github.com/stevengj/nlopt

to $K_{\max}$ on K,

$$p_{\mathrm{B}}(K) \propto \begin{cases} \mathrm{Poisson}(K; \bar{K}), & 1 \leq K \leq K_{\max}; \\ 0, & \text{otherwise.} \end{cases} \tag{S16}$$

Using categorical distributions for this prior did not lower the AIC, likely due to higher penalties from more free parameters. Out of the $K_{\max}$ fitted GMMs, we chose the one with the highest log-likelihood on the observed data subtracted by an AIC-like penalty

$$\log p(\mathbf{x}|\hat{\boldsymbol{\varphi}}, K) + \log p(K) - \kappa K, \tag{S17}$$

where κ is a parameter. The predicted model parameters are then sent to the aleatoric component, from which we can obtain an estimated log-likelihood of the reported cluster properties $p(\boldsymbol{\varphi}|K)$. To approximate the log likelihood, we ran 500 simulations, with each one containing $K_{\max}$ variational GMM fittings, which we found to be sufficient for the log-likelihood to stabilize.

We fit all parameters to each participant's data using the exact same procedure of DEE fitting, after augmenting the batch-based model with the same aleatoric component used in the DEE model. The free parameters optimized are the visual noise standard deviation σ_n, the concentration parameter α, Poisson rate $\bar{K}$, the model complexity weight κ, and the parameters of the cluster prior and the aleatoric component, as in the DEE model.

1.6.2 Ablations on the DEE model

We show that the two simultaneous modifications in the DEE from the CRP baseline, namely the **Economic** decay in expansion rate α_t and **Distorted** cluster count $n_{t,k}$, produce reliable improvements over the CRP. Applying one of these two modifications, or applying other modifications from the CRP baseline, produces improvements that were either unreliable or insignificant compared to the CRP model. We list the modifications on top of the CRP baseline below:

- **Economic Only**: adding a model-size dependent decay rate r, as in (S2).
- **Distorted Only**: adding an exponential transformation to the size of the clusters, as in (S2).
- **Merge Cluster**: when $\tilde{K}^i < \hat{K}^i$", instead of removing a cluster, the function $f(\hat{\varphi}^i, \tilde{K}^i)$ combines the smallest cluster (in terms of the cluster weight) with its nearest neighbor (in terms of the mean) to form a new cluster. Cluster properties of the new component are computed by moment matching.
- **Local MAP**: instead of sampling the cluster assignment according to (S5), take the cluster with largest posterior probability. This is the local MAP procedure described in [1].
- **Constant Variance**: instead of updating the variance of each cluster according to (S4), the cluster variance is fixed to the trainable parameter σ_0^2.
- **Fixed Mean**: instead of updating the mean of each cluster according to (S4), the mean is fixed at the first observation assigned to the cluster. This checks if

Table S1 Table of all model parameters.

Parameter name	Symbol	DEE component	Equation	Log-space?	Notes
Expansion rate	α_0	rational	(S2)	Y	
Decay rate	r	rational	(S2)	Y	optimized in economical, fixed to 0 in CRP
Cluster count distortion	β	rational	(S2)	Y	optimized in economical, fixed to 1 in CRP
Prior mean	μ_0	rational	(S3)	-	fixed to zero, not fitted
Pseudocount of prior mean	λ_0	rational	(S3)	Y	
Prior standard deviation	σ_0	rational	(S3)	Y	
Pseudocount of prior variance	a_0	rational	(S3)	Y	
Lapse on cluster count	ϵ	aleatoric	(S7)	Y	
Confidence in reporting cluster weight	c	aleatoric	(S9), (S10)	Y	
Gaussian noise sd in reporting cluster mean	σ_m	aleatoric	(S9), (S11)	Y	
Gaussian noise sd in reporting cluster log sd	σ_v	aleatoric	(S9), (S12)	Y	
Factor to shrink cluster width after splitting	s_σ	aleatoric	(S8)	Y	
Factor to shift cluster centers after splitting	b_σ	aleatoric	(S8)	Y	
Visual noise variance	σ_n	-	(S14)	Y	
Truncated Poisson prior mean	$\overline{K}$	batch rational	(S16)	Y	only in the variational GMM
Weight on model size penalty	κ	batch rational	(S17)	Y	only in the variational GMM
Cluster weight prior	-	batch rational	-	Y	only in the variational GMM

participants are able to update the mean or simply remember the first observations assigned to the clusters.

- **Fixed Mean Confidence**: instead of fitting λ_0 as a parameter, we fix it at $\lambda_0 = 0.01$.
- **No Visual Noise**: do not add Gaussian noise to the observations.
- **No Distribution Prior**: when computing the per-step assignment posterior in (S5), the likelihood is a Student's t-distribution obtained from having a conjugate prior over the Gaussian distribution parameters. Instead of computing this likelihood using the t-distribution, this modification computes this likelihood by a Gaussian with mean $\mu_{t,k}$ and variance $\sigma^2_{t,k}$.
- **No Counting Prior**: in (S2), instead of using the $n_{t,k}$ maintained for each cluster, use the average cluster size.

Model parameters are in Table S1. The results of fitting these variants, together with the DEE and the CRP-based models, on data from Experiments 1-3 are shown in Figure S6.

1.7 Modeling of recognition bias in Experiments 4–7

Three models were developed to account for participants' recognition bias. In each trial, after observing a sample sequence $\mathbf{x}_T$ of length T, n options were presented ($n = 4$ in Experiments 4, 5, and 6; $n = 2$ in Experiment 7). The decision variable for the i-th option is defined as:

$$d_i = \log P(\mathbf{x}_T \mid \text{option}_i) + w \cdot b(\mathbf{x}_T, \text{option}_i), \tag{S18}$$

where $\log P(\mathbf{x}_T \mid \text{option}_i)$ represents the likelihood of the samples under that option, capturing the strength of evidence for the option. The term $b(\mathbf{x}_T, \text{option}_i)$ indicates the recognition bias associated with the i-th option, while w is a parameter that scales the influence of this bias.

The probability of selecting each option is determined by a softmax function with a lapse rate γ, which accounts for random choices due to inattention:

$$P(\text{choose option}_i) = (1-\gamma)\frac{e^{d_i/\tau}}{\sum_j^n e^{d_j/\tau}} + \frac{\gamma}{n}. \tag{S19}$$

- **MLE**: The Maximum Likelihood Estimation (MLE) model assumes no recognition bias ($w = 0$) and bases decisions solely on the evidence term $\log P(\mathbf{x}_T \mid \text{option}_i)$. This model includes two free parameters: τ, which governs the sensitivity to the decision variable, and γ, the lapse rate.
- **DEE**: The DEE model incorporates a recognition bias that favors options with a specific number of clusters, determined by the number of observed samples T. The bias term is defined as:

$$b_{\text{DEE}}(\mathbf{x}_T, \text{option}_i) = \left|K_{\text{option}_i} - K_{\text{DEE-preference}}(T)\right|, \tag{S20}$$

  where K_{option_i} denotes the number of clusters in the i-th option, and $K_{\text{DEE-preference}}(T)$ represents the expected number of clusters under the DEE process given T.
  To evaluate whether the parameters fitted in the structured density estimation task generalize to the recognition task, we estimated $K_{\text{DEE-preference}}(T)$ by estimating the expected number of clusters $\mathbb{E}[K|\alpha, r, T]$ using Monte Carlo sampling of Equation S2, with (α, r) set to the participant-specific fitted values from Experiment 1. Specifically, we calculated:

$$K_{\text{DEE-preference}}(T) = \frac{1}{21}\sum_{i=1}^{21} E(K \mid \alpha_i, r_i, T), \tag{S21}$$

  where (α_i, r_i) are the fitted parameters for the i-th participant in Experiment 1. Note that the distortion parameter in Equation S2 does not affect the expected number of clusters and was therefore excluded when fitting the recognition choices. The DEE-based recognition model included three free parameters: τ, γ, and w.
- **CRP**: Similarly, we fitted a CRP model with a recognition bias term:

$$b_{\text{CRP}}(\mathbf{x}_T, \text{option}_i) = \left|K_{\text{option}_i} - K_{\text{CRP-preference}}(T)\right|, \tag{S22}$$

where $K_{\text{CRP-preference}}(T)$ was estimated using the parameters from the CRP models fitted in Experiment 1. The CRP-based recognition model also included three free parameters: τ, γ, and w.

2 Task instructions for the structured density estimation task

2.1 Experiments 1 and 2

Participants were instructed that their task was to predict the distribution of future samples by inferring the properties of latent clusters. Samples were described as "magic lava stones" emitted from "invisible volcanoes", and participants were told their predictions would help the Magic Council deploy energy collection fields optimally. Participants were told that their performance would be evaluated based on how well their drawn distribution graph predicted the rate of occurrence of future samples.

Participants were explicitly told they needed to infer three latent properties of each invisible volcano:

1. **Location**: where each volcano is positioned
2. **Emission range**: how widely stones spread around each volcano
3. **Activity level**: how frequently each volcano emits stones relative to others

These three properties correspond to the parameters of Gaussian mixture components (means μ_k, standard deviations σ_k, and weights w_k, respectively). All instructions were framed using intuitive, non-statistical language. The instructions used visual examples to show that stones from each volcano follow a bell-shaped distribution centered at that volcano's location, without introducing terms like "Gaussian", "normal distribution", or any mathematical formulation.

As participants adjusted their reports during the response phase, the overall marginal distribution (blue curve) was updated in real time on the screen. This real-time visualization allowed participants to immediately see how their reported cluster parameters combined to form the predicted distribution, enabling them to iteratively adjust their estimates to better match their internal representation of the distribution.

2.2 Experiment 3

Experiment 3 used the same cover story as Experiments 1 and 2, and also mentioned location, emission range, and activity level as the three key properties of each volcano. To make the experiment purely numeric, the instructions did not present the bell-shaped curve visually, but did mention that magic lava stones are more likely to land close to the center location of each volcano.

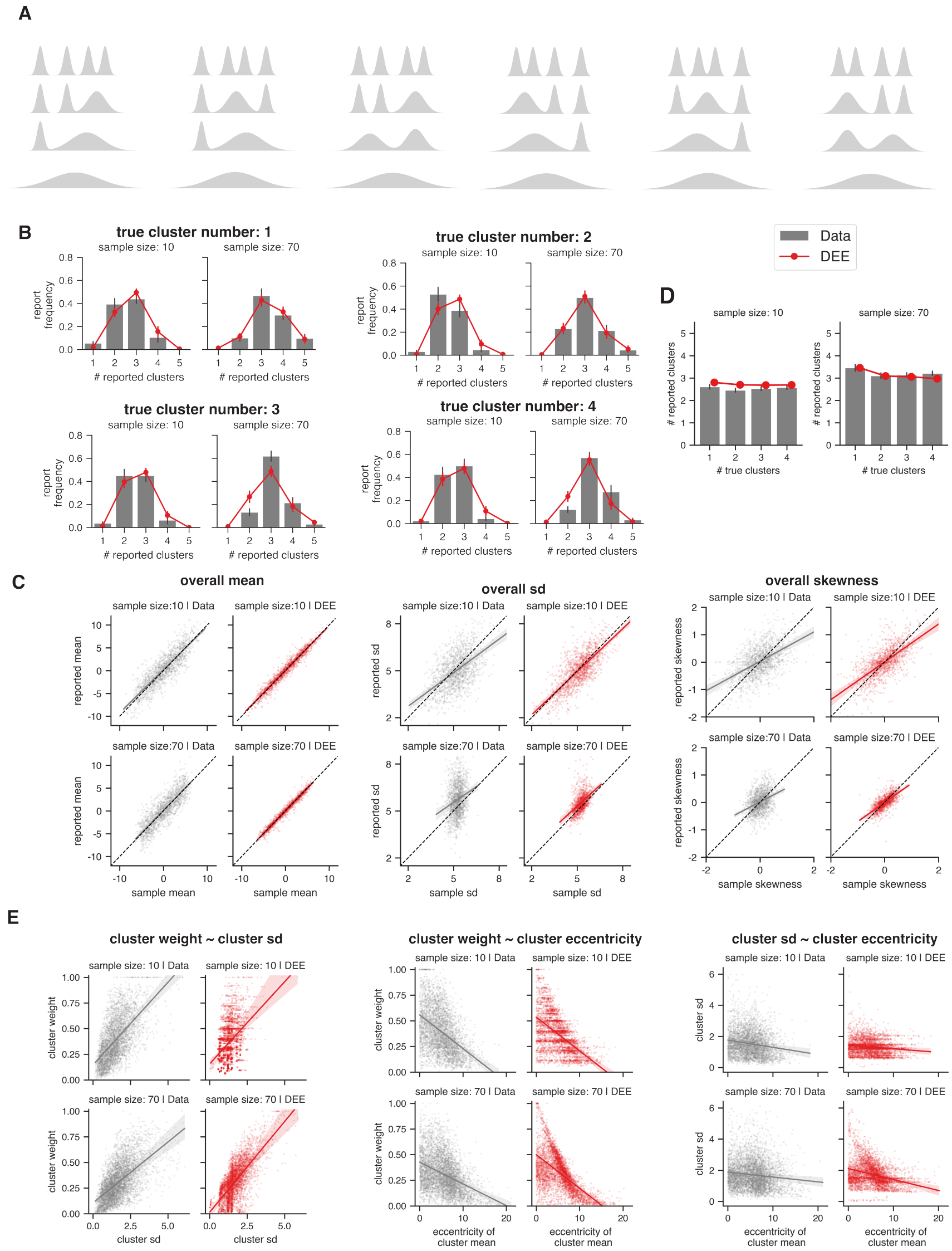

Fig. S2 Experiment 1 Full Results. **A**: The set of true distributions used in Experiments 1, 4, 5, 6, and 7. The true number of clusters was equally likely to be 1, 2, 3, or 4. All distributions were generated as described in Figure S5. Columns four to six are horizontally flipped versions of columns one to three, ensuring horizontal symmetry in the distribution set. **B-E**: Comparison between the empirical data and the simulated behavior of the fitted DEE model. **B**: Relative frequency of reported cluster numbers across different true cluster number conditions. **C**: Correlation between sample moments and reported moments. **D**: Change in reported cluster numbers as a function of the number of observed samples. **E**: Correlation between the reported properties of each cluster.

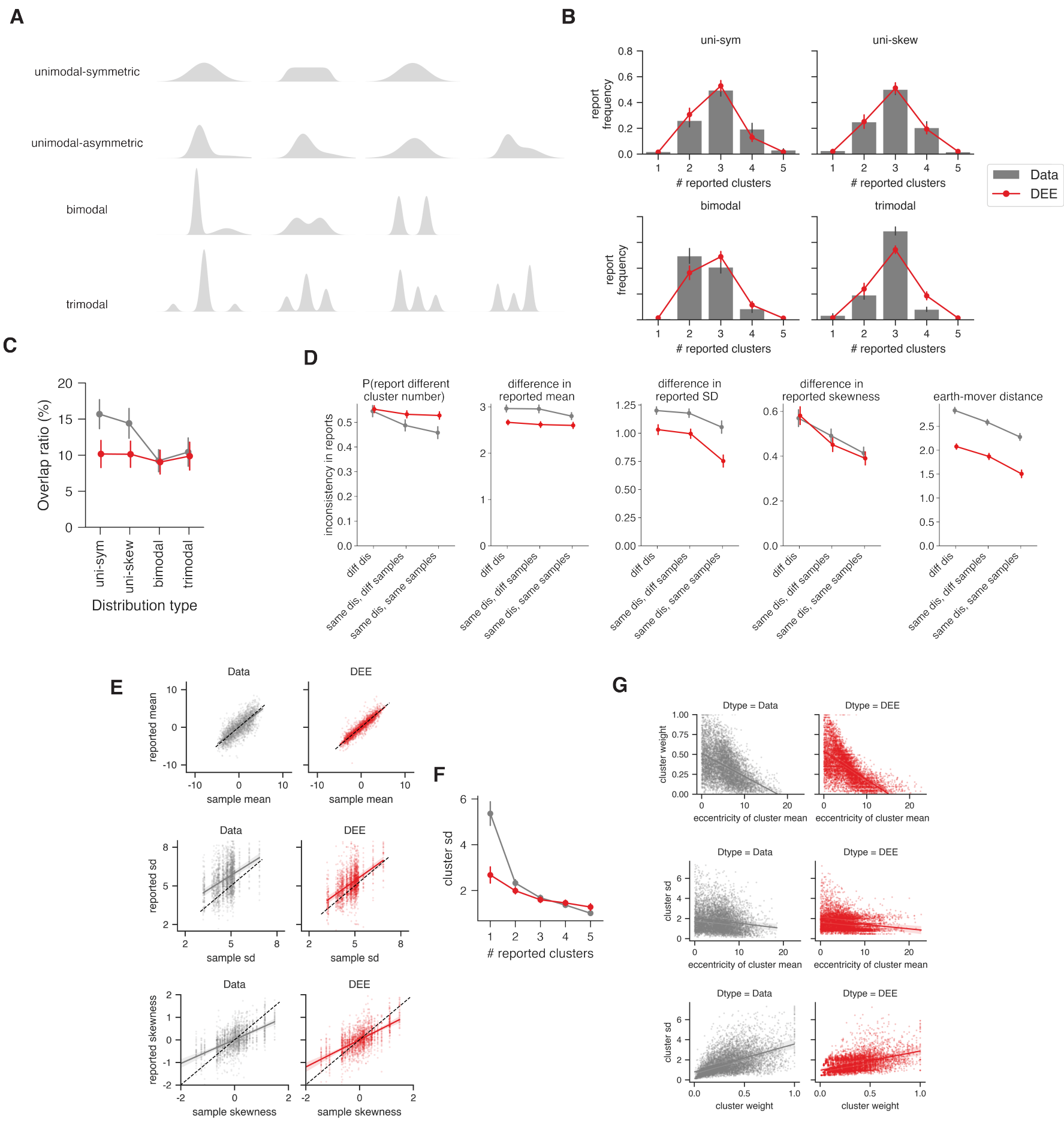

Fig. S3 Experiment 2 Full Results. **A**: The set of true distributions used in Experiment 2. The unimodal-symmetric subset includes (from left to right) a Gaussian distribution, a uniform-like Gaussian mixture, and a mixture distribution with two Gaussian clusters [from 7]. The unimodal-asymmetric subset comprises the first three distributions generated using the function family from [8], and the rightmost distribution sourced from [7]. The bimodal subset contains distributions from [9], [7], and [10] (left to right). The trimodal subset includes one distribution from [10] (leftmost) and three distributions from [11]. **B-G**: Comparison of empirical data with simulated behavior of the fitted DEE model. **B**: Relative frequency of reported cluster numbers across different true distribution conditions. **C**: Overlap ratio between adjacent clusters. **D**: Inconsistency in participants' reports across three comparison conditions: (1) different sample sequences from different distributions ("diff dis"), (2) different sample sequences from the same distribution ("same dis, diff samples"), and (3) identical sample sequences ("same dis, same samples"). Inconsistency is measured by: probability of reporting different cluster numbers, absolute difference in reported moments (mean, SD, skewness), and earth-mover distance. For earth-mover distance, reported densities are centered at the mean of the true generative distribution (as in Figure 1G) to isolate shape differences beyond location shifts. **E**: Correlation between sample moments and reported moments. **F**: Correlation between cluster width and the total number of reported clusters per trial. **G**: Correlation between the reported properties of each cluster.

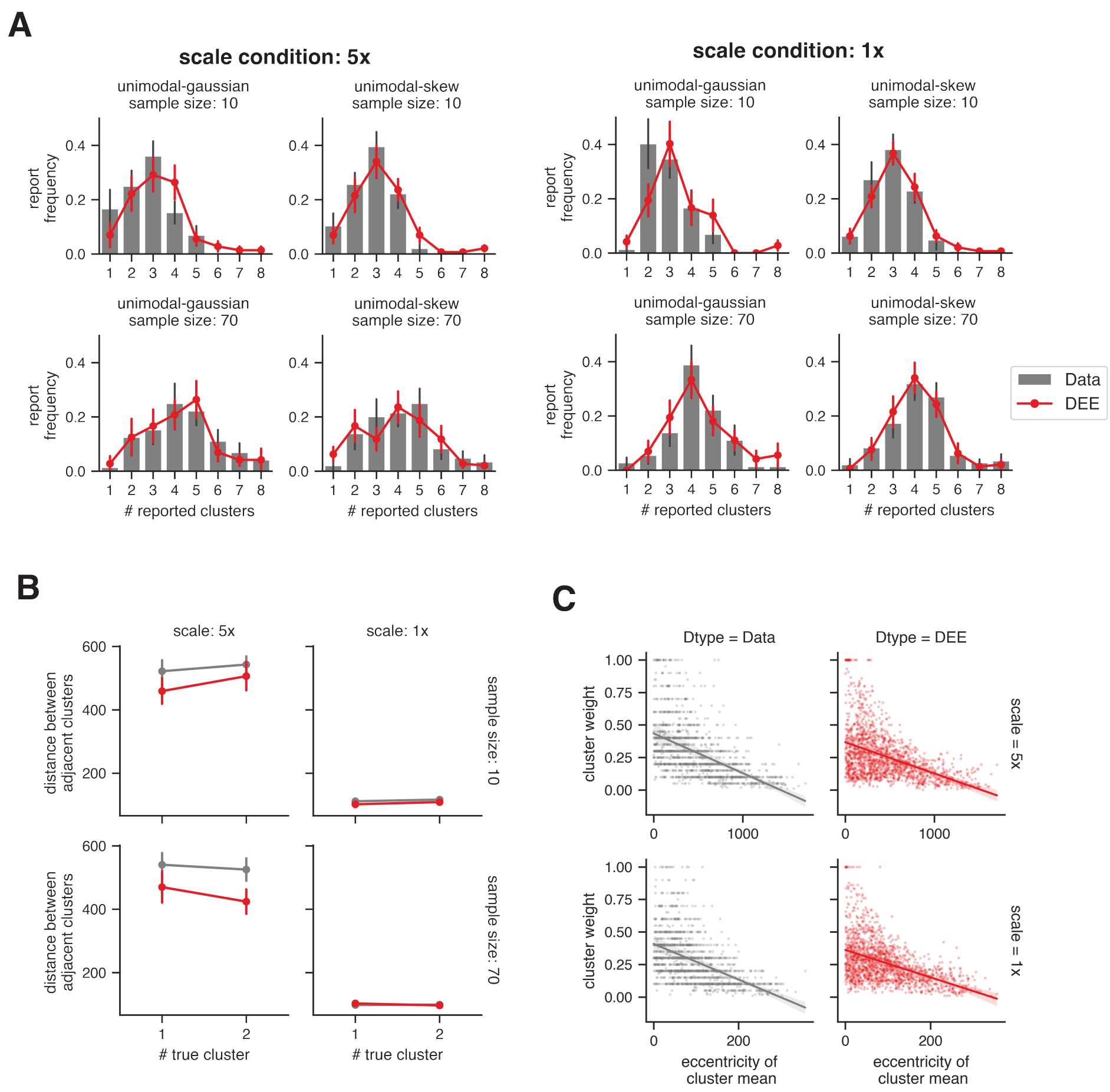

Fig. S4 Experiment 3 Full Results. **A**: Relative frequency of reported cluster numbers across different experiment conditions. **B**: The distance between adjacent clusters. **C**: Correlation between the reported properties of each cluster.

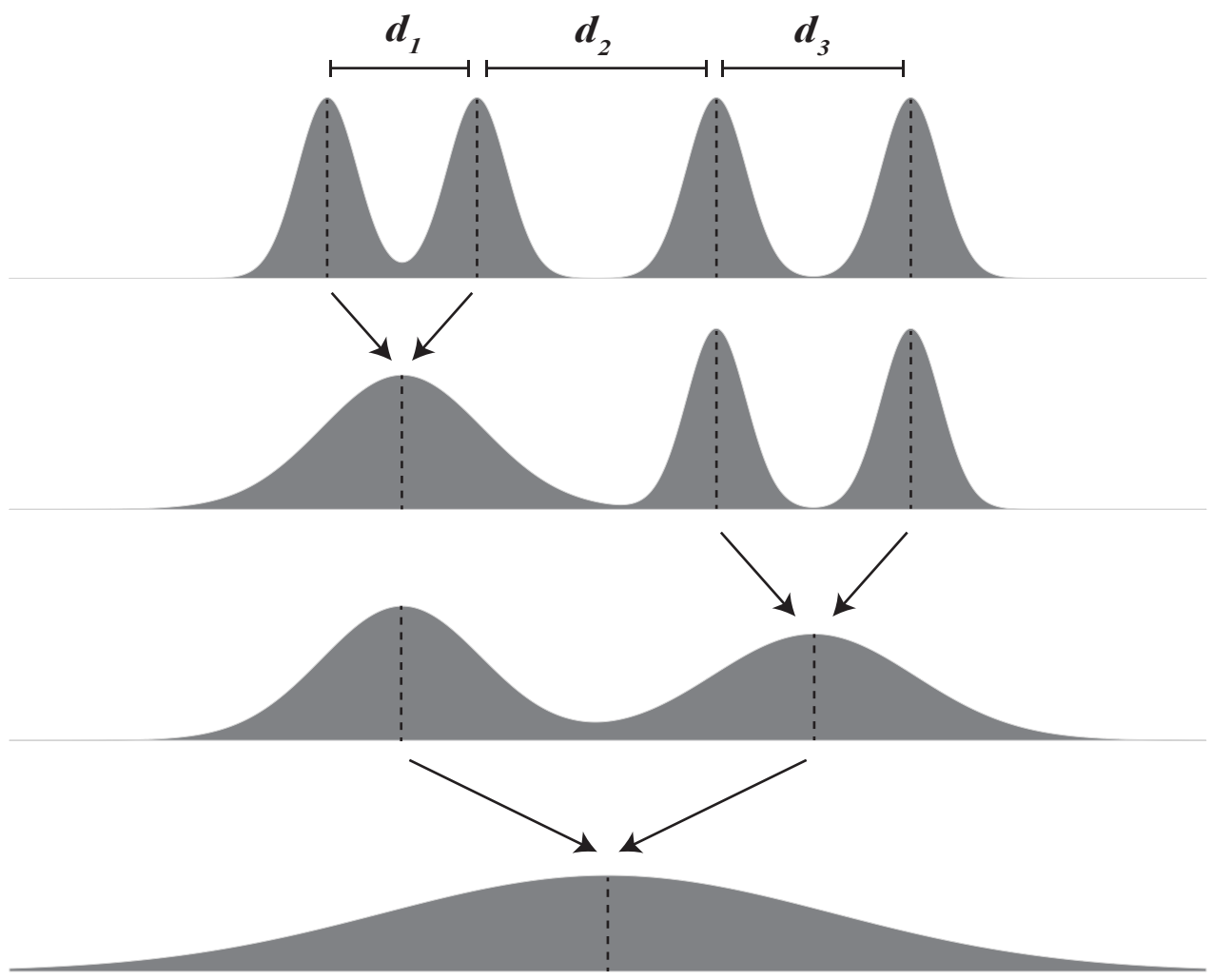

Fig. S5 The set of true distributions was constructed following the steps below. First, we created 6 four-cluster Gaussian mixture distributions (top row in Figure S2A) in which each component had the same SD of 1 a.u. and equal weight. The three center-to-center distances between their adjacent Gaussian components, denoted $[d_1, d_2, d_3]$ (from left to right), were chosen from the set of all permutations of $\{5, 6.5, 8\}$ a.u.. Second, each of the 6 four-cluster Gaussian mixtures went through "merging steps", inspired by the proposal step in the dynamic clustering algorithms (e.g. Reverse-jump MCMC) in statistics [12]. In each merging step, we chose two adjacent Gaussian components to merge into one, with the post-merger new component having the same zeroth, first and second moments as the combination of the two pre-merger components. The to-be-merged adjacent components were chosen in such a way that the post-merger Gaussian mixtures minimize KL-divergence $\text{KL}(p_{\text{pre}}||p_{\text{post}})$. By applying the merging step iteratively, the original four-cluster Gaussian mixtures were transformed into three-cluster, two-cluster, and finally one-cluster mixtures.The method description is adapted from [13] with permission from the copyright holder.

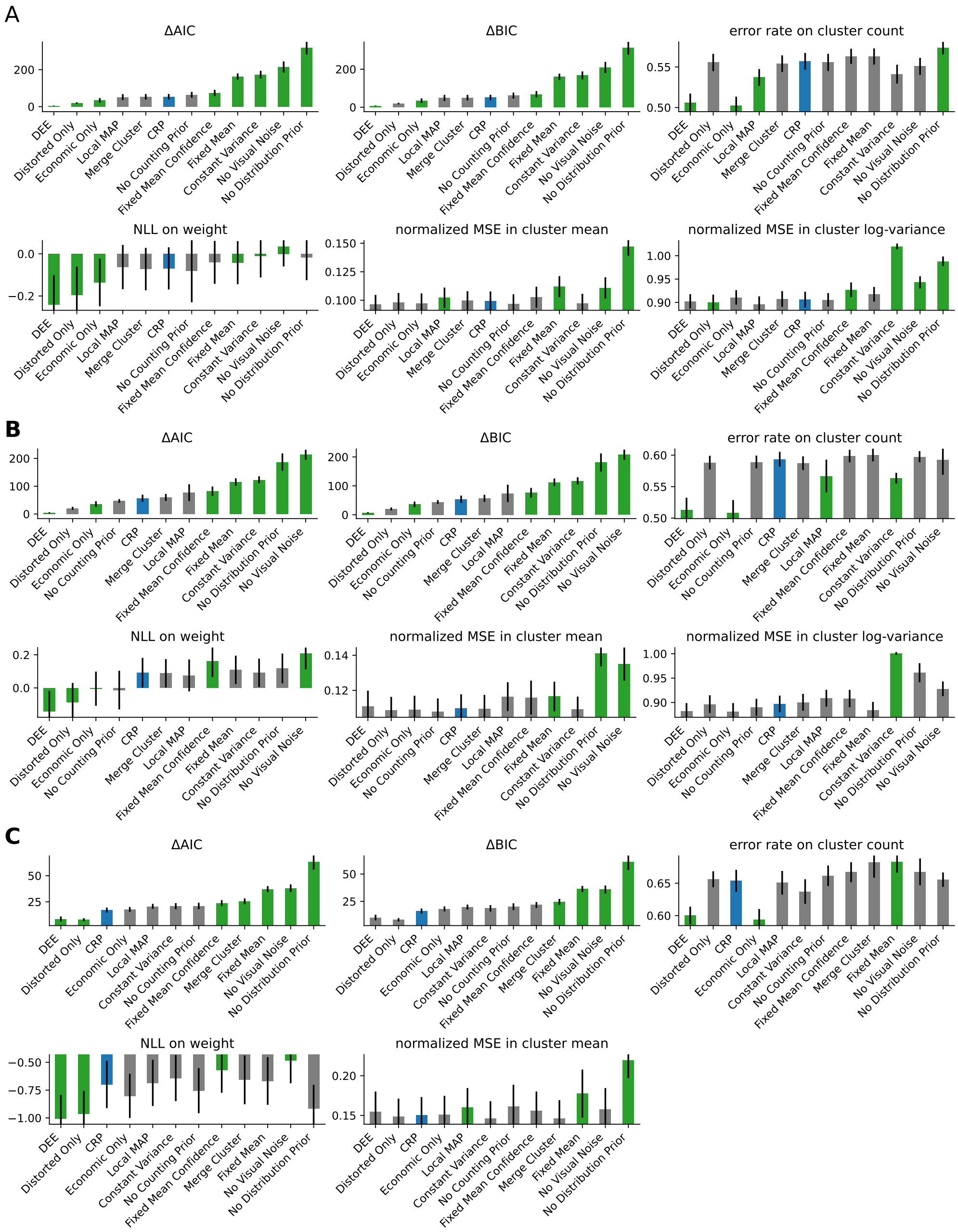

Fig. S6 ΔAICs and prediction errors for all model variants for Experiment 1(**A**), Experiment 2(**B**) and Experiment 3(**C**). In all panels, CRP is shown in blue. Green bars indicate models that differ significantly from CRP on that metric (Wilcoxon signed-rank test, $p < 0.05$), while grey bars indicate no significant difference.

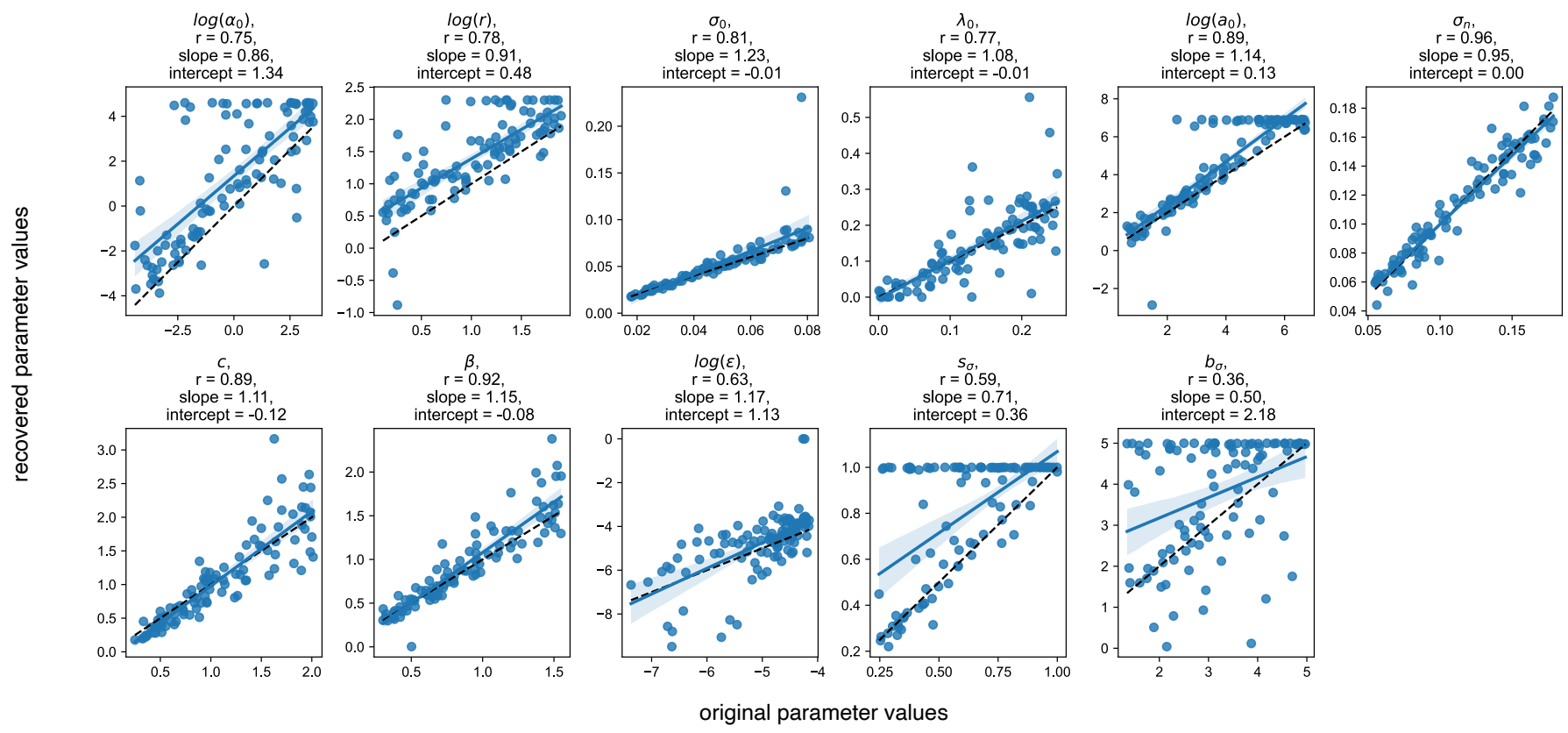

Fig. S7 Parameter recovery. We simulated responses from the DEE model using 100 randomly sampled parameter pairs, with trial settings identical to those in Experiment 2. The parameters were drawn uniformly from the range defined by the minimum and maximum values of the fitted parameters in Experiment 2. The noise levels in the final report of the cluster mean and standard deviation (Equations (S11) and (S12)) were set to the median of the fitted σ_v and σ_m. We then refitted the DEE model to each of the 100 simulated datasets and computed the correlation between the true (generative) and recovered (fitted) parameters. The mean correlation between simulated and recovered parameters was 0.76 ± 0.18 (SD across parameters), with mean linear slope of 0.98 ± 0.22 and mean intercept of 0.49 ± 0.75.

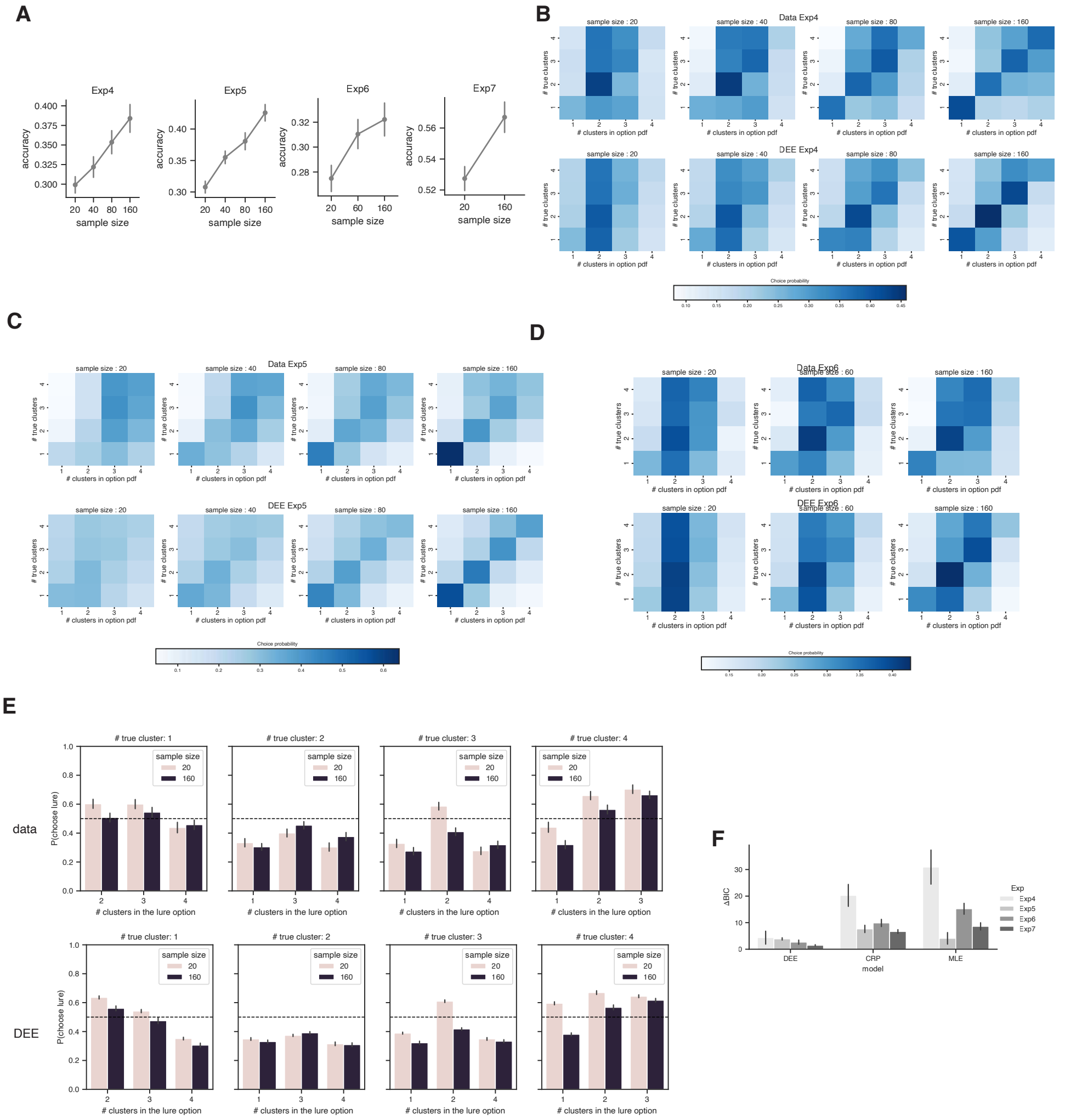

Fig. S8 Experiment 4,5,6,7 full results. **A**: Recognition accuracy increased with the number of observed samples, mirroring the findings in Figure 1B. **B-D**: Recognition confusion matrices comparing human data with simulated behavior of the fitted DEE model in Experiment 4 (**B**), Experiment 5 (**C**), Experiment 6 (**D**). **E**: The probability of choosing the lure option in Experiment 7. **F**: Model comparisons based on ΔBIC

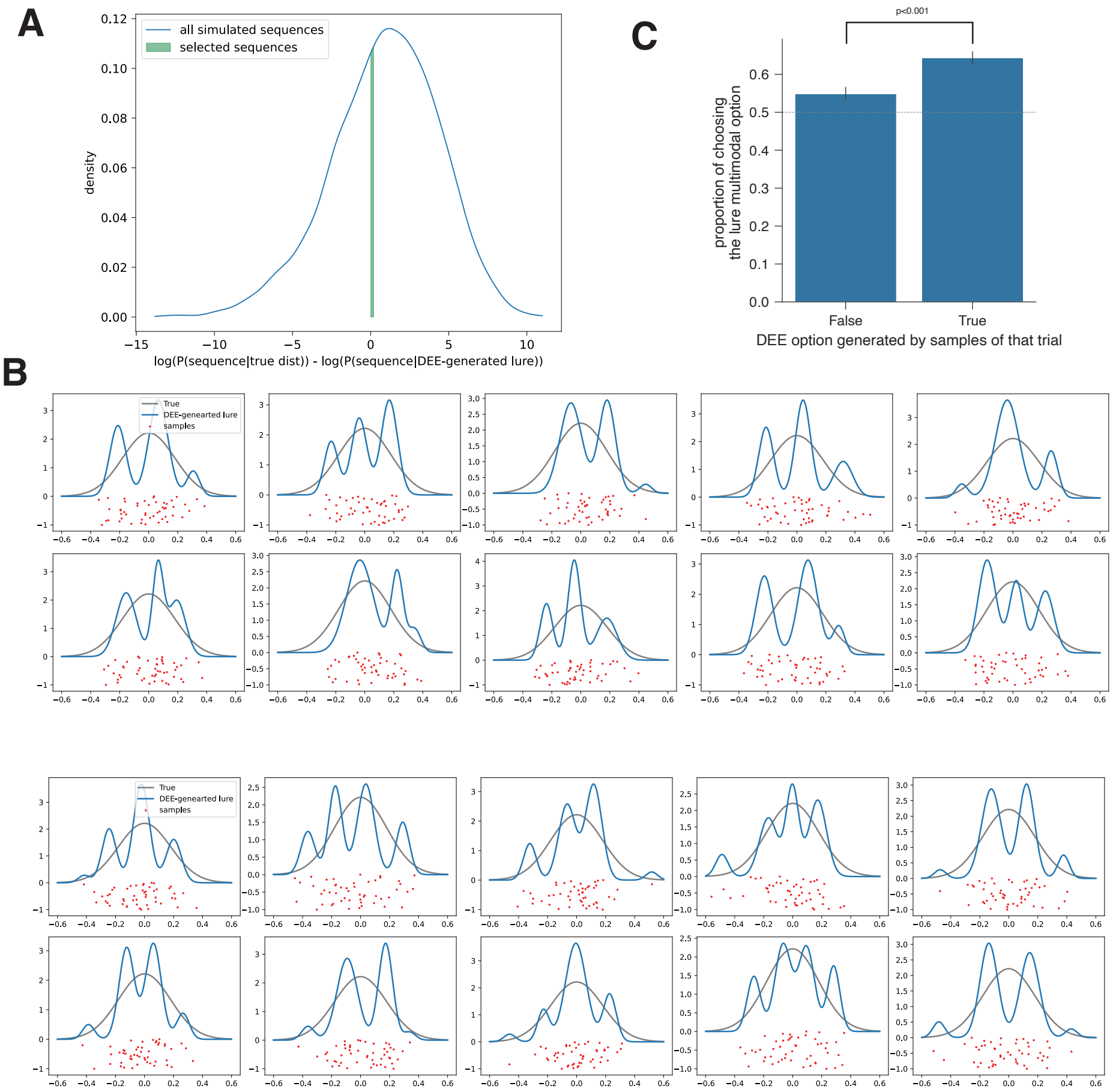

Fig. S9 Experiment 8. **A**: Log-likelihood difference of sample sequences under the true Gaussian distribution compared to the associated DEE-generated option. **B**: The 20 selected sample-option pairs used in the experiment. **C**: Participants showed a higher tendency to choose the multimodal lure in experimental trials compared to control trials.

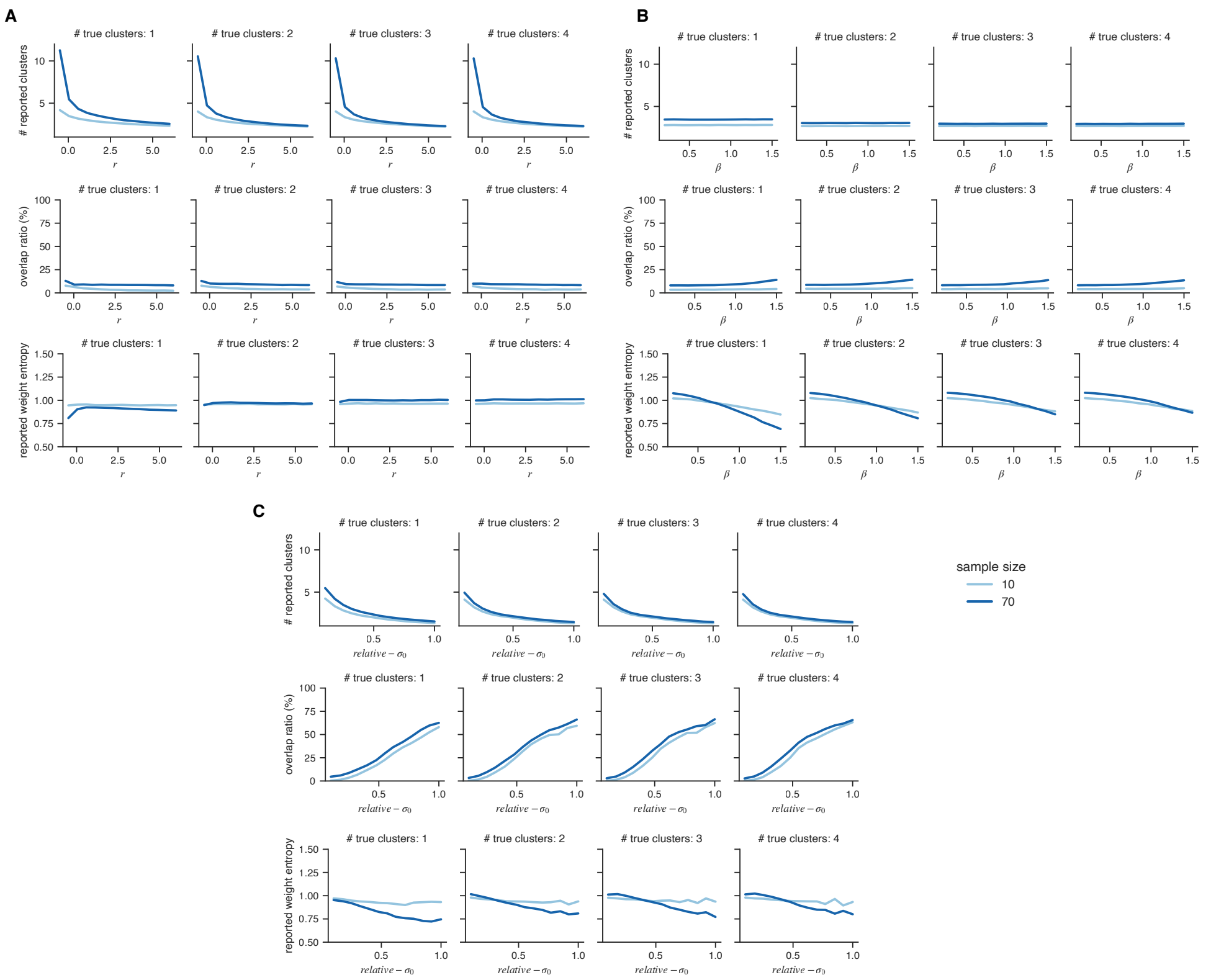

Fig. S10 Illustration of model behavior as function of key parameters. We simulated model-predicted behavior from Experiment 1 across a range of parameter values. For each trial, we computed the entropy of the reported cluster weights to quantify the certainty in the weight distribution within that trial. Higher entropy indicates more uniform weight distributions across clusters. Overweighting small probabilities and underweighting large probabilities will result in a higher weight entropy. In each panel, one parameter was varied while all other parameters were fixed at the median values fitted in Experiment 1. The overlap ratio between clusters and the reported weight entropy can be substantially influenced by the reported number of clusters. To ensure that the reported number of clusters does not confound the illustration of weight entropy and overlap ratio, we only included trials in which the model reported 3 clusters while plotting these two behavioral metrics. **A**: The economical expansion parameter r strongly modulates cluster number growth. As r increases, the number of reported clusters decreases for both sample sizes (10 and 70). At high r values, the difference in reported cluster number between the two sample sizes diminishes, indicating that r constrains sample-driven expansion due to memory limitations. In contrast, r has minimal influence on cluster overlap ratio and reported weight entropy. **B**: The probability distortion parameter β primarily affects reported weight entropy, with minimal influence on the number of reported clusters or their overlap ratio. **C**: The prior cluster width parameter σ_0 (relative-σ_0) influences all behavioral metrics. For consistency with Figure 3C, σ_0 is normalized by the standard deviation of the marginal distribution in Experiment 1. A relative-σ_0 of 1.0 indicates that the prior cluster width equals the marginal distribution's standard deviation. Smaller relative-σ_0 values lead to more reported clusters, lower overlap between clusters, and higher weight entropy.

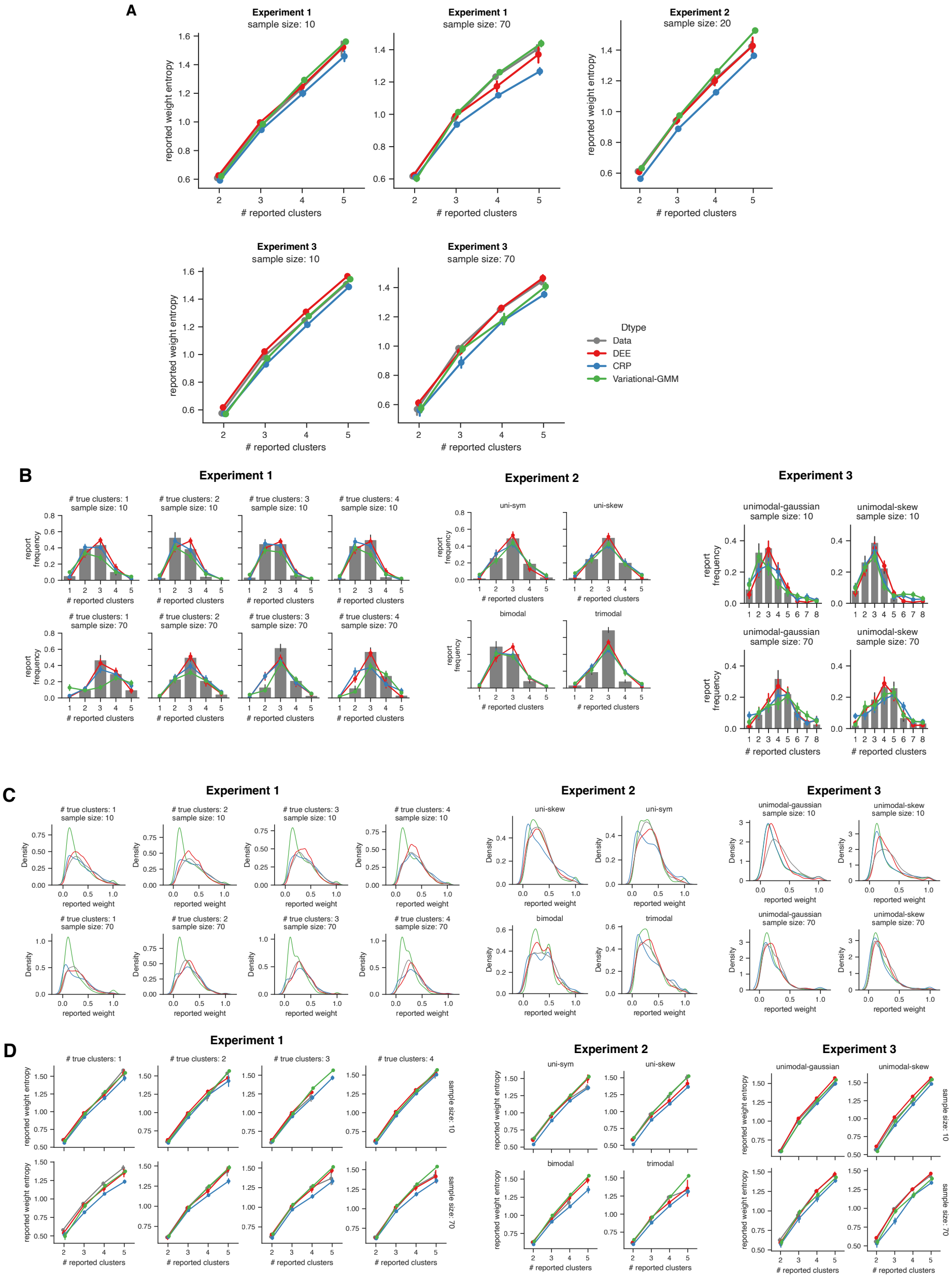

Fig. S11 Comparing DEE's behavior to alternative models. **A**: Reported weight entropy versus number of reported clusters. Weight entropy measures how evenly distributed cluster weights are (higher entropy = more equal weights). DEE and Variational-GMM match human patterns well, while CRP consistently underestimates entropy, indicating that probability distortion is necessary beyond basic CRP. **B**: Distribution of reported cluster numbers across experiments. **C**: Distribution of reported cluster weights across experiments. **D**: Weight entropy (from A) faceted by the type of true distribution.

Supplementary References

[1] Sanborn, A. N., Griffiths, T. L. & Navarro, D. J. Rational approximations to rational models: alternative algorithms for category learning. *Psychological Review* (2010).

[2] Courville, A. C. & Daw, N. The rat as particle filter. *Advances in neural information processing systems* (2007).

[3] Lloyd, K. & Leslie, D. S. Context-dependent decision-making: a simple bayesian model. *Journal of The Royal Society Interface* **10**, 20130069 (2013).

[4] Vul, E., Goodman, N., Griffiths, T. L. & Tenenbaum, J. B. One and done? optimal decisions from very few samples. *Cognitive science* **38**, 599–637 (2014).

[5] Miller, J. & Harrison, M. T. A simple example of dirichlet process mixture inconsistency for the number of components. *Advances in neural information processing systems* **26** (2013).

[6] Pedregosa, F. *et al.* Scikit-learn: Machine learning in Python. *Journal of Machine Learning Research* **12**, 2825–2830 (2011).

[7] Gershman, S. J. & Niv, Y. Perceptual estimation obeys occam's razor. *Frontiers in psychology* (2013).

[8] Körding, K. & Wolpert, D. M. The loss function of sensorimotor learning. *Proceedings of the National Academy of Sciences* (2004).

[9] Shin, Y. S. & Niv, Y. Biased evaluations emerge from inferring hidden causes. *Nature Human Behaviour* **5**, 1180–1189 (2021).

[10] Körding, K. & Wolpert, D. M. Probabilistic Inference in Human Sensorimotor Processing. *Advances in Neural Information Processing Systems* (2003).

[11] Sun, J., Li, J. & Zhang, H. Human representation of multimodal distributions as clusters of samples. *PLoS Computational Biology* (2019).

[12] Richardson, S. & Green, P. J. On bayesian analysis of mixtures with an unknown number of components (with discussion). *Journal of the Royal Statistical Society Series B: Statistical Methodology* **59**, 731–792 (1997).

[13] Teng, T., Li, K. & Zhang, H. Bounded rationality in structured density estimation. *Advances in Neural Information Processing Systems* **36**, 25211–25237 (2023).
