# Supplementary material for: Human learning of probability distributions is biased toward moderate structural complexity
Source: Nat Commun. 2026 Aug 8;17:9528. doi: 10.1038/s41467-026-76247-3 (PMC13547380; doi:10.1038/s41467-026-76247-3)
Supplement: Supplementary file 2 [file 41467_2026_76247_MOESM2_ESM.pdf]

nature portfolio

Corresponding author(s): Li Wenliang
Hang Zhang

Last updated by author(s): May 30, 2026

Reporting Summary

Nature Portfolio wishes to improve the reproducibility of the work that we publish. This form provides structure for consistency and transparency in reporting. For further information on Nature Portfolio policies, see our Editorial Policies and the Editorial Policy Checklist.

Statistics

For all statistical analyses, confirm that the following items are present in the figure legend, table legend, main text, or Methods section.

n/a	Confirmed	
☐	☒	The exact sample size (*n*) for each experimental group/condition, given as a discrete number and unit of measurement
☐	☒	A statement on whether measurements were taken from distinct samples or whether the same sample was measured repeatedly
☐	☒	The statistical test(s) used AND whether they are one- or two-sided<br>*Only common tests should be described solely by name; describe more complex techniques in the Methods section.*
☐	☒	A description of all covariates tested
☐	☒	A description of any assumptions or corrections, such as tests of normality and adjustment for multiple comparisons
☐	☒	A full description of the statistical parameters including central tendency (e.g. means) or other basic estimates (e.g. regression coefficient) AND variation (e.g. standard deviation) or associated estimates of uncertainty (e.g. confidence intervals)
☐	☒	For null hypothesis testing, the test statistic (e.g. *F*, *t*, *r*) with confidence intervals, effect sizes, degrees of freedom and *P* value noted<br>*Give P values as exact values whenever suitable.*
☒	☐	For Bayesian analysis, information on the choice of priors and Markov chain Monte Carlo settings
☒	☐	For hierarchical and complex designs, identification of the appropriate level for tests and full reporting of outcomes
☐	☒	Estimates of effect sizes (e.g. Cohen's *d*, Pearson's *r*), indicating how they were calculated

Our web collection on statistics for biologists contains articles on many of the points above.

Software and code

Policy information about availability of computer code

Data collection	We used Psychopy for human experiments. Experiments 1&2 used version 2021.1.2, Experiment 3 used version 2021.2.3, Experiments 4&5 used version 2020.2.4, Experiments 6&7 used version 2020.2.8, Experiment 8 used version 2022.2.5.
Data analysis	We used python (3.9), numpy (1.26.4), pandas (2.2.2), seaborn (0.13.2), and pytorch (1.9.1) for data analysis and modeling. All code is avalivable at https://osf.io/nk5z7/files.

For manuscripts utilizing custom algorithms or software that are central to the research but not yet described in published literature, software must be made available to editors and reviewers. We strongly encourage code deposition in a community repository (e.g. GitHub). See the Nature Portfolio guidelines for submitting code & software for further information.

Data

Policy information about availability of data

All manuscripts must include a data availability statement. This statement should provide the following information, where applicable:

- Accession codes, unique identifiers, or web links for publicly available datasets
- A description of any restrictions on data availability
- For clinical datasets or third party data, please ensure that the statement adheres to our policy

The data are available on the OSF platform (https://osf.io/nk5z7/files).

Research involving human participants, their data, or biological material

Policy information about studies with human participants or human data. See also policy information about sex, gender (identity/presentation), and sexual orientation and race, ethnicity and racism.

Reporting on sex and gender	Gender was self-reported by participants. Across all experiments, we recruited 547 participants (233 females, 299 males, 15 preferred not to disclose). In-person experiments (Experiments 1-5) included 142 participants (84 females, 58 males). Online experiments (Experiments 6-8) included 405 participants (149 females, 241 males, 15 preferred not to disclose).<br>The study was not designed to investigate gender differences, and we did not have an a priori hypothesis regarding gender effects. Therefore, no gender-based analyses were conducted.
Reporting on race, ethnicity, or other socially relevant groupings	For in-person experiments (Experiments 1-5), race and ethnicity data were not collected. For online experiments (Experiments 6-8), demographic information including race and ethnicity was automatically provided by the Prolific platform but was not used in study design or analyses. The study was not designed to investigate effects of race or ethnicity, and we did not have an a priori hypothesis regarding these factors on the cognitive processes examined.
Population characteristics	See above.
Recruitment	For in-person experiments (Experiments 1-5), we recruited participants through a university online forum (https://bbs.pku.edu.cn) at Peking University using convenience sampling (first-come, first-served). This approach resulted in a sample biased toward young university students (aged 18-28), which may limit generalizability to broader populations.<br>For online experiments (Experiments 6-8), we recruited participants through Prolific, which provided access to a much wider and more diverse participant pool spanning multiple age ranges (18-76 years), educational backgrounds, and geographic locations. This diversity in online recruitment helps establish the robustness of our findings across different demographic groups beyond the university student population.
Ethics oversight	The study was approved by the Institutional Review Board at the School of Psychological and Cognitive Sciences at Peking University.

Note that full information on the approval of the study protocol must also be provided in the manuscript.

Field-specific reporting

Please select the one below that is the best fit for your research. If you are not sure, read the appropriate sections before making your selection.

☐ Life sciences ☒ Behavioural & social sciences ☐ Ecological, evolutionary & environmental sciences

For a reference copy of the document with all sections, see nature.com/documents/nr-reporting-summary-flat.pdf

Behavioural & social sciences study design

All studies must disclose on these points even when the disclosure is negative.

Study description	Participants first observed a sequence of samples and were then required to report their belief about the underlying distribution. The data are quantitative.
Research sample	Experiments 1 to 5 were conducted on-site at Peking University, and participants were University students. Experiments 6 to 8 were conducted online, with participants recruited from the Prolific platform. Experiment 1 recruited 21 participants (11 females, aged 18–25). Experiment 2 recruited 21 participants (13 females, aged 18–25). Experiment 3 recruited 36 participants (21 females, aged 18–26), with 18 participants assigned to each scale condition. Experiment 4 recruited 31 participants (21 females, aged 18–28). Experiment 5 recruited 33 participants (18 females, aged 18–26). Experiment 6 recruited 80 participants (20 females, 11 participants did not disclose their gender, aged 20–56). Experiment 7 recruited 80 participants (27 females, 3 participants did not disclose their gender, aged 20–62). Experiment 8 recruited 245 participants (102 females, 1 participant did not disclose their gender, aged 18–76).
Sampling strategy	We used convenience sampling for all experiments. Each experiment recruited a different set of participants. For Experiments 1 to 7, we did not use statistical methods to predetermine sample size, but our sample size is similar to previous onsite experiments and online experiments. In Experiment 8, we first conducted a pilot experiment to estimate the effect size, based on which we estimated the sample size (reported in the preregistrationhttps://osf.io/4shqd/overview).
Data collection	In all in-person experiments, participants finished the experiments on a 21.5-inch iMac, with keyboard and mouse. In online experiments, participants finished experiments on their own desktop/laptop. The first author (TT) conducted all in-person experiments. No one was present besides TT and participants. TT was aware of the hypothesis when he conducted the experiment. The online experiments were self-paced and did not require the involvement of an experimenter to introduce or explain the task.
Timing	Experiments 1 to 7 were collected from September 2020 to January 2022. Experiment 8 was collected in March 2024.
Data exclusions	One participant was excluded from Experiment 2 due to their reported distribution mean being insensitive to the true mean, evidenced by a slope less than three standard deviations below the mean slope across participants. For Experiments 6 and 7, three comprehension check questions were included before the experiment began, and participants failing any of these were excluded. As a result, 24 participants were excluded in Experiment 6, and 13 participants were excluded in Experiment 7. Additionally, one

	participant in Experiment 6 was excluded due to the absence of a confidence report. Experiment 8 followed the preregistered exclusion criteria (https://osf.io/4shqd) and excluded 27 participants.
Non-participation	Data from 2 participants in Experiment 8 failed to reach the server due to technical errors.
Randomization	Only Experiment 3 used a between-subjects design, where participants were randomly allocated to scale conditions (1x vs. 5x), resulting in 18 participants per group.

Reporting for specific materials, systems and methods

We require information from authors about some types of materials, experimental systems and methods used in many studies. Here, indicate whether each material, system or method listed is relevant to your study. If you are not sure if a list item applies to your research, read the appropriate section before selecting a response.

Materials & experimental systems

n/a	Involved in the study
☒	☐ Antibodies
☒	☐ Eukaryotic cell lines
☒	☐ Palaeontology and archaeology
☒	☐ Animals and other organisms
☒	☐ Clinical data
☒	☐ Dual use research of concern
☒	☐ Plants

Methods

n/a	Involved in the study
☒	☐ ChIP-seq
☒	☐ Flow cytometry
☒	☐ MRI-based neuroimaging

Plants

Seed stocks	*Report on the source of all seed stocks or other plant material used. If applicable, state the seed stock centre and catalogue number. If plant specimens were collected from the field, describe the collection location, date and sampling procedures.*
Novel plant genotypes	*Describe the methods by which all novel plant genotypes were produced. This includes those generated by transgenic approaches, gene editing, chemical/radiation-based mutagenesis and hybridization. For transgenic lines, describe the transformation method, the number of independent lines analyzed and the generation upon which experiments were performed. For gene-edited lines, describe the editor used, the endogenous sequence targeted for editing, the targeting guide RNA sequence (if applicable) and how the editor was applied.*
Authentication	*Describe any authentication procedures for each seed stock used or novel genotype generated. Describe any experiments used to assess the effect of a mutation and, where applicable, how potential secondary effects (e.g. second site T-DNA insertions, mosiacism, off-target gene editing) were examined.*
