# Supplementary material for: Human learning of probability distributions is biased toward moderate structural complexity
Source: Nat Commun. 2026 Aug 8;17:9528. doi: 10.1038/s41467-026-76247-3 (PMC13547380; doi:10.1038/s41467-026-76247-3)
Supplement: Supplementary file 3 [file 41467_2026_76247_MOESM3_ESM.pdf]

nature portfolio

Peer Review File

Human learning of probability distributions is biased toward moderate structural complexity

Corresponding Author: Professor Hang Zhang

This file contains all reviewer reports in order by version, followed by all author rebuttals in order by version.

Version 0:

Reviewer comments:

Reviewer #1

(Remarks to the Author)
In this paper, Teng and colleagues show that humans exhibit a kind of "anti-Occam" phenomenon: they infer more latent clusters than exist in the data-generating process. The authors demonstrate this phenomenon repeatedly across a tour de force series of experiments. Computational modeling revealed that human behavior was consistent with the hypothesis that expansion of the hypothesis space (i.e., postulation of new clusters) is potentially unbounded but cognitively constrained.

Overall, I thought the main findings of the paper were really interesting, and the paper is overall well-written (though quite dense in some places). The modeling is done to a high standard. The paper has important implications for our understanding of inductive biases in human inference.

Major:

I find it a bit odd that in the Discussion the DEE model is framed in terms of conserving cognitive resources. I underestand that there is a resource parameter in the model, but it seems to me that the central empirical finding is that people are *less* economical than one would expect of a well-calibrated observer. They see more complex structure than is really there. I would appreciate if the authors could help resolve this tension in an intuitive way for the reader.

It's not obvious to me that online inference (e.g., with a single particle) should yield a bias towards over-complication of the learned structure (as claimed on p. 14). I would want to see a concrete demonstration of proof of this claim. I think actually that there are some simple counterexamples, though this depends on details of the setup.

Since the models play such an important role in the paper, I don't think they should be completely relegated to the supplement, especially since I found it hard to understand them based on the description in the Results. I suggest the authors include the key technical details of the models in the Methods section.

Minor:

The Methods section on Experiment 8 reports some experimental results. I think it would make sense to have all the results in the Results section, or possibly in the supplement.

Did the random-effects Bayesian model selection use the AIC, or an approximation of the log marginal likelihood?

p. 22: "handful parameters" -> "handful of parameters"

p. 23: I'm not sure what is meant by the statement "the approximate inference method using a single-particle no longer preserves exchangeability" since exchangeability here is a property of the prior, not the posterior (or its approximations).

p. 27: I think it's slightly misleading to say that the estimator in Eq. 14 is an approximation of the log-likelihood. As pointed out in the next section, Jensen's inequality implies that it's actually an approximation of a lower bound on the log-likelihood.

(Remarks on code availability)

Reviewer #2

(Remarks to the Author)
Review of "Illusory perception of latent probabilistic structure"
Reviewer: Michael Landy

This is quite a nice paper about how people build up a representation of a 1-d distribution, with the assumption of a Gaussian mixture model. The basic model, vastly simplified, is a forward process model matched to some of the experiments in which samples are shown to the observer one by one. The observer either puts the sample in an existing cluster (element of the mixture), updating its mean and SD, or creates a new cluster for it (with a default SD). Biases are introduced to reduce the likelihood of choosing to create a new cluster and to control cluster weights. The model never merges clusters, so that an overestimation of the number of clusters (when the true number is small) is kind of built in. The "economical" bias prevents models from getting too complex (and thus taxing structure memory in the observer). All in all the paper is impressive and convincing. I do have some comments, but this is very nice work.

Specifics, important and trivial, mostly by line number:

Figure 1E: certian -> certain

General question: If you showed all the samples simultaneously, I assume results would be different. So, this is really about the memory representation used when building a density estimate from sequential samples. If one sees all the samples at once, one can consider splitting a subset into separate clusters or merging them as a distinct, conscious decision. It's interesting that merging clusters is not needed for modeling the main task. But, the difference between your task and simple density estimation of a set of samples bears some discussion. Also, given that they had to report all cluster means, one by one, I wonder how much the results were do to the constraints on how participants made the report. Would it have been difference if, at any time, they could pick a cluster and delete it, move another, change its width, over and over until satisfied? Another thought: When presented with a sequence of random numbers, people see non-randomness that isn't there (e.g., sequences of identical digits or close real numbers seem surprising even though they are expected). Could clustering subsets be a bias in and of itself, in which case you'd also see too many clusters even with simultaneous presentation of the entire set, obviating the need for a sequential algorithm to explain it?

Figure 1C: The legend (or the figure) should say which row is which, rather than assuming the reader will notice the colors and look at the key above 1B.

Legend 1F: "reports were centralized relative to the mean of ...". I find that phrase obscure, so please clarify.

272-275: This is a pretty minimal definition of similarity between report and the twin trial only including number of clusters and nothing about the set of (mean,sd,weight) triples.

Figure 3A: Desnity -> Density, Schoastic -> Stochastic

Figure 3C (twice): gets richer -> get richer

400: unobserved to -> unobservable by

437: modulated by memory resource -> modulated by limited memory resources

444-445: I don't really buy the tasks for Expts. 4-8 as being a different requirement for the participant. Sure, they don't have to explicitly state the number of clusters. But, they do need to build up a representation and then compare it to options that have a clear number of clusters.

619: manifests -> manifests

721: "participants cluster property reports". First, it needs an apostrophe: participants' reports. But the phrase "cluster property report" is needlessly obscure.

Expt. 3: Was this a different set of participants from Expts. 1 and 2. Otherwise, they might have been prompted to think about the task visually despite the numeric stimuli. In fact, given the task, it seems likely that many might have used an internal visual number line to do this task. Did you ask?

974: Why are you using \Sigma instead of \sigma for a 1-d SD in the notation?

975: I assume \Delta^K is meant to mean the K-dimensional simplex (k-tuples that are positive and sum to one), but I'd bet many readers won't know that, so clarify, please.

1000: handful OF parameters

1004-1005 and elsewhere: I'd use the standard term "lapse" rather than "slack" throughout. Note that this "lapse" is a flat distribution over an infinite domain (i.e., improper).

Eq. 2 and 1028: \alpha_t is proportional to the probability of making a new cluster. Thus, that probability is reduced when r is large and positive, the opposite of what you say on line 1028.

1032: From here down, the text is pretty much for the cognoscenti and very dense. If you want this more widely read, you'll need to add some introductory material and some clarify. First, I don't remember what "exchangeable" means in this context.

1034: "single-particle" doesn't need a hyphen here. More important, the use of the word "particle" comes out of the blue. In effect you have a forward process model that could be run a bunch of times in parallel, like a particle filter, but you never say that out loud. So, put this in a context so the reader knows where you are going with it.

1042: I didn't re-educate myself on this. I thought the standard model was normal-inverse-gamma (for mean/variance). Maybe normal-inverse-\chi^2 is the standard thing if you use SD instead of variance (I don't know and didn't check). But again, this is not knowledge that your readers will know, so be more didactic as you lay things out.

1051: \bold{z}_t^i: what is "i"??? You never say and I couldn't figure it out from the context. I'm wondering if the "i" throughout is an index for possibly multiple particle runs. Maybe yes, maybe no. I'm lost at this point with your notation.

1054: Either "over Gaussian mixture parameters" or "over the Gaussian mixture parameter"

1067: I have no idea what "(unadjusted)" means here

1094-1095: \mu_{T,k} gets a superscript i in one instance and not in the other???

1100-1102: As I said above, this statement is the crux of your model and is hugely important, but is buried in math methods in the Supplement and never discussed explicitly in the main text.

1111: number of clusterS

1134/1138: I think you have the cases backward, case 1 is for K-tilde > K-hat (so that you have to merge) and case 2 is the opposite.

1262: I may have missed it, but the acronym "DEE" seems only to be spelled out in a figure legend and not in the main running text.

1286: Our -> Out

1314-1315: "the function f(\phi,\hat{K}) merge the smallest cluster" doesn't parse and I'm not sure what you meant to say here

Figure S2E right-hand plots: Isn't this negative correlation inevitable for two clusters? If they are to match the SD of the samples, then when they are further apart they have to reduce their SD to compensate. The result seems built-in.

Figure S&: You plot the correlation for a parameter recovery. But, for recovery you don't just want a high correlation, you want the scatterplot to be close to the identity line. Seems the wrong statistic to describe how close you achieved that. Why not include some scatterplots (with identity lines) of simulated vs. recovered?

1836* The probability of CHOOSING

Various figures: axis label should be "# of reported clusters"

(Remarks on code availability)
N/A

Reviewer #3

(Remarks to the Author)
Teng, Li, and Zhang examined people's internal representation of latent structure underlying sequentially observed data. Using a structured distribution report task, the authors have found that although people could report the probability density relatively well, they fail to identify the true underlying structure. The authors have developed a model termed distorted economical expansion process (DEE), to capture people's behavior in both the self-report task and a series of AFC tasks. The DEE model builds upon the Chinese restaurant process (CRP) with additional parameters as well as an aleatoric component to accounting for cognitive constraints and randomness in participants' behavior.

The research question is important and the task design is novel and suitable to probe people's internal representation. In principle, the finding that the same sorts of biases that impair decisions from description also lead to systematic biases in understanding the structure of the environment, would certainly be novel and of lasting importance to a broad audience. However, we believe that more analysis of both human and model behavior are needed to substantiate that claim – and that

more discussion is necessary to clarify and contextualize it.

Main comments:

One question we had in trying to understand this paper is whether the behavior metrics that are focused on reflect structural understanding, or a probabilistic decision strategy used to report it. More specifically, whether economic biases impact structural understanding – or whether economic biases impact a series of one-off decisions through which that understanding is communicated in this particular task framework. Participants were not able to "undo" a mixture component once it was specified. If you consider that the decision about where to place the first "cluster" might be both probabilistic and systematically biased, as we would expect from existing decision making literature, and that the subsequent decision would be made conditional on the first, couldn't you get many of the reported results even with perfect underlying knowledge of the structure? Would this idea extend to the series of 2AFC task results that were also reported? The manuscript does not currently grapple with the distinction, and to our view, it needs to in order to support the primary claim that the biases measured arise at the level of a structural understanding, rather than from the decisions based on it.

The key behavioral features in the human subject data – and how these features are affected by the parameters of interest in the DEE model – were not sufficiently clear to evaluate the strength of evidence for each individual economic bias contributing to behavior. The manuscript overall seems a bit vague on what the key behavioral pattern is in the structured distribution report task. In the abstract section, the authors made the claim that people typically overestimate the cluster size. Though this seems true when # of true cluster <= 2 with a small sample size (figure 1D), this is not the case when # of true cluster >=3. It also seems that the reported cluster number does not differ much when the underlying cluster number changes (Figure S2B). In discussion, the author wrote '[participants] struggled to identify the true generative structures even with extensive observations.' The overall behavioral claim needs to be more clear and quantified by specific conditions throughout the manuscript. Furthermore, the behavioral analyses focus on two experimentally manipulated factors (number of clusters, number of samples) and show that the former has no influence on reported number of clusters, while the latter does. While these findings are enough to falsify key predictions of a CRP, they are not particularly informative with regards to what subjects are actually doing, nor are they sufficient to explain why the particular biases in the DEE model might be necessary to explain the behavior. Presumably, there are some more systematic trends in human behavior that are captured by the models – for example that specific experimental mixture distributions tend to elicit more reported clusters – and perhaps these trends could shed light on why specific components of the model are necessary to capture these. Our view is that fully supporting the primary claim would require examining the factors that systematically influence structural reports and showing how key parameters of the model allow it to reproduce the influence of those factors on behavior.

Along the same lines, providing more details for other aspects of the behavioral data could be helpful. For example, what is the distribution of weights people put on different clusters? The authors have reported skewness but it only captures people's report on a trial aggregated level. Is the weight distribution different under different conditions (# of clusters, sample size)?

The major contribution of the DEE model, as far as we understand, is to incorporate cognitive constraints into the clustering process. The authors have done a comprehensive model comparison to show the superiority of DEE (Figure 3D and Figure S6) focusing on model fit metrics, but it is not clear how these model components change its behavior. How does behavior of the model change when parameters thought to reflect different cognitive constraints are turned up or down? Do specific constraints affect specific aspects of the measured behavior? And are these aspects of behavior clearly related to systematic biases in structural understanding? It would also be very helpful to see figures that overlay model prediction and human data for the other model candidates similar to Figure 3E-G, but for a wider range of behavioral metrics.

The computational model plays a pivotal role in supporting the primary claim made in this paper. There is no way to evaluate the evidence for the claim without understanding the model. Thus it is our view that the modeling methods (or at least the critical ones) should be included in the main paper rather than the supplementary information.

Other comments:

The primary novel claim of the paper, at least to our understanding, is that cognitive biases lead to systematic structural misunderstandings. Given this, it seems critical that the discussion contextualize the results in terms of the cognitive biases explored here and how they relate to the larger context of work in decision making on cognitive biases – for example, we were very surprised not to see any mention of Prospect theory in the discussion, despite one of the core components of the model being a subjective probability much like that incorporated by prospect theory.

Discussion should also provide broader context of other paradigms that have been used to measure how people conceptualize probability distributions, and how the current paradigm might differ from these. For example, DiBerardino 2024 provides a very rich way to characterize people's beliefs about probability (specifying a complete distribution), whereas Nassar 2010 provides a much more constrained one (just mean and variance). This context seems somewhat necessary for interpreting the broader claims of the paper, in particular, whether the biases observed are related to structural understanding, rather than how it is reported. In principle, the bias to see more clusters than truly exist would be consistent with the overly high "hazard rate" seen in Nassar 2010, since considering a new changepoint is essentially adding a new latent cause.

In Figure 3G, how is the eccentricity of cluster mean calculated? Is Eccentricity of cluster mean defined as distance from the mean of the full mixture distribution? If so, doesn't the weight have to be negatively correlated with this – since a higher

weight would also pull the mean toward the cluster. If this is a key qualitative effect that the model captures – it shouldn't be something that is tautological.

It is interesting to see the impact of sample size on human reports. However, it does not seem to be motivated enough in the introduction - why are we interested in people's reports under small and large sample sizes? Presumably this effect alone is enough to falsify the CRP, which is an exchangeable model? Also, can the DEE model reproduce the results in Figure 1D?

Experiment 2 results suggest that people are relatively inconsistent in reporting cluster numbers when facing the identical observation stream. It would be helpful to provide more discussions on this point. For example, can the DEE model reproduce this inconsistency? If not, does this suggest that there are other sources of randomness that the aleatoric process fails to account for?

For model comparison, is the CRP and/or variational-GMM model equipped with an aleatoric process as well? Otherwise, it does not seem to be a fair comparison.

Authors should report the instructions of experiments 1 and 2 more in detail - the instructions seem important in setting up the experiment and we are not sure how well people understand the action of reporting several gaussian distributions.

Figure 3D should specify what \delta AIC means in the caption.

Figure S6 should specify the meaning of different colors.

Figure S7 caption should be 'parameter recovery' instead of 'model recovery'.

(Remarks on code availability)

Reviewer #4

(Remarks to the Author)
I co-reviewed this manuscript with one of the reviewers who provided the listed reports. This is part of the Nature Communications initiative to facilitate training in peer review and to provide appropriate recognition for Early Career Researchers who co-review manuscripts.

(Remarks on code availability)

Version 1:

Reviewer comments:

Reviewer #1

(Remarks to the Author)
The authors have done a good job addressing my comments. I'm happy to recommend publication.

I don't want to make a big deal about this, but I don't think -AIC/2 is technically a proper approximation of the log model evidence. This is true for -BIC/2 asymptotically (see for example the derivation in the Bishop 2006 textbook), but as far as I know this doesn't hold for AIC, which is derived from different (non-Bayesian) principles. Maybe the correct thing here is just to say that the authors use this as a model score but don't commit to the Bayesian (marginal likelihood) interpretation.

A few small things:
- p. 10: a phrase ("this enables...") is repeated twice.
- p. 17: "there is not explicit, step-wise merging operation" -> "there is no explicit, step-wise merging operation"
- p. 24: "memory laod" -> "memory load"
- p. 24: "Previous work have established" -> "Previous work has established"
- p. 25: "estimated number of cluster" -> "estimated number of clusters"
- p. 25: "The parameters of the chosen model is taken" -> "The parameters of the chosen model are taken" ... "which is" -> "which are"

(Remarks on code availability)

Reviewer #2

(Remarks to the Author)
Re-review of "Illusory perception of latent probabilistic structures"
Reviewer: Michael Landy

I still like this paper and it's a bit more readable now, which is a good thing. I now fully understand the models, although I did still get a bit lost in some of the details in the Supplement. My comments are trivial. This thing is ready to go.

* 160: "see dashed lines": This is the only reference to the two contours in 1F. The legend should state what black vs. blue is.

* 174: "effect of true cluster number": From the figure, this seems to be driven entirely by the stimuli with a single cluster

* 349: "DEE" should be spelled out at first use (probably here), not at 353

* 435: As I said last time, I think, "r" is not the right statistic. You don't merely want the recovered parameter to be linear in the true parameter; you want them to be close to equal (slope 1, intercept 0).

* 754: not explicit -> no explicit

* 756: bit fit -> better fit?

* 1070: number of clusters -> number of items

* 1074: laod -> load

* 1115: cluster -> clusters

* 1117-1118: sample -> samples

* 1324: a the -> a

* Eq. S5: The probabilities of the two cases should be 1-\epsilon and \epsilon. There's no need to remove \hat{K}_i from the 2nd case. Leaving it in just corresponds to a slightly different value of \epsilon.

* 1487: creates a lapsed -> creates lapsed

(Remarks on code availability)

Reviewer #3

(Remarks to the Author)
We thank the authors for their responses to our comments. We especially appreciate figures S10 and S11, which have linked the parameters in the DEE to behavioral metrics and show that DEE does a better job than other model candidates to qualitatively capture people's behavior. Overall, we think the paper has been strengthened and clarified. However, despite this, we still have few remaining concerns with the interpretation of the results and its communication.

• In our initial review, we raised the concern that the observed bias may partly arise from the sequential structure of the task (i.e., reporting the cluster location and its properties in sequence). While the authors provide new evidence supporting that the effect generalizes across experimental conditions and variants, there are still some remaining questions regarding the origins of some of the primary effects. For example, the authors note that it was possible for participants to effectively eliminate a cluster by setting its weight to zero – however, the fact that participants never do so could be interpreted as evidence for them conditioning subsequent inference on this initial response, which would be a form of the sequential effects that we were concerned about. Basically, we worry that sequence based explanations exist for many of the individual results and assuming that these cannot be rejected directly through analytic approaches, they should be discussed in a more balanced fashion.

Beyond the sequential effects driven by the task design, it is also quite possible that individuals undergo a series of cognitive decisions, leading to sequential effects related to internal, rather than external, decisions processes. This applies to the 2AFC task, as participants may internally engage in a sequential evaluation before committing to a binary choice. We are not requesting that the authors directly resolve this issue, as doing so would likely require substantial and creative changes to the task design. However, we believe the newly added discussion paragraph should be more cautiously framed and more explicitly acknowledge that some of the observed effects may stem from (mental) sequential cluster reporting.

• It would still be valuable to mention prospect theory in the manuscript. Although the authors provide several reasons for why it may not apply, we see little downside to briefly citing and discussing it. Given the conceptual overlap, some readers (including ourselves) are likely to draw connections between the present work and prospect theory, and an explicit clarification of the similarities and differences would therefore be helpful.

• Our concern regarding inconsistency in human behavior has not been fully addressed. The authors note that "our model could reproduce some of the variability exhibited in human structure learning reasonably; residual mismatches remain." While we do not expect the current model to fully account for these inconsistencies, we believe this point warrants further discussion. For example, what specific residual mismatches remain? What mechanisms might plausibly give rise to the

observed inconsistencies in behavior?

(Remarks on code availability)

Reviewer #4

(Remarks to the Author)
I co-reviewed this manuscript with one of the reviewers who provided the listed reports. This is part of the Nature Communications initiative to facilitate training in peer review and to provide appropriate recognition for Early Career Researchers who co-review manuscripts.

(Remarks on code availability)

Version 2:

Reviewer comments:

Reviewer #2

(Remarks to the Author)
Fine, accept!

(Remarks on code availability)

Reviewer #3

(Remarks to the Author)
The authors have fully addressed our concerns.

(Remarks on code availability)

Open Access This Peer Review File is licensed under a Creative Commons Attribution 4.0 International License, which permits use, sharing, adaptation, distribution and reproduction in any medium or format, as long as you give appropriate credit to the original author(s) and the source, provide a link to the Creative Commons license, and indicate if changes were made.
In cases where reviewers are anonymous, credit should be given to 'Anonymous Referee' and the source.
The images or other third party material in this Peer Review File are included in the article's Creative Commons license,

unless indicated otherwise in a credit line to the material. If material is not included in the article's Creative Commons license and your intended use is not permitted by statutory regulation or exceeds the permitted use, you will need to obtain permission directly from the copyright holder.
To view a copy of this license, visit https://creativecommons.org/licenses/by/4.0/

Reviewer #1 (Remarks to the Author):

In this paper, Teng and colleagues show that humans exhibit a kind of "anti-Occam" phenomenon: they infer more latent clusters than exist in the data-generating process. The authors demonstrate this phenomenon repeatedly across a tour de force series of experiments. Computational modeling revealed that human behavior was consistent with the hypothesis that expansion of the hypothesis space (i.e., postulation of new clusters) is potentially unbounded but cognitively constrained.

Overall, I thought the main findings of the paper were really interesting, and the paper is overall well-written (though quite dense in some places). The modeling is done to a high standard. The paper has important implications for our understanding of inductive biases in human inference.

Thank you for your kind and insightful comments.

Major:

I find it a bit odd that in the Discussion the DEE model is framed in terms of conserving cognitive resources. I underestand that there is a resource parameter in the model, but it seems to me that the central empirical finding is that people are *less* economical than one would expect of a well-calibrated observer. They see more complex structure than is really there. I would appreciate if the authors could help resolve this tension in an intuitive way for the reader.

We agree with the reviewer that our previous Discussion was imprecise and oversimplified in framing the DEE model as solely conserving cognitive resources. Indeed, in the sense that it still leads to overly complicated structures, the DEE model does not seem to rationally save up cognitive resources. The word “economical” in the DEE model refers to its decaying tendency of model expansion at increasingly complicated structures, contrasting with the Chinese restaurant process that grows unboundedly. It is as if the brain starts to conserve cognitive resources when the remaining resources become low.

We have revised the related parts in Discussion (para. 1, p.14):

> Human performance is best modeled by a distorted economical expansion (DEE) process, [edit start] where an individual’s internal world model grows incrementally with new observations but at a decreasing rate when the model becomes increasingly complicated. It is as if a conservation mechanism is gradually activated when cognitive resources approach exhaustion.[edit end]

We have also clarified that the model is economical when the size is large in the motivation of the DEE model (p. 8):

> First, structure expansion must be *economical* to conserve humans’ limited memory, which is realized by a decay rate parameter $r > 0$ that slows down model expansion as the internal model becomes large.

and in the new Methods section (p. 24):

For $r > 0$, new clusters are less likely to be added as the model size grows.

It's not obvious to me that online inference (e.g., with a single particle) should yield a bias towards over-complication of the learned structure (as claimed on p. 14). I would want to see a concrete demonstration of proof of this claim. I think actually that there are some simple counterexamples, though this depends on details of the setup.

We agree with the reviewer that online inference with a single particle does not necessarily yield a bias towards over-complication of the learned structure. Our previous statement was based on the intuition that multiple particles allow the number of estimated clusters to decrease with samples (e.g., through sequential reweighting), while a single particle can only lead to an increasing number of clusters. However, consistent with the reviewer's insight, our simulation results (see the figure below) show that whether a single particle biases towards more or fewer clusters depends on the model parameters. In the revised manuscript, we have removed the inaccurate statement about single particles.

The figure below shows the parameters of σ_0 and a_0 and whether using a single particle produces fewer (blue) or more (red) clusters than using 1000 particles. The size of the dot indicates the absolute value of the difference. To get these results, we ran particle filter under the CRP model for T=50 samples drawn from a standard Gaussian. We fixed the prior parameters μ_0=0.0 and λ_0=0.1 (low confidence prior mean at 0) and randomly sampled σ_0 and a_0 over some relevant intervals. We used 1 or 1000 particles, repeated 1000 times. The colored dots below show that using a single particle can increase or decrease the estimated number of clusters depending on the hyperparameters, confirming the reviewer's intuition.

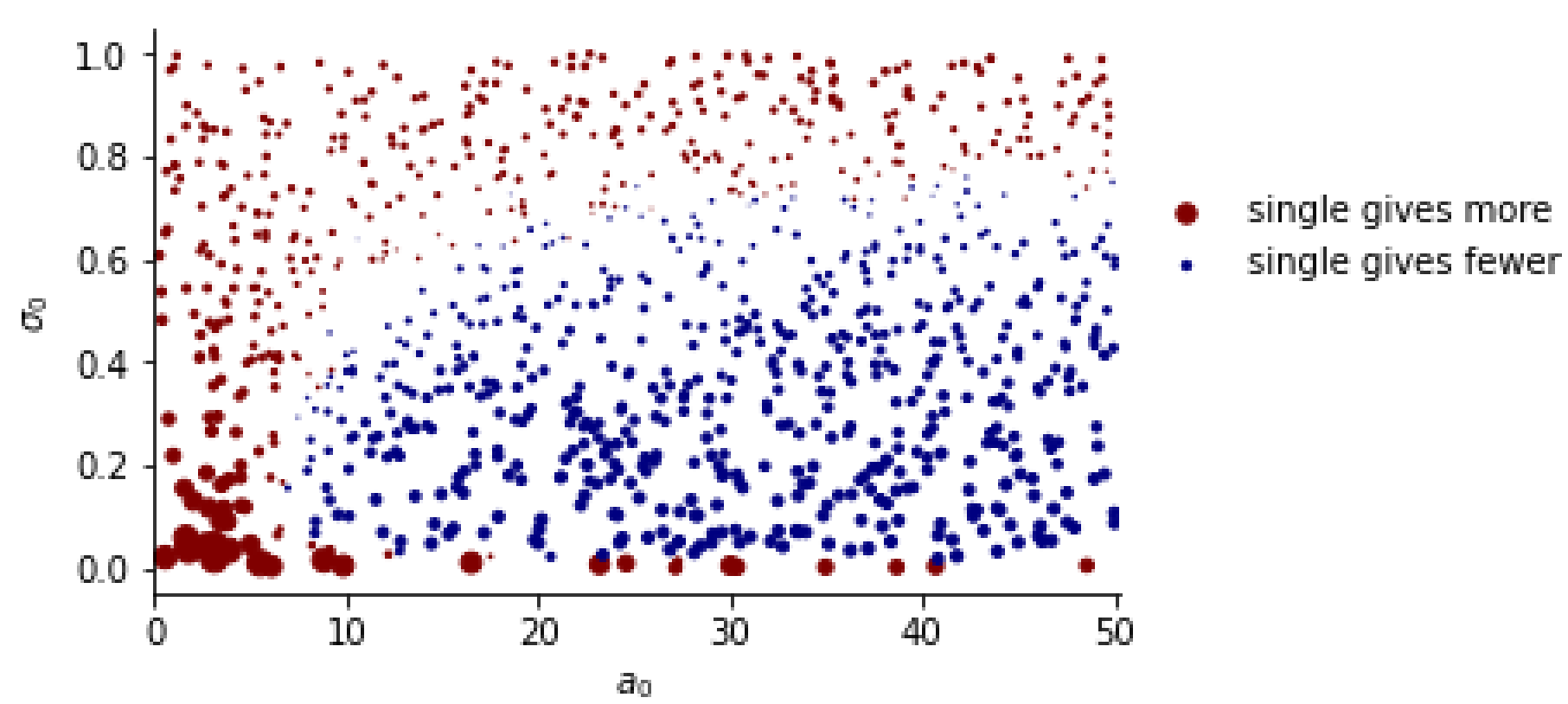

Since the models play such an important role in the paper, I don't think they should be completely relegated to the supplement, especially since I found it hard to understand them based on the description in the Results. I suggest the authors include the key technical details of the models in the Methods section.

Agreed. We have summarized the model details in the Supplementary material into shorter sections in Methods.

Minor:

The Methods section on Experiment 8 reports some experimental results. I think it would make sense to have all the results in the Results section, or possibly in the supplement.

Apologies for the confusion. We have moved the related experimental results to the Results section (pp. 13-14):

> This preference was significantly stronger in experimental trials (with carefully likelihood-matched options) compared to control trials where the multimodal lure had substantially lower likelihood ($t(215) = 5.03$, $p < 0.001$, 95%CI = [0.06, 0.13], $d = 0.34$, Figure S9C, see Methods for details).

Did the random-effects Bayesian model selection use the AIC, or an approximation of the log marginal likelihood?

Thank you for pointing out the missing content. We used the –AIC/2 as an approximation of the log model evidence (Eq. A.4 in Stephan et al. 2009). The related methods section is updated (p. 26):

> Additionally, we applied group-level Bayesian model selection as a complementary measure of model advantage, using –AIC/2 as an approximation of the log model evidence (Daunizeau et al. 2014; Rigoux et al. 2014; Stephan et al. 2009).

Reference:
Stephan, K. E., Penny, W. D., Daunizeau, J., Moran, R. J., & Friston, K. J. (2009). Bayesian model selection for group studies. NeuroImage, 46(4), 1004–1017. https://doi.org/10.1016/j.neuroimage.2009.03.025

p. 22: "handful parameters" -> "handful of parameters"
Corrected.

p. 23: I'm not sure what is meant by the statement "the approximate inference method using a single-particle no longer preserves exchangeability" since exchangeability here is a property of the prior, not the posterior (or its approximations).

The reviewer is correct that exchangeability is a property of the prior and independent of any inference method. We meant to say that using a single particle for inference over CRP does not make the posterior invariant to the order of samples in the sequence, while using a large number of particles (or exact inference) leads to order invariance. We realize that this is a technical detail that is not central to the main message of the paper, and runs the risk of unnecessary confusion, so we have removed this description on exchangeability in the revised manuscript.

p. 27: I think it's slightly misleading to say that the estimator in Eq. 14 is an approximation of the log-likelihood. As pointed out in the next section, Jensen's inequality implies that it's actually an approximation of a lower bound on the log-likelihood.

Agreed. More precisely, we approximate the log-likelihood using a biased (but consistent) estimator, and the bias should diminish with increasing number of simulations. We now explicitly state this when introducing Eq. 14 (now Eq. S12, p. 33), and refer the readers to the next section for the bias.

> Putting things together, we estimate the log-likelihood of data (with a diminishing downward bias, see next section) as …

To further demonstrate that such estimation bias is small and diminishes with the number of simulations, we ran additional experiments to confirm that the number of simulations we used to estimate the log-likelihood and AIC (1,000,000 simulations) should be sufficient to bring the bias to an acceptable range. We took the fitted models and ran {100, 1,000, …, 1,000,000} simulations to estimate the log likelihood. Below, we show the first-order difference in the estimated log likelihoods (log lik from 1,000 sims – log lik from 100 sims, log lik of 10,000 sims – log likelihood of 1,000 sims, etc.), plotted for CRP and DEE for all participants in Experiment 2. This difference is on the order of 0.01 at 100,000 simulations (could be negative due to randomness), and this curve goes down linearly on log-scales.

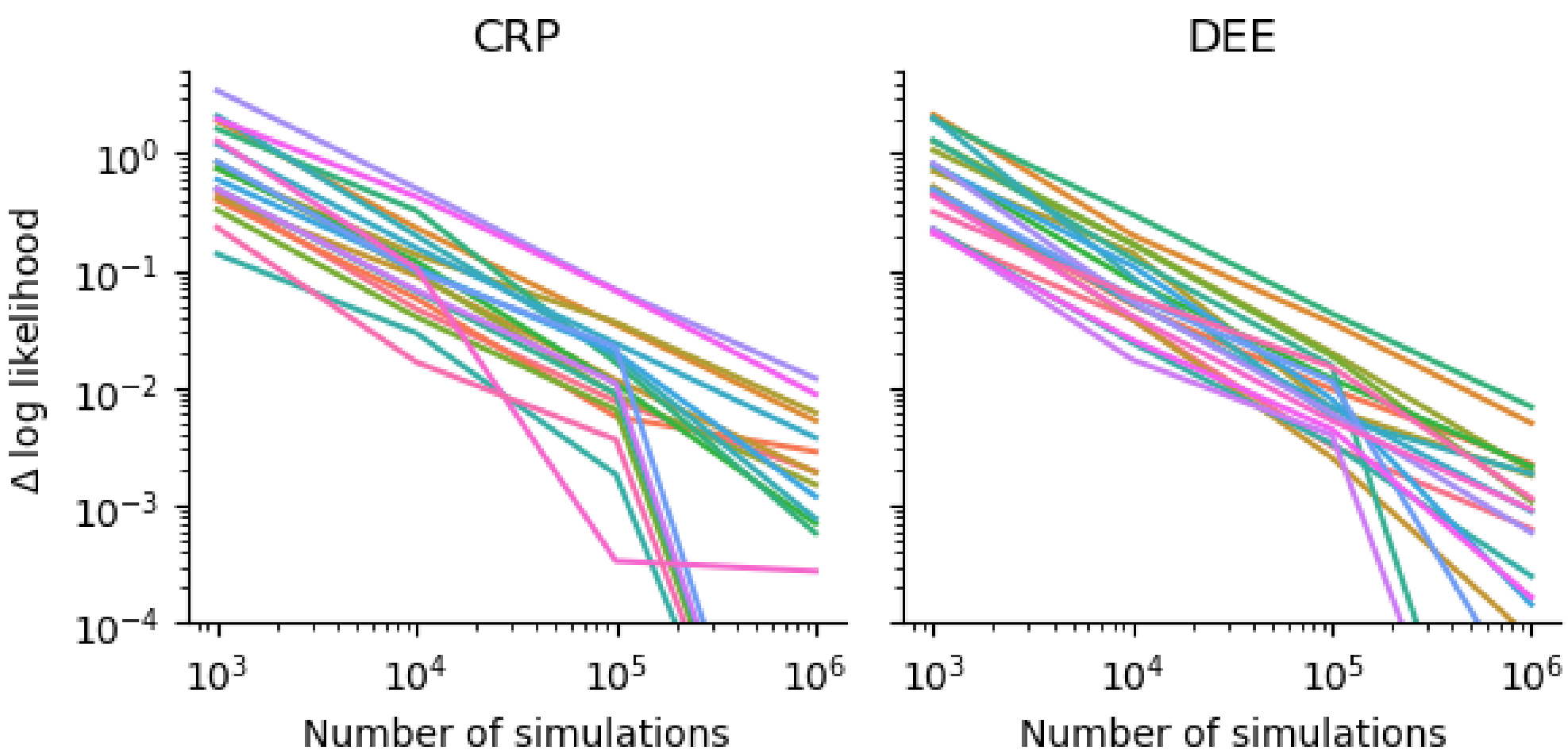

CRP
DEE
Δ log likelihood
10^0
10^{-1}
10^{-2}
10^{-3}
10^{-4}
10^3
10^4
10^5
10^6
Number of simulations
Number of simulations

Reviewer #2 (Remarks to the Author):

Review of "Illusory perception of latent probabilistic structure"
Reviewer: Michael Landy

This is quite a nice paper about how people build up a representation of a 1-d distribution, with the assumption of a Gaussian mixture model. The basic model, vastly simplified, is a forward process model matched to some of the experiments in which samples are shown to the observer one by one. The observer either puts the sample in an existing cluster (element of the mixture), updating its mean and SD, or creates a new cluster for it (with a default SD). Biases are introduced to reduce the likelihood of choosing to create a new cluster and to control cluster weights. The model never merges clusters, so that an overestimation of the number of clusters (when the true number is small) is kind of built in. The "economical" bias prevents models from getting too complex (and thus taxing structure memory in the observer). All in all the paper is impressive and convincing. I do have some comments, but this is very nice work.

Thank you for these insightful and detailed comments.

Specifics, important and trivial, mostly by line number:

Figure 1E: certian -> certain
Corrected.

General question:
If you showed all the samples simultaneously, I assume results would be different. So, this is really about the memory representation used when building a density estimate from sequential samples. If one sees all the samples at once, one can consider splitting a subset into separate clusters or merging them as a distinct, conscious decision. It's interesting that merging clusters is not needed for modeling the main task. But, the difference between your task and simple density estimation of a set of samples bears some discussion.

The reviewer has raised an intriguing question about sequential versus simultaneous presentation of samples, concerning whether (1) the behavioral biases and (2) the underlying learning algorithm found in our tasks could generalize to simultaneous presentation. We do not have definitive answers to either question. As suggested, we have added a paragraph in the Discussion stating indirect evidence from the literature as well as our conjectures.

Two points need clarification. First, although the DEE model based on one particle does not allow cluster merging during step-by-step cluster assignment, all our models for the structured density estimation task (including the DEE model) include an aleatoric component to account for noise in participants' reports, and one version of it does allow cluster merging (Aleatoric component in Supplementary Materials, pp. 31-32). This merging strategy did not capture human behavior as well as using the simple strategy of removing the smallest cluster. The main

behavioral patterns, including illusory perception of cluster numbers, are consequences of the DEE model's key assumptions: sample-dependent expandable prior, probability distortion, and economical model expansion.

The new paragraph added to the Discussion (p. 16) reads:

> We chose sequential item presentation in our task because it closely mirrors online learning under uncertainty in real-world settings (Anderson 1991; Sutton et al. 1998). An intriguing question is whether our major findings—the illusory perception of probabilistic structures and the learning process characterized by the DEE model—can be generalized to simultaneous presentation of samples, where working memory demands are reduced and deliberation on the structure is possible. The findings from a recent study make us conjecture that simultaneous presentation of samples may yield similar illusory perception: when estimating the mean position of a cloud of Gaussian-distributed dots, participants seem to group the dots into multiple clusters (Ota et al. 2025), resembling findings for sequentially-presented samples from multimodal distributions in earlier studies (Sun et al. 2019). It should be noted that simultaneous presentation does not necessarily entail parallel processing of all samples. For example, even estimating the statistics of a cloud of dots—commonly assumed to involve parallel processing—involves integrating information from sequential eye fixations (He et al. 2025), with sequential processing being even more likely for non-spatial features (e.g., shapes or numbers). Even when simultaneous presentation involves truly parallel processing, the DEE model could still apply, with minor algorithmic modifications—reframing the sequential random processes into equivalent parallel forms and replacing the particle filter with batch sampling methods such as Gibbs sampling (Sanborn et al. 2010). Our key model assumptions, including economical model expansion with sample size, should still apply because cognitive constraints would continue to limit the number of clusters used to represent distributions for future use, even when individual samples need not be memorized. Future studies are needed to test our conjectures.

Also, given that they had to report all cluster means, one by one, I wonder how much the results were do to the constraints on how participants made the report. Would it have been difference if, at any time, they could pick a cluster and delete it, move another, change its width, over and over until satisfied?

Participants were actually allowed to revisit a cluster and change its properties as many times as they wished, with no time limits. For Experiments 1 and 2 (visual modality), participants could change the weight and standard deviation (width) of each cluster. For Experiment 3 (numeric modality), where the report of standard deviation was not required, participants could change the mean and weight of each cluster. Though they had no option to directly delete a cluster, participants could effectively delete clusters by setting their weight to zero. We have supplied these important methodological details.

[p.19, Experiment 1] Participants could revisit any previously placed cluster by clicking its volcano icon and readjust both its weight and standard deviation. While cluster means could not be changed once initially placed, participants could effectively remove a cluster by setting its weight to zero.

[p.20, Experiment 3] Before their confirmation, participants were allowed to revisit the input fields and revise their responses.

Despite having this freedom, participants readjusted their reports in only a minority of trials (10.7% in Experiment 1, 13.8% in Experiment 2, and 36.7% in Experiment 3). Notably, participants never "deleted" clusters by setting weights to zero. Together, this suggests that the illusory perception of structures observed in our structured estimation task is unlikely due to constraints in our report paradigm.

Nevertheless, we understand the reviewer's concerns about the reporting format. This was exactly the motivation for our additional Experiments 4–8, which used completely different reporting formats but yielded converging conclusions.

Another thought: When presented with a sequence of random numbers, people see non-randomness that isn't there (e.g., sequences of identical digits or close real numbers seem surprising even though they are expected). Could clustering subsets be a bias in and of itself, in which case you'd also see too many clusters even with simultaneous presentation of the entire set, obviating the need for a sequential algorithm to explain it?

Thank you for these inspiring questions. We have discussed these issues in the newly added paragraph in the Discussion (p. 16, quoted in our reply to your previous question).

In short, we agree with you that clustering subsets could be a bias in and of itself, even with simultaneous presentation of all samples. However, as we argue in the manuscript, the DEE model may still shed light on the learning process underlying such bias. On one hand, simultaneous presentation does not necessarily imply parallel processing. On the other hand, at the computational level, the key assumptions of the DEE model—sample-dependent expandable prior, probability distortion, and economical expansion—can be separated from its sequential algorithm implementation.

Figure 1C: The legend (or the figure) should say which row is which, rather than assuming the reader will notice the colors and look at the key above 1B.

Clarified in the figure legend:

Top and bottom rows are respectively for sample size 10 and 70.

Legend 1F: "reports were centralized relative to the mean of ...". I find that phrase obscure, so please clarify.

We jittered the center of the generative distribution on a trial-by-trial basis. To better use the limited figure space for visualizing the shape of the reported density, we shifted the x-axis by subtracting the sampled jitter from each x-coordinate, centering each panel at the true mean of the latent distribution.

We have rephrased the sentence (p. 5):

> To enhance visualization, each panel is centered at the mean of the true generative distribution.

272-275: This is a pretty minimal definition of similarity between report and the twin trial only including number of clusters and nothing about the set of (mean,sd,weight) triples.

Indeed, the similarity between the twin trials manifests in almost all aspects we can measure. We have now presented more measures in Figure S3D (Gray and red colors respectively code data and DEE predictions). For most of these metrics, it is more natural to define them as differences between the reports (e.g., the earth-mover distance between the densities), so we plot all metrics in terms of distance.

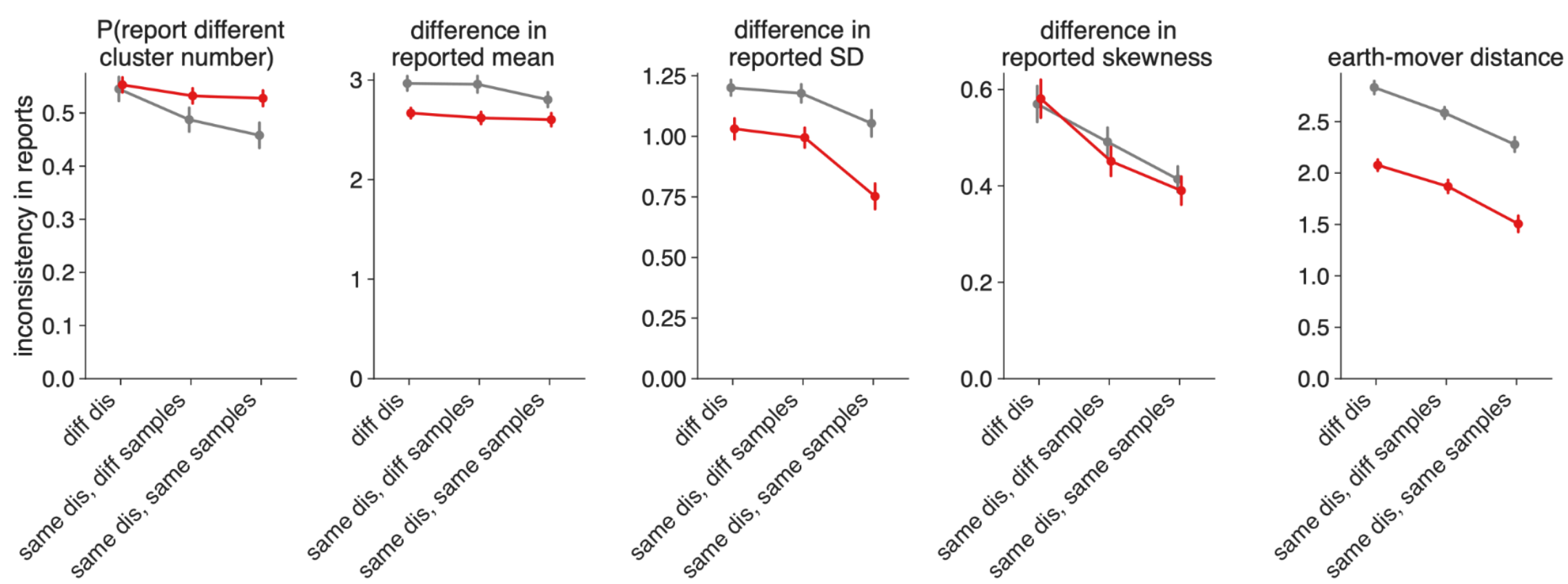

Fig. S3, … D, Inconsistency in participants' reports across three comparison conditions: (1) different sample sequences from different distributions ("diff dis"), (2) different sample sequences from the same distribution ("same dis, diff samples"), and (3) identical sample sequences ("same dis, same samples"). Inconsistency is measured by: probability of reporting different cluster numbers, absolute difference in reported moments (mean, SD, skewness), and earth-mover distance. For earth-mover distance, reported densities are centered at the mean of the true generative distribution (as in Figure 1G) to isolate shape differences beyond location shifts.

Figure 3A: Desnity -> Density, Schoastic -> Stochastic
Figure 3C (twice): gets richer -> get richer
400: unobserved to -> unobservable by
437: modulated by memory resource -> modulated by limited memory resources
Thank you for your careful reading and pointing out these typos. We have corrected them.

444-445: I don't really buy the tasks for Expts. 4-8 as being a different requirement for the participant. Sure, they don't have to explicitly state the number of clusters. But, they do need to build up a representation and then compare it to options that have a clear number of clusters.

We are afraid that the description “options that have a clear number of clusters” does not apply to Experiment 5, where options were presented as raw samples rather than probability density functions with visible clusters. For instance, in the option example for Experiment 5 in Figure 4B (reproduced below), it is not immediately apparent how many clusters are present in the samples shown in the first, second, and fourth rows. It is not even obvious that these options represent distributions with different numbers of clusters.

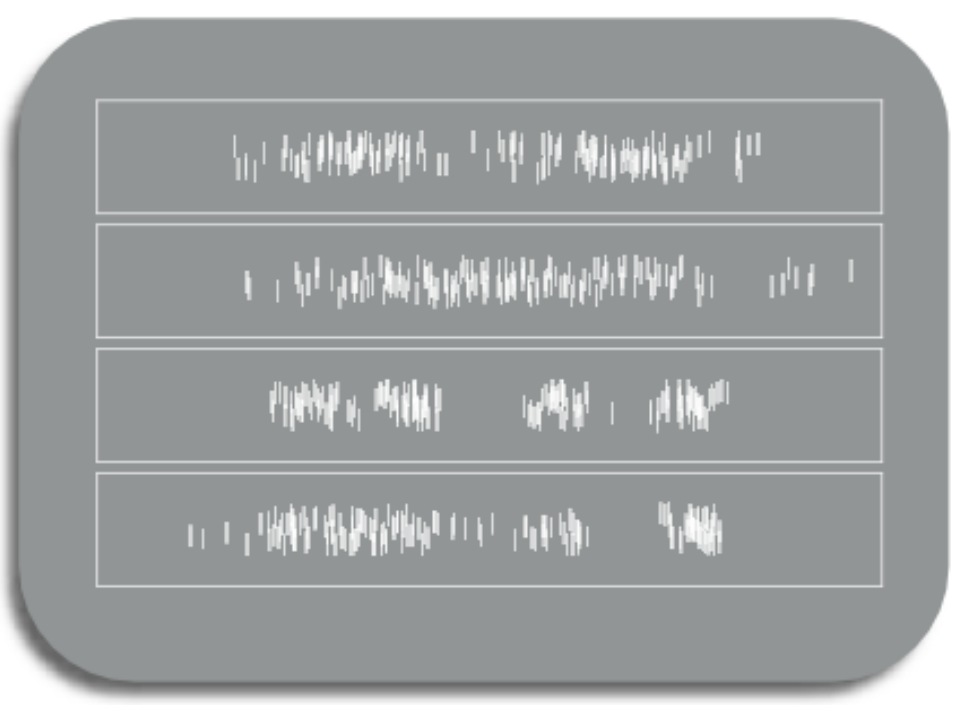

619: manifests -> manifests
We have corrected the word by changing it to the singular form.

721: "participants cluster property reports". First, it needs an apostrophe: participants' reports. But the phrase "cluster property report" is needlessly obscure.
We have rephrased this sentence to be more comprehensible (p. 19).

> As participants adjusted cluster properties, a blue curve was updated in real time to show the marginal distribution of the Gaussian clusters.

Expt. 3: Was this a different set of participants from Expts. 1 and 2. Otherwise, they might have been prompted to think about the task visually despite the numeric stimuli. In fact, given the task, it seems likely that many might have used an internal visual number line to do this task. Did you ask?

Yes, each of eight experiments recruited a different set of participants. We have added this information to the Methods (p. 18):

> Each experiment recruited a different set of participants.

We did not ask participants whether they used an internal visual number line to perform the numeric task. We agree that some participants might use an internal spatial line to organize numeric representations. However, the distribution of reported cluster numbers for Gaussian samples in the numeric experiment (the unimodal-Gaussian panel in Figure S4A) differs from comparable conditions in the visual experiments (Figure 3E). For example, at sample size 70, four clusters were most frequently reported in the numeric experiment, while three clusters were most frequently reported in the visual experiments. This suggests that the two modalities are subject to distinct cognitive constraints and the illusory perception of probabilistic structures found in both modalities is unlikely to be explained by the use of a common visual number line.

974: Why are you using \Sigma instead of \sigma for a 1-d SD in the notation?
This unconventional notation is used for two reasons. First, we later add superscript to this term (e.g., Eq. S2), using σ for standard deviation will cause unnecessary clutter ($\sigma^{2,i}$). Second, the variance appears more frequently in equations than the SD for the rational component, so to avoid confusion, we use Σ to distinguish from a standard deviation and is closer to the variance (or a 1x1 covariance matrix). Later on in the aleatoric component, we switch to the standard deviation σ. We have now clarified this notation in p. 24 (following Eq. 2) in the new Methods section. We have also added the following sentence in the Supplementary Materials (p. 30):

> Note that we use Σ to represent the variance of a cluster, and σ the standard deviation.

975: I assume \Delta^K is meant to mean the K-dimensional simplex (k-tuples that are positive and sum to one), but I'd bet many readers won't know that, so clarify, please.
Yes, it is. We have added the definition for \Delta^K as suggested (p. 27).

1000: handful OF parameters
Corrected.

1004-1005 and elsewhere: I'd use the standard term "lapse" rather than "slack" throughout. Note that this "lapse" is a flat distribution over an infinite domain (i.e., improper).
Agreed. Changed to “lapse” as suggested.

Eq. 2 and 1028: \alpha_t is proportional to the probability of making a new cluster. Thus, that probability is reduced when r is large and positive, the opposite of what you say on line 1028.
Corrected.

1032: From here down, the text is pretty much for the cognoscenti and very dense. If you want this more widely read, you'll need to add some introductory material and some clarify. First, I don't remember what "exchangeable" means in this context.
We agree that this is a very technical point and is confusing while not adding much to our main message. It is then more adequate to remove the discussion on exchangeability. We have also included a more introductory background on the normal-chi-squared distribution in Supplementary Material, and have elaborated on the generative nature of the DEE process (pp. 28–29).

1034: "single-particle" doesn't need a hyphen here. More important, the use of the word "particle" comes out of the blue. In effect you have a forward process model that could be run a bunch of times in parallel, like a particle filter, but you never say that out loud. So, put this in a context so the reader knows where you are going with it.

We now introduce the single-particle idea this way: participants can use a particle filter algorithm for approximate inference -> particles are “approximate samples” -> a single particle is usually sufficient from previous work -> In our case, we approximate the cluster assignment by a single particle.

There is a subtle difference between using many particles and using a single particle repeated over many *simulations*. We do the latter. We explain the single-particle approximation to the reader early, and then say these are latent randomness of the inference (forward) process unobserved to the experimenter, which needs to be averaged out by Monte Carlo simulation.

To make things clear, we have explicitly defined the superscripts i as indicating the i-th run of the forward process/simulation required for likelihood approximation. In addition, we now explicitly state the assumption that participants keep a single cluster assignment for each past observation, equivalent to using a single particle of assignment trajectory (p. 8):

> We assume that participants keep a single possible cluster assignment for each past observation.

We also appreciate the suggested phrase “forward process”, which is now used in the main text to summarize our algorithm (p. 10):

> Taken together, our overall framework is a modular, flexible and cognitively plausible forward process that produces structured densities given sequential observations, allowing us to test diverse hypotheses about how participants perform the task; this enables the discovery and validation of deeper cognitive insights from more complicated forms of report.

1042: I didn't re-educate myself on this. I thought the standard model was normal-inverse-gamma (for mean/variance). Maybe normal-inverse-\chi^2 is the standard thing if you use SD instead of variance (I don't know and didn't check). But again, this is not knowledge that your readers will know, so be more didactic as you lay things out.
Normal-inverse-gamma and normal-chi-squared distributions are reparameterization of the same distribution. The latter has more interpretable hyperparameters, which we now explain for clarity (pp. 28-29). This will give our readers more background. We also relate the posterior parameters in Eq. S2 with simple averages introduced above Eq. S1, to make Eq. S2 more interpretable.

1051: \bold{z}_t^i: what is "i"??? You never say and I couldn't figure it out from the context. I'm wondering if the "i" throughout is an index for possibly multiple particle runs. Maybe yes, maybe no. I'm lost at this point with your notation.
Our apologies! The index refers to the *i*-th simulation of the single particle forward process. We have added an explicit definition of *i*. This superscript differentiates a particle from the underlying random variable without the superscript; second, it refers to the *i*-th particle filter run, with each run producing a single particle over the cluster assignment sequence (so does not require an additional index). Clarification has been added to this paragraph (p. 29).

> Different runs of the particle filter over a given sequence may produce different cluster assignments, which are used for likelihood approximation; see Section Monte Carlo simulation later for details. Different runs of the particle filter over a given sequence may produce different cluster assignments, which are used for likelihood approximation; see Section Monte Carlo simulation later for details. Thus, we also use the superscript i to indicate the i'th single-particle sequence from a collection of particle filter runs $z_{\square}^{1}$, $z_{\square}^{2}$, …

1054: Either "over Gaussian mixture parameters" or "over the Gaussian mixture parameter"
We changed it to "over cluster properties" to be consistent with other mentions.

1067: I have no idea what "(unadjusted)" means here
It refers to the biased finite-sample estimator (divided by "N" rather than "N-1"). Sanborn et al. (2010) used the unbiased estimator, and we want to be clear here. We have now added "biased" in the parentheses (p. 30).

1094-1095: \mu_{T,k} gets a superscript i in one instance and not in the other???
Good catch. This is now corrected with i added in both cases (p.30).

1100-1102: As I said above, this statement is the crux of your model and is hugely important, but is buried in math methods in the Supplement and never discussed explicitly in the main text.

"It renders a single hypothesis over the cluster assignments of the past, while randomly assigning the incoming stimulus xt according to (5). As such, previous assignments cannot be

modified, even if new observations may favor other assignments. Maintaining multiple particles allows revision of past assignments when new stimuli arrive."

Sanborn et al. (2010) noted that using a single particle prevents cluster reduction, which we referenced here and hypothesized that this could contribute to over-estimation of the number of clusters. To test whether this is the case, we ran inference simulations on CRP of various prior parameters (σ_0 and a_0), and compared the number of clusters estimated by 1 particle or 1000 particles. The result indicates that whether using a single particle increases or decreases the estimate depends on the prior cluster properties; see the figure below.

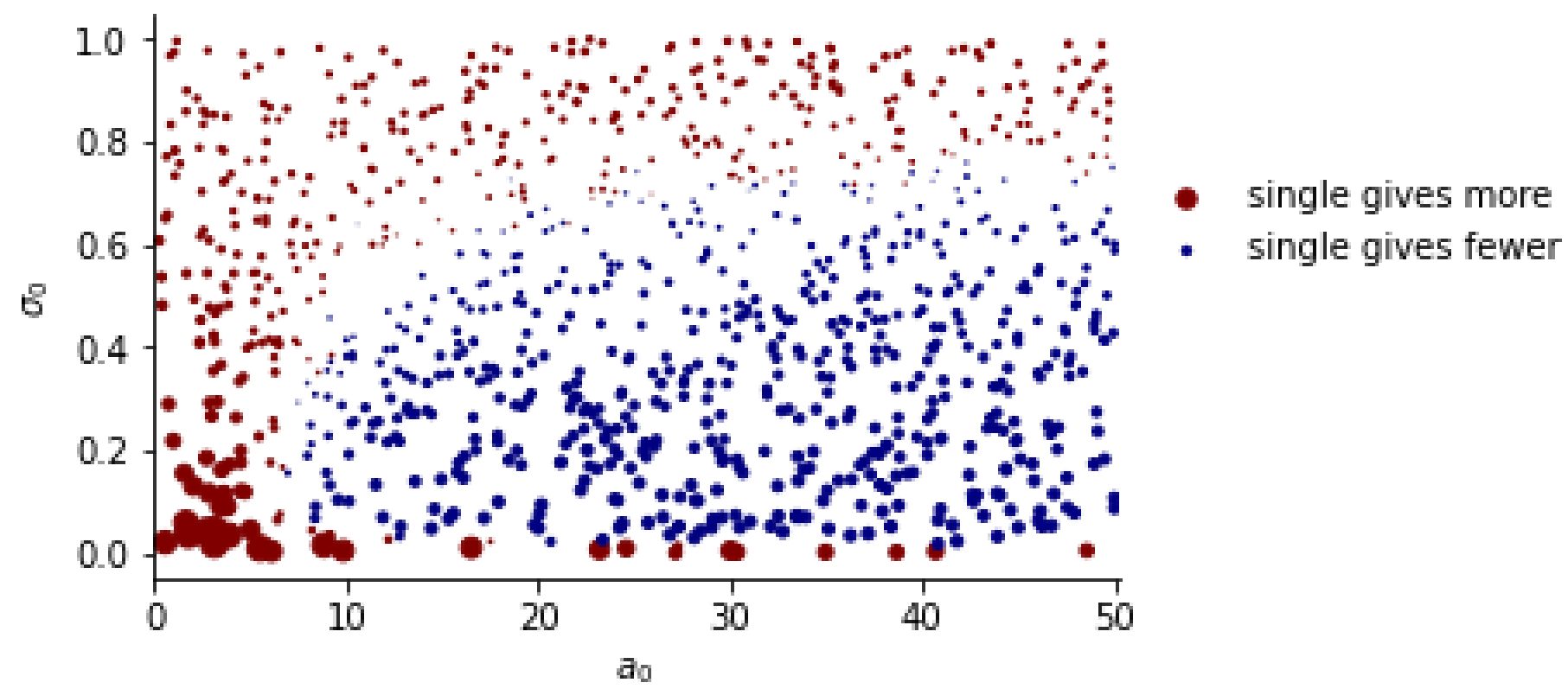

This is a nuanced detail of the CRP. We also found no improvement in the quality of fit when using more particles (up to 20). As such, we have removed the discussion on how a single particle can lead to under- or over-complication of the estimate structure.

1111: number of clusterS
Corrected.

1134/1138: I think you have the cases backward, case 1 is for K-tilde > K-hat (so that you have to merge) and case 2 is the opposite.
Corrected. We note that the best aleatoric component used the removal strategy instead of the merging strategy ("Merge Cluster" ablation Supplementary Material, p. 35, Figure S6).

1262: I may have missed it, but the acronym "DEE" seems only to be spelled out in a figure legend and not in the main running text.
Thanks for the reminder. The acronym is introduced in the main running text along with its full name (p. 9): "We thus refer to this process that generates structured density as the Distorted Economical Expansion (DEE) process."

1286: Our -> Out
Corrected.

1314-1315: "the function f(\phi,\hat{K}) merge the smallest cluster" doesn't parse and I'm not sure what you meant to say here
This is now clarified as “the function f(\phi,\hat{K}) combines the smallest cluster (in terms of the cluster weight) with its nearest neighbor (in terms of the mean) to form a new cluster.”

Figure S2E right-hand plots: Isn't this negative correlation inevitable for two clusters? If they are to match the SD of the samples, then when they are further apart they have to reduce their SD to compensate. The result seems built-in.

We believe the right-hand plots of Figure S2E are informative because the negative correlation between cluster SD and eccentricity is not a pattern that every model can predict. For example, as the figure below shows, the var-GMM model can hardly predict this negative correlation, though the DEE and CRP models can predict it well.

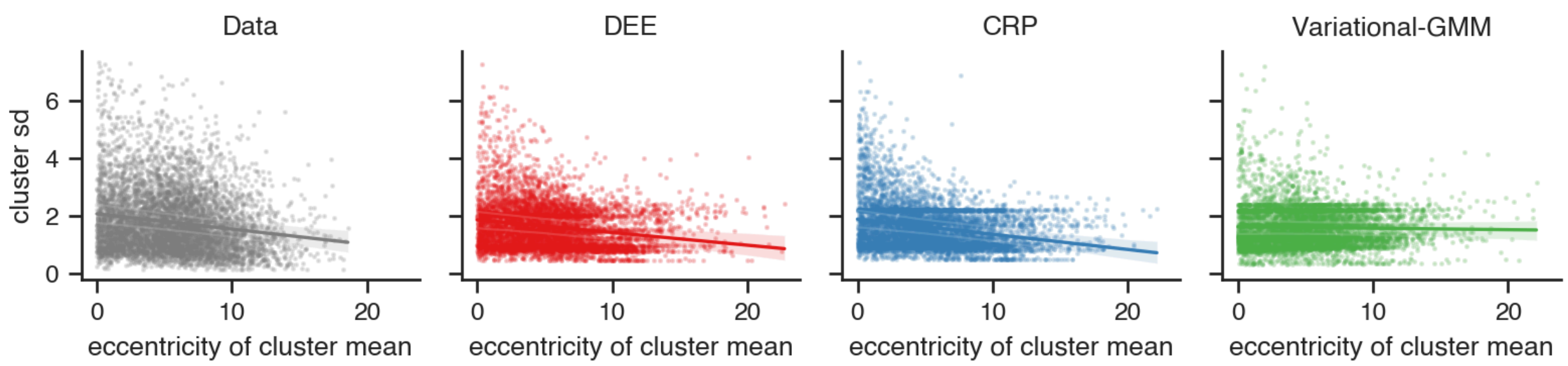

We agree with the reviewer’s intuition that attempting to match the overall SD of samples could produce a negative correlation between cluster SD and eccentricity. However, we do not think the negative correlation observed in our data is simply an artifact of this matching strategy, for several reasons. First, as described above, not all models can explain the phenomenon. Second, despite its high correlation with the ground truth, the overall SD of participants’ reports showed considerable biases and variances (Figures 1C and S2C), suggesting that participants did not strictly match the overall SD of samples.

Based on the DEE model, we conjecture that the observed negative correlation is partly driven by the uneven assignment of samples to clusters in the center versus the periphery. The reported clusters at the tail of the distribution, which may have no counterparts in the ground truth, are likely to absorb only a few samples and thus have smaller cluster SD and weight. In contrast, clusters near the center, which are less eccentric and absorb more samples, have larger cluster SD and weight. Consistent with our conjecture, there is a positive correlation between cluster SD and weight (see Figures S2E, S3G, and S4C).

Figure S&: You plot the correlation for a parameter recovery. But, for recovery you don't just want a high correlation, you want the scatterplot to be close to the identity line. Seems the wrong statistic to describe how close you achieved that. Why not include some scatterplots (with identity lines) of simulated vs. recovered?

We agree with the reviewer that it is important to check whether the recovered parameters match the generative parameters. We now include a scatter plot of recovered versus generative parameter values as a supplementary figure (Figure S7, reproduced below). For most parameters (including all three parameters highlighted in the main text, *r*, *β*, and σ_0), the dots fall on the identity line.

In preparing the plot, we detected a small bug in the Jupyter notebook for the codes of model recovery, whose influence on the results is negligible. After correction, the summary statistic of correlations reported in the main text is updated from 0.75 ± 0.18 to 0.76 ± 0.18 (p. 10).

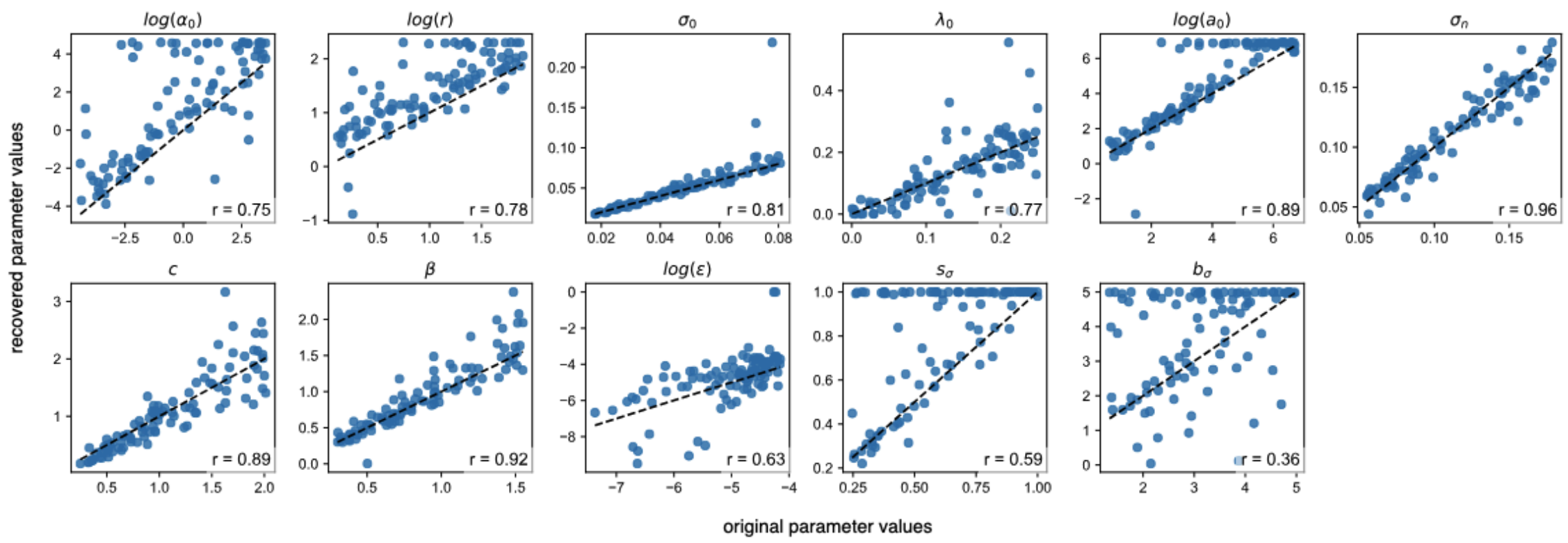

Fig. S7 ~~Model~~ Parameter recovery. We simulated responses from the DEE model using 100 randomly sampled parameter pairs, with trial settings identical to those in Experiment 2. The parameters were drawn uniformly from the range defined by the minimum and maximum values of the fitted parameters in Experiment 2. The noise levels in the final report of the cluster mean and standard deviation (Equations (9) and (10)) were set to the median of the fitted σ_v and σ_m. We then refitted the DEE model to each of the 100 simulated datasets and computed the correlation between the true (generative) and recovered (fitted) parameters.

1836* The probability of CHOOSING
Corrected.

Various figures: axis label should be "# of reported clusters"
We have corrected "reported cluster" to be "reported clusters". To save space, we did not add "of" in the axis label. Complete descriptions are included in the figure legends.

Reviewer #2 (Remarks on code availability):

N/A

Reviewer #3 (Remarks to the Author):

Teng, Li, and Zhang examined people's internal representation of latent structure underlying sequentially observed data. Using a structured distribution report task, the authors have found that although people could report the probability density relatively well, they fail to identify the true underlying structure. The authors have developed a model termed distorted economical expansion process (DEE), to capture people's behavior in both the self-report task and a series of AFC tasks. The DEE model builds upon the Chinese restaurant process (CRP) with additional parameters as well as an aleatoric component to accounting for cognitive constraints and randomness in participants' behavior.

The research question is important and the task design is novel and suitable to probe people's internal representation. In principle, the finding that the same sorts of biases that impair decisions from description also lead to systematic biases in understanding the structure of the environment, would certainly be novel and of lasting importance to a broad audience. However, we believe that more analysis of both human and model behavior are needed to substantiate that claim – and that more discussion is necessary to clarify and contextualize it.

Thank you for your comprehensive and highly constructive comments.

Main comments:

One question we had in trying to understand this paper is whether the behavior metrics that are focused on reflect structural understanding, or a probabilistic decision strategy used to report it. More specifically, whether economic biases impact structural understanding – or whether economic biases impact a series of one-off decisions through which that understanding is communicated in this particular task framework. Participants were not able to "undo" a mixture component once it was specified. If you consider that the decision about where to place the first "cluster" might be both probabilistic and systematically biased, as we would expect from existing decision making literature, and that the subsequent decision would be made conditional on the first, couldn't you get many of the reported results even with perfect underlying knowledge of the structure? Would this idea extend to the series of 2AFC task results that were also reported? The manuscript does not currently grapple with the distinction, and to our view, it needs to in order to support the primary claim that the biases measured arise at the level of a structural understanding, rather than from the decisions based on it.

The reviewer's comments regarding "structural understanding" and "series of one-off decisions" raises a crucial point. To make sure our response is targeted, we have provisionally defined "structure understanding" as the structured density learned by participants given observations before reporting. We also take the "decision" (and the "series of one-off decisions") to mean the actions performed during the final report stage, where participants sequentially report various cluster properties via an interactive display. We respond below based on these working definitions.

Several lines of evidence suggest that the biases we observed in the structured density estimation and distribution recognition tasks likely arise from structural understanding rather than from the sequential post-learning decisions based on it. First, our finding that the reported cluster number increases with sample size suggests an evolution of biases during learning, which cannot be attributed to post-learning decisions.

Second, as clarified in the revised manuscript, participants in the structured density estimation task were actually allowed to revisit a cluster and change its properties as many times as they wished, with no time limits. Though they had no option to directly delete a cluster, participants could effectively delete clusters by setting their weight to zero. This freedom to revise earlier decisions makes random errors in the decision process less likely to accumulate into systematic bias. We apologize for any confusion in our previous manuscript, and have provided clarification in the Methods (p. 19).

Third, though the two types of tasks require distinctively different forms of responses, similar mis-estimation of cluster numbers was found in both tasks, indicating that the biases originate from structure learning, which is common to both tasks. Notably, the distribution recognition task tested in Experiments 4–8 required only simple forced-choice judgments, involving no sequential reports like "a series of one-off decisions". Still, participants' responses were strikingly biased. For example, in the 2AFC task of the pre-registered Experiment 8, participants consistently favored multimodal lures over the correct Gaussian option.

Fourth, the results of Experiment 5 provide additional evidence that the observed biases are unlikely to arise from the forced-choice decisions. In Experiment 5, options were presented as raw samples rather than probability density functions with visible clusters, so similar biases would apply to both the stimuli and options if the biases originated from the decision phase. Therefore, the observed biases are more likely to arise from structural understanding rather than from the decisions based on it.

The reviewer could also refer to potential biases in the sequential decisions of cluster assignments. In fact, the sampling approach is correct and *unbiased* given the choice of using a single particle algorithm. Some of our ablated models in Supplementary Materials add decision biases on top of this, but they all gave worse results in terms of AIC:

1) removing the sampling noise by greedy cluster assignment (Local MAP)
2) using the first assigned sample as the mean of each cluster (Fixed Mean variant)
3) using a constant value for all the cluster widths (Constant Variance)

These results suggest that participants follow the inference algorithm quite closely, at least not exhibiting the biases above during cluster assignment decisions.

We agree with the reviewers that the origin of the observed biases is an important question to address. In the revised manuscript, we have added a paragraph in the Discussion (p. 15):

The biases in the reported structured density more likely stem from the learning process rather than from subsequent task-specific decision-making processes. Participants reported more clusters as the sample size increases, indicating that the bias evolves during learning, which cannot be explained by a single post-learning decision policy. The illusory perception of clusters is observed across both the structured density estimation and distribution recognition tasks, suggesting that the biases have arisen before being revealed in different report formats. In particular, the distribution recognition task tested in Experiments 4–8 required only simple forced-choice judgments that are less affected by report biases; further, in Experiment 5, the options provided were presented as raw samples comparable to those of the stimulus, making judgments further immune to potential distortions in the decision phase.

The key behavioral features in the human subject data – and how these features are affected by the parameters of interest in the DEE model – were not sufficiently clear to evaluate the strength of evidence for each individual economic bias contributing to behavior. The manuscript overall seems a bit vague on what the key behavioral pattern is in the structured distribution report task. In the abstract section, the authors made the claim that people typically overestimate the cluster size. Though this seems true when # of true cluster <= 2 with a small sample size (figure 1D), this is not the case when # of true cluster >=3. It also seems that the reported cluster number does not differ much when the underlying cluster number changes (Figure S2B). In discussion, the author wrote '[participants] struggled to identify the true generative structures even with extensive observations.' The overall behavioral claim needs to be more clear and quantified by specific conditions throughout the manuscript. Furthermore, the behavioral analyses focus on two experimentally manipulated factors (number of clusters, number of samples) and show that the former has no influence on reported number of clusters, while the latter does. While these findings are enough to falsify key predictions of a CRP, they are not particularly informative with regards to what subjects are actually doing, nor are they sufficient to explain why the particular biases in the DEE model might be necessary to explain the behavior. Presumably, there are some more systematic trends in human behavior that are captured by the models – for example that specific experimental mixture distributions tend to elicit more reported clusters – and perhaps these trends could shed light on why specific components of the model are necessary to capture these. Our view is that fully supporting the primary claim would require examining the factors that systematically influence structural reports and showing how key parameters of the model allow it to reproduce the influence of those factors on behavior.

Two points were raised by the reviewer. We address them under two subheadings below.

Demonstrating how DEE's key assumptions contribute to behavioral patterns

Two additional analyses were conducted: first, we systematically varied the key parameters in DEE to demonstrate how they influence key behavioral metrics in Experiment 1. We have also included a figure for illustrating the difference between DEE, CRP, and Variational-GMM, which may address the reviewers' later concerns. Although our computational model and our report

data structure are both very complicated, we show these results having controlled the model parameters at typical fitted values, and grouped the results into different experimental and behavioral facets, such as the number of true clusters and number of samples. We hope this will strengthen the connection between our model and behavioral data, and show where DEE performed better than the alternatives.

(1) The effect of DEE parameters on behavior

We systematically varied three key DEE parameters:

- r (which controls the decay rate of expansion tendency);
- β (which controls probability distortion); and
- σ_0 (which controls the prior over cluster width).

We then examined three behavioral metrics after learning the density:

- the reported number of clusters;
- the entropy of reported cluster weights; and
- the overlap between reported clusters, calculated for every trial.

The entropy of reported cluster weights reflects the probability distortion: higher entropy indicates that reported weights across clusters are more similar (due to downweighting large clusters and upweighting small clusters), with maximum entropy achieved when all reported clusters have equal weights. Our simulations reveal distinct parameter effects (Figure S10, p. 49):

- r strongly influences the **number of learned clusters** but has minimal impact on other behavioral aspects (Figure S10A);
- β primarily modulates **reported weight entropy**, reflecting probability distortion (Figure S10B);
- and σ_0 simultaneously affects multiple behavioral dimensions (Figure S10C).

(2) Visualize DEE's behavior and compare it to CRP and DEE.

To further illustrate the contribution of these key assumptions on capturing behavior, we compare in Figure S11 (p. 50) the predictions made by DEE, CRP, and Variational-GMM on the reported number of clusters (K) and the reported weight (w).

- DEE better captures K than CRP and Variational-GMM (Figure S11B), especially in Experiment 1 with 70 samples and Experiment 3. For Experiment 2, all models performed similarly and DEE leads by a small margin.
- DEE captures the entropy of the reported w distribution than CRP (Figure S11A). Also, both CRP and Variational-GMM tend to produce clusters with small weights (Figure S11C), a feature not so prominent in the data. This is especially the case of Variational-GMM in Experiment 1.

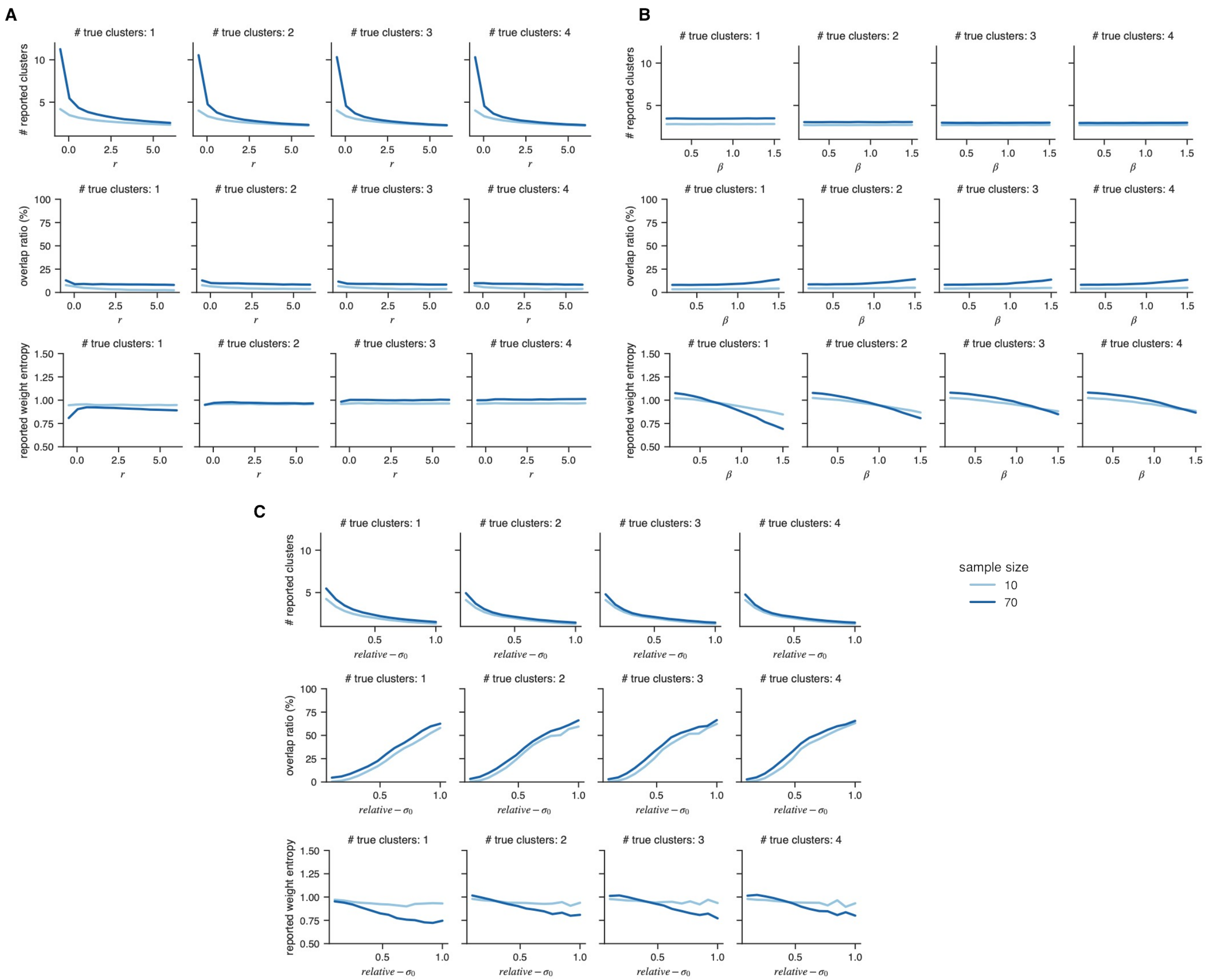

Fig. S10 Illustration of model behavior as function of key parameters. We simulated model-predicted behavior from Experiment 1 across a range of parameter values. For each trial, we computed the entropy of the reported cluster weights to quantify the certainty in the weight distribution within that trial. Higher entropy indicates more uniform weight distributions across clusters. Overweighting small probabilities and underweighting large probabilities will result in a higher weight entropy. In each panel, one parameter was varied while all other parameters were fixed at the median values fitted in Experiment 1. The overlap ratio between clusters and the reported weight entropy can be substantially influenced by the reported number of clusters. To ensure that the reported number of clusters does not confound the illustration of weight entropy and overlap ratio, we only included trials in which the model reported 3 clusters while plotting these two behavioral metrics. **A**: The economical expansion parameter r strongly modulates cluster number growth. As r increases, the number of reported clusters decreases for both sample sizes (10 and 70). At high r values, the difference in reported cluster number between the two sample sizes diminishes, indicating that r constrains sample-driven expansion due to memory limitations. In contrast, r has minimal influence on cluster overlap ratio and reported weight entropy. **B**: The probability distortion parameter β primarily affects reported weight entropy, with minimal influence on the number of reported clusters or their overlap ratio. **C**: The prior cluster width parameter σ_0 (relative-σ_0) influences all behavioral metrics. For consistency with Figure 3C, σ_0 is normalized by the standard deviation of the marginal distribution in Experiment 1. A relative-σ_0 of 1.0 indicates that the prior cluster width equals the marginal distribution's standard deviation. Smaller relative-σ_0 values lead to more reported clusters, lower overlap between clusters, and higher weight entropy.

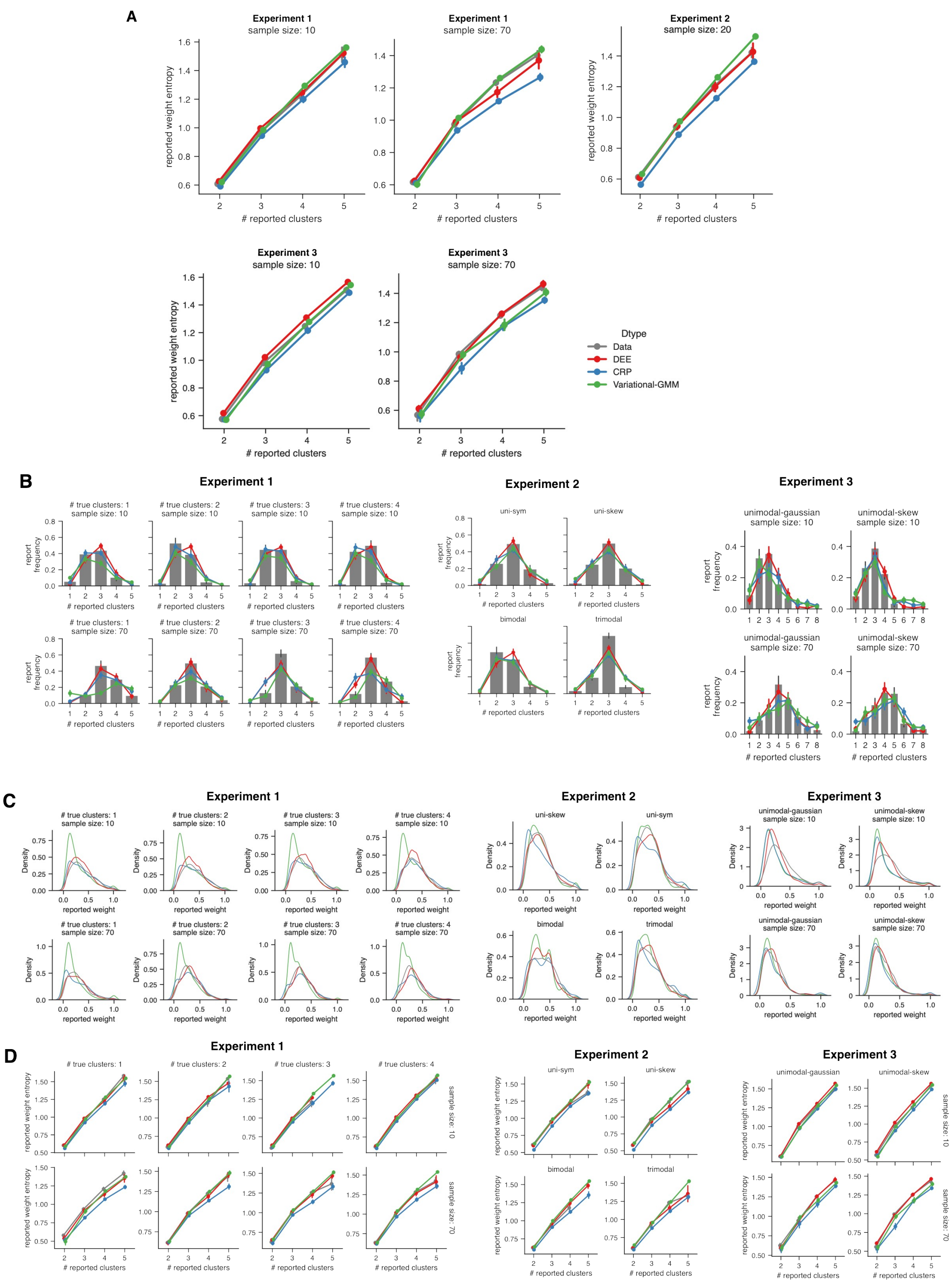

Fig. S11 Comparing DEE's behavior to alternative models. **A**: Reported weight entropy versus number of reported clusters. Weight entropy measures how evenly distributed cluster weights are (higher entropy = more equal weights). DEE and Variational-GMM match human patterns well, while CRP consistently underestimates entropy, indicating that probability distortion is necessary beyond basic CRP. **B**: Distribution of reported cluster numbers across experiments. **C**: Distribution of reported cluster weights across experiments. **D**: Weight entropy (from A) faceted by the type of true distribution.

Summarizing the key behavioral patterns

We thank the reviewer for this suggestion. One paragraph is added to the Results section to enhance the main message while mentioning the key behavioral finding in Experiment 1. The abstract and the result section were updated to be more precise.

> [Results, pp. 7-8]: To summarize, in both visual and numeric modalities, we found systematic over-complication in the structured distributions learned by humans when the true distribution was simple. Instead of converging to the ground truth, *increasing* observations from 10 to 70 samples led participants to report *more* clusters. However, participants did not produce arbitrarily complex structures, reporting fewer clusters when the true distribution exceeded three clusters. The reported clusters were well-separated, resulting in profiles often bumpier than the true generative distributions. Given the complexity of the task and these behavioral patterns, inferring inductive biases directly from summary statistics is difficult. We thus resort to building computational models to simulate such behavior and identify key parameters that underlie online density estimation by humans.
>
> [Abstract]: …Across eight behavioral experiments (including one pre-registered study) in two modalities, participants made systematic errors in reporting the probabilistic structures, despite estimating overall probability density reasonably well. For example, participants consistently overestimated the number of clusters when the true distribution was a single Gaussian but underestimated it when the true distribution contained many clusters.…
>
> [Discussion, p. 14]: While participants could approximate the overall density, [edit start] they often failed to identify the true generative structure, and seeing more observations resulted in an increase in the reported number of clusters.[edit end]

Along the same lines, providing more details for other aspects of the behavioral data could be helpful. For example, what is the distribution of weights people put on different clusters? The authors have reported skewness but it only captures people's report on a trial aggregated level. Is the weight distribution different under different conditions (# of clusters, sample size)?

We now include two new ways of visualizing the reported weights in Figure S11 (see figure in the last reply). Together with our existing visualizations (Figures S2E, S3G, S4C, showing joint distributions of cluster weight, sd, and location), Figure S11 provides additional details on the distribution of weights. Figure S11C plots the distribution of all reported cluster weights across different distribution types and sample size levels. Figure S11A&D show the entropy of reported clusters to visualize the participants' tendency of reporting weights of similar magnitudes. Note that the S11C reflects the distribution among all reported clusters, while the S11A&D reflects the entropy of weights in specific trials.

Note that the magnitude of the reported weights in a trial is strongly influenced by the number of reported clusters in that trial, and given that the number of reported clusters changed in different conditions, the distribution of reported weights of all clusters was also influenced by experimental conditions. As our focus is on probability distortion, the reported weight entropy provides more information about our research question. We can see that DEE is better at capturing the reported weight entropy, while CRP usually underestimates it.

The major contribution of the DEE model, as far as we understand, is to incorporate cognitive constraints into the clustering process. The authors have done a comprehensive model comparison to show the superiority of DEE (Figure 3D and Figure S6) focusing on model fit metrics, but it is not clear how these model components change its behavior. How does behavior of the model change when parameters thought to reflect different cognitive constraints are turned up or down? Do specific constraints affect specific aspects of the measured behavior? And are these aspects of behavior clearly related to systematic biases in structural understanding? It would also be very helpful to see figures that overlay model prediction and human data for the other model candidates similar to Figure 3E-G, but for a wider range of behavioral metrics.

Thanks for these valuable suggestions on clarifying the relevance of specific model assumptions to behavior. See our replies to a similar concern raised by other reviewers. As suggested by the reviewer, we have added Supplementary Figure S10 (p. 49) to demonstrate the contribution of each key model parameter to behavior, and Figure S11 (p. 50) to visualize model prediction versus human data, comparing different models for a wider range of behavioral metrics.

The computational model plays a pivotal role in supporting the primary claim made in this paper. There is no way to evaluate the evidence for the claim without understanding the model. Thus it is our view that the modeling methods (or at least the critical ones) should be included in the main paper rather than the supplementary information.

Agreed. A new modeling section is now included in the main text.

Other comments:

The primary novel claim of the paper, at least to our understanding, is that cognitive biases lead to systematic structural misunderstandings. Given this, it seems critical that the discussion contextualize the results in terms of the cognitive biases explored here and how they relate to the larger context of work in decision making on cognitive biases – for example, we were very surprised not to see any mention of Prospect theory in the discussion, despite one of the core components of the model being a subjective probability much like that incorporated by prospect theory.

We agree with the reviewers that the probability distortion in our DEE model is similar to that in Kahneman and Tversky's prospect theory. However, we chose not to cite prospect theory, for several reasons. First, the concept and mathematical formulation of probability distortion was first proposed in the perception literature in the 1960s (Pitz, 1966), predating prospect theory. Kahneman and Tversky introduced probability distortion (weighting functions) in their original prospect theory paper (Kahneman & Tversky, 1979) but did not propose a specific mathematical form until their cumulative prospect theory paper (Tversky & Kahneman, 1992). Even within the decision-making literature, Karmarkar (1978, 1979) and Goldstein and Einhorn (1987) proposed functional forms for probability weighting earlier than Kahneman and Tversky (see Zhang & Maloney, 2012 for a review). Second, the specific probability distortion function we used is different from that of Tversky and Kahneman (1992). Third, the potential origin of probability distortion in our context aligns more closely with the perception literature than with typical prospect theory applications, where probabilities are stated explicitly.

Nevertheless, we appreciate the reviewers' suggestion to situate our findings in a broader context and have enhanced the discussion accordingly. See our reply to the reviewers' next question. We welcome suggestions of other related work.

Pitz, G. F. (1966). The sequential judgment of proportion. *Psychonomic Science*, 4, 397–398.

Karmarkar, U. S. (1978). Subjectively weighted utility: a descriptive extension of the expected utility model. *Organizational Behavior and Human Performance*, 21, 61–72.

Karmarkar, U. S. (1979). Subjectively weighted utility and the Allais paradox. *Organizational Behavior and Human Performance*, 24, 67–72.

Goldstein, W. M., and Einhorn, H. J. (1987). Expression theory and the preference reversal phenomena. *Psychological Review*, 94, 236–254.

Zhang, H., & Maloney, L. T. (2012). Ubiquitous Log Odds: A Common Representation of Probability and Frequency Distortion in Perception, Action, and Cognition. *Frontiers in Neuroscience*, 6. https://doi.org/10.3389/fnins.2012.00001

Discussion should also provide broader context of other paradigms that have been used to measure how people conceptualize probability distributions, and how the current paradigm might differ from these. For example, DiBerardino 2024 provides a very rich way to characterize people's beliefs about probability (specifying a complete distribution), whereas Nassar 2010 provides a much more constrained one (just mean and variance). This context seems somewhat necessary for interpreting the broader claims of the paper, in particular, whether the biases observed are related to structural understanding, rather than how it is reported. In principle, the bias to see more clusters than truly exist would be consistent with the overly high "hazard rate" seen in Nassar 2010, since considering a new changepoint is essentially adding a new latent cause.

Thank you for recommending these valuable references. We had cited Nassar et al. (2010) in our original manuscript but did not realize its connection to illusory perception of clusters. We have enhanced the second paragraph of the Discussion (pp. 14-15), describing the methods and findings of other paradigms in more details:

> One striking finding is that humans persistently misinterpret Gaussian distributions as more complex structures, typically seeing them as mixtures of barely overlapping clusters. Though seemingly counterintuitive, this finding does not necessarily conflict with previous studies that suggest humans prefer simpler, unimodal representations (Flannagan et al. 1986; Nisbett and Kunda 1985; Tran et al. 2017), where multimodal representations (especially those deviating from the ground truth) were not systematically tested, even when the visualized data hint multimodality (Tran et al. 2017). In fact, our findings of overcomplication in learned structures are hinted at in seminal modeling works on category learning (Anderson 1991; Sanborn et al. 2010) and align with findings from a wide range of behavioral paradigms. Some studies (Zhang et al. 2015; Schustek and Moreno-Bote 2018) ask participants to make decisions based on the integral of probability distributions and use computational models to infer participants' internal representations, revealing that participants form multimodal representations even for samples from Gaussian-like distributions. Another study (Nassar et al. 2010) uses computational modeling to infer how participants estimate the mean and variance of samples in dynamic environments and finds that humans overestimate the probability that changes occur—a tendency toward overcomplication since each changepoint introduces a new latent cause. Two recent studies (Nie et al. 2021; DiBerardino et al. 2024) use histogram-like reports to obtain more model-free and richer measures of participants' probability distribution representations. Through spectral analysis (Nie et al. 2021) or classification analysis (DiBerardino et al. 2024) on the high-dimensional reports, both studies indicate multimodal features in prior beliefs, at least for some participants. In contrast to these findings that rely on indirect behavioral measures, auxiliary model assumptions, or complicated data analysis methods, our structured density estimation task provides more direct evidence for humans' illusory perception of latent probabilistic structures. Converging evidence from the distribution recognition task further demonstrates that such illusory perception is automatic, not depending on explicit requirements of structure learning.

In Figure 3G, how is the eccentricity of cluster mean calculated? Is Eccentricity of cluster mean defined as distance from the mean of the full mixture distribution? If so, doesn't the weight have to be negatively correlated with this – since a higher weight would also pull the mean toward the cluster. If this is a key qualitative effect that the model captures – it shouldn't be something that is tautological.

Apologies for the confusion. The eccentricity is calculated as the distance between the reported center and the mean of the presented samples. Thus, the definition of "center" is not influenced

by any of participants' reports, thus relieving us from the concern raised by the reviewer. We now made the definition of eccentricity clear in the legend of Figure 3.

It is interesting to see the impact of sample size on human reports. However, it does not seem to be motivated enough in the introduction - why are we interested in people's reports under small and large sample sizes? Presumably this effect alone is enough to falsify the CRP, which is an exchangeable model? Also, can the DEE model reproduce the results in Figure 1D?

Motivation for sample size manipulation:
Testing on different sample sizes is implicated by our focus on sequential learning, an ethologically relevant setting, and is explicitly motivated in the first paragraph under the subheading "Illusory perception of latent structures": it allows us to observe the effect of different strengths of distributional evidence, without interrupting a trial and asking for a complex distributional report multiple times. We have added the following sentences (p. 4) to motivate it better:

> Varying the sample size avoided interrupting each trial to query for a complex distributional report and also allowed us to observe convergence patterns, if any.

By including multiple sample size levels (along with manipulating the true distribution type), we can better constrain our modeling of the learning process. Indeed, we found a surprising phenomenon through this manipulation: when the truth is a simple Gaussian, more samples did not attract participants' beliefs toward the truth, but instead pushed them further away from it. The inclusion of different sample size levels (and different types of true distributions) also helped to falsify CRP, as DEE better captures the sample-driven expansion process from small to large sample sizes.

Whether DEE can reproduce the data pattern in Figure 1D: Yes, DEE successfully captures the pattern in Figure 1D (see the plot below, reproduced from Figure S2D, where gray bars and red dots respectively denote data and DEE predictions). We had originally omitted this comparison to avoid redundancy with Figure S2B, which shows the same information in greater detail across all conditions. We have now reformatted Figure S2D to directly show this comparison, matching the layout of Figure 1D.

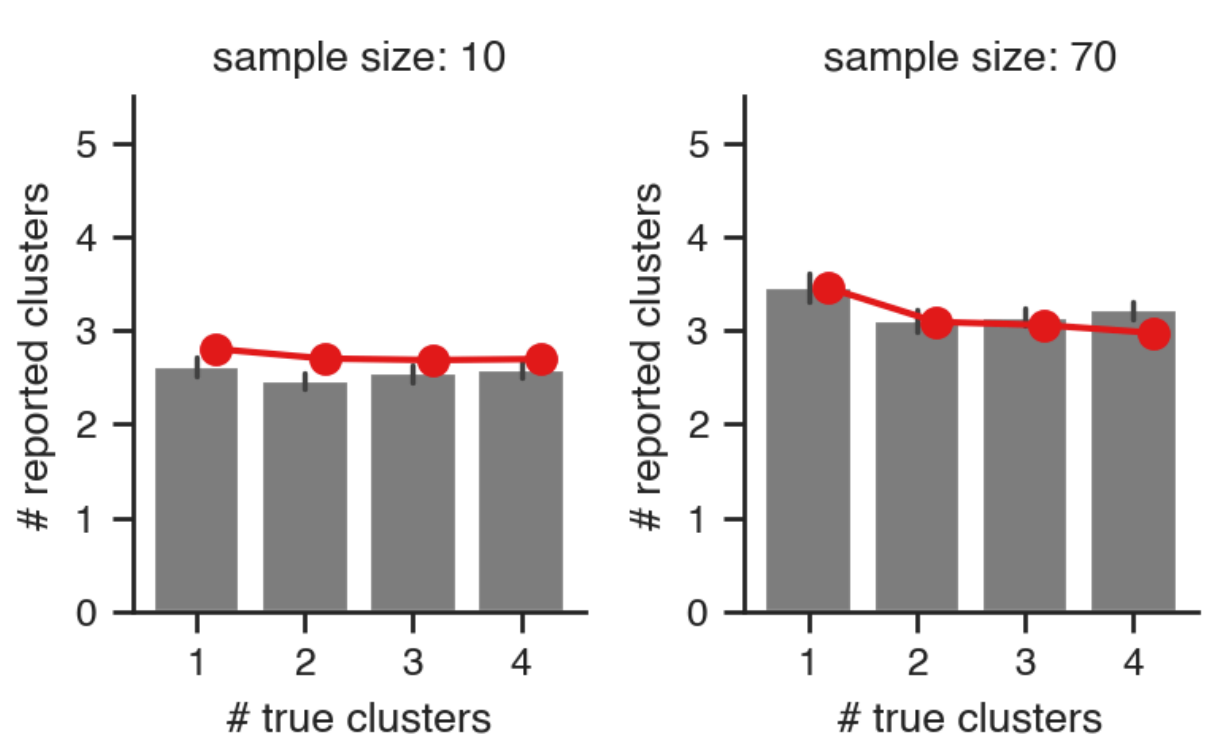

Experiment 2 results suggest that people are relatively inconsistent in reporting cluster numbers when facing the identical observation stream. It would be helpful to provide more discussions on this point. For example, can the DEE model reproduce this inconsistency? If not, does this suggest that there are other sources of randomness that the aleatoric process fails to account for?

We use this inconsistency as a baseline randomness of the reported density, but our DEE model is still limited in reproducing these highly detailed randomness in the data, despite that the mathematical formulation of this is already complicated. Doing this would require a much more flexible model (including a wide class of aleatoric components) and more deliberate trial structures, such as querying multiple reports at different time points during observation (Findling et al., 2019) or detailed manipulation of task type and sample size (Drugowitsch et al., 2016). We added discussion about the noise structure in the paragraph for limitations and future directions (p. 17):

> On capturing within-participant behavioral variability, although our model could reproduce some of the variability exhibited in human structure learning reasonably (Figure S3D), residual mismatches remain. Future studies could query more reports per sequence from participants and vary the sequence length more systematically (e.g., Findling et al. 2019; Drugowitsch et al. 2016) to differentiate and model these noise components more precisely.

Findling, C., Skvortsova, V., Dromnelle, R., Palminteri, S., & Wyart, V. (2019). Computational noise in reward-guided learning drives behavioral variability in volatile environments. *Nature Neuroscience*, 22(12), 2066-2077.
Drugowitsch, J., Wyart, V., Devauchelle, A. D., & Koechlin, E. (2016). Computational precision of mental inference as critical source of human choice suboptimality. *Neuron*, 92(6), 1398-1411.

For model comparison, is the CRP and/or variational-GMM model equipped with an aleatoric process as well? Otherwise, it does not seem to be a fair comparison.

Yes, all rational components / structural learning models are equipped with an aleatoric component, described in the last sentence of *Batch-based variational GMM* in the supplementary materials. We also added a line to make it more explicit (p. 35).

> We fit all parameters to each participant's data using the exact same procedure of DEE fitting, after augmenting the batch-based model with the same aleatoric component used in the DEE model.

Authors should report the instructions of experiments 1 and 2 more in detail - the instructions seem important in setting up the experiment and we are not sure how well people understand the action of reporting several gaussian distributions.

Agreed. The instruction of Experiment 1 and 2 is now included in the Supplementary Materials (p. 40):

> Participants were instructed that their task was to predict the distribution of future samples by inferring the properties of latent clusters. Samples were described as "magic lava stones" emitted from "invisible volcanoes", and participants were told their predictions would help the Magic Council deploy energy collection fields optimally. Participants were told that their performance would be evaluated based on how well their drawn distribution graph predicted the rate of occurrence of future samples.
>
> Participants were explicitly told they needed to infer three latent properties of each invisible volcano:
>
> 1. Location: where each volcano is positioned
> 2. Emission range: how widely stones spread around each volcano
> 3. Activity level: how frequently each volcano emits stones relative to others
>
> These three properties correspond to the parameters of Gaussian mixture components (means μ_k, standard deviations σ_k, and weights w_k, respectively). All instructions were framed using intuitive, non-statistical language. The instructions used visual examples to show that stones from each volcano follow a bell-shaped distribution centered at that volcano's location, without introducing terms like "Gaussian", "normal distribution", or any mathematical formulation.
>
> As participants adjusted their reports during the response phase, the overall marginal distribution was updated in real time as a blue curve on the screen. This real-time visualization allowed participants to immediately see how their reported cluster parameters combined to form the predicted distribution, enabling them to iteratively adjust their estimates to better match their internal representation of the distribution.

Figure 3D should specify what \delta AIC means in the caption.

Agreed. The following is added to the caption (p. 9):

> ΔAIC represents each model's AIC relative to the best-fitting model for each participant, then averaged over participants.

Figure S6 should specify the meaning of different colors.

Agreed. The following is added to the caption (p. 45):

> In all panels, CRP is shown in blue. Green bars indicate models that differ significantly from CRP on that metric (Wilcoxon signed-rank test, $p < 0.05$), while grey bars indicate no significant difference.

Figure S7 caption should be ‘parameter recovery’ instead of ‘model recovery’.
Agreed. It is now corrected (p. 46).

Reviewer #4 (Remarks to the Author):

I co-reviewed this manuscript with one of the reviewers who provided the listed reports. This is part of the Nature Communications initiative to facilitate training in peer review and to provide appropriate recognition for Early Career Researchers who co-review manuscripts.

Thank you for co-reviewing the manuscript and providing valuable feedback.

Reviewer #1 (Remarks to the Author):

The authors have done a good job addressing my comments. I'm happy to recommend publication.

> Thank you for your helpful comments!

I don't want to make a big deal about this, but I don't think -AIC/2 is technically a proper approximation of the log model evidence. This is true for -BIC/2 asymptotically (see for example the derivation in the Bishop 2006 textbook), but as far as I know this doesn't hold for AIC, which is derived from different (non-Bayesian) principles. Maybe the correct thing here is just to say that the authors use this as a model score but don't commit to the Bayesian (marginal likelihood) interpretation.

> Agreed. We have revised the sentence in concern, “using –AIC/2 as an approximation of the log model evidence”, to be “using –AIC/2 as the model score” (p. 25).

A few small things:
- p. 10: a phrase ("this enables...") is repeated twice.
- p. 17: "there is not explicit, step-wise merging operation" -> "there is no explicit, step-wise merging operation"
- p. 24: "memory laod" -> "memory load"
- p. 24: "Previous work have established" -> "Previous work has established"
- p. 25: "estimated number of cluster" -> "estimated number of clusters"
- p. 25: "The parameters of the chosen model is taken" -> "The parameters of the chosen model are taken" ... "which is" -> "which are"

> Thank you for the careful reading. These are all corrected.

Reviewer #2 (Remarks to the Author):

Re-review of "Illusory perception of latent probabilistic structures"
Reviewer: Michael Landy

I still like this paper and it's a bit more readable now, which is a good thing. I now fully understand the models, although I did still get a bit lost in some of the details in the Supplement. My comments are trivial. This thing is ready to go.

Thank you very much for your comments and support.

160: "see dashed lines": This is the only reference to the two contours in 1F. The legend should state what black vs. blue is.

Sorry for the omission. The legend of Figure 1F has been updated to:

> "Examples of the structured densities participants reported (blue solid) in response to the observations (gray dots) drawn from hidden underlying distributions (gray dashed)."

174: "effect of true cluster number": From the figure, this seems to be driven entirely by the stimuli with a single cluster

You are correct. Post hoc tests confirm significant differences between the 1-cluster condition and all other conditions (vs. 2-cluster: $t(20) = 5.52$, $p < 0.001$; vs. 3-cluster: $t(20) = 4.12$, $p = 0.003$; vs. 4-cluster: $t(20) = 5.41$, $p < 0.001$), with no significant differences among the 2-, 3-, and 4-cluster conditions (2 vs. 3: $t(20) = -0.164$, $p = 0.998$; 2 vs. 4: $t(20) = 0.327$, $p = 0.988$; 3 vs. 4: $t(20) = 0.493$, $p = 0.960$).

However, we respectfully note that our primary objective is to demonstrate the effect of sample size as a sanity check for the principle of "gradually acquiring density information from more samples." The manipulation of cluster number was included for completeness, but it is not central to our main point of the paragraph. Given this focus, we believe that presenting the full suite of post hoc comparisons would draw undue attention to a secondary aspect of the design and potentially obscure the main message. We therefore prefer to retain the current presentation without these additional statistical details.

349: "DEE" should be spelled out at first use (probably here), not at 353

Apologies for not fixing it properly. We have reordered the sentences, and how the first mention of DEE is spelled out (p. 8).

> "First, structure expansion must be economical to conserve humans' limited memory… The effects of these two parameters are shown in Figure 3C and Figure S10, together with the effect of the prior cluster width $\sigma 0$. We thus refer to

this process that generates structured density as the **Distorted Economical Expansion (DEE)** process.

435: As I said last time, I think, "r" is not the right statistic. You don't merely want the recovered parameter to be linear in the true parameter; you want them to be close to equal (slope 1, intercept 0).

Agreed. We now remove the ‘r’ from the main text as reporting it alone could be misleading. Figure S7 now includes the slope and intercept of the linear function, and the figure legend is updated accordingly.

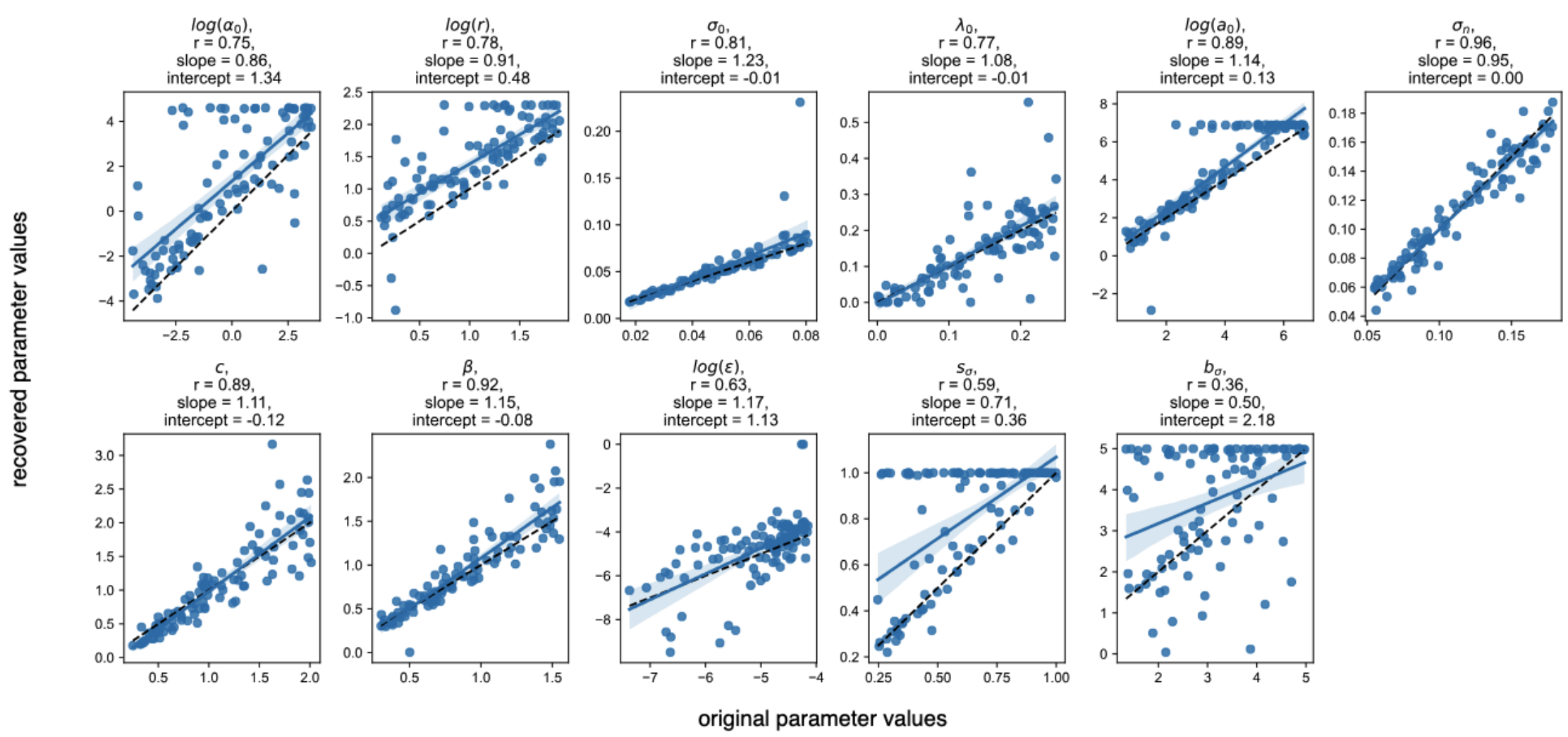

Fig. S7 Parameter recovery. We simulated responses from the DEE model using 100 randomly sampled parameter pairs, with trial settings identical to those in Experiment 2. The parameters were drawn uniformly from the range defined by the minimum and maximum values of the fitted parameters in Experiment 2. The noise levels in the final report of the cluster mean and standard deviation (Equations (S9) and (S10)) were set to the median of the fitted σ_v and σ_m. We then refitted the DEE model to each of the 100 simulated datasets and computed the correlation between the true (generative) and recovered (fitted) parameters. The mean correlation between simulated and recovered parameters was 0.76 ± 0.18 (SD), with mean linear slope of 0.98 ± 0.22 and mean intercept of 0.49 ± 0.75.

Eq. S5: The probabilities of the two cases should be 1-\epsilon and \epsilon. There's no need to remove \hat{K}_i from the 2nd case. Leaving it in just corresponds to a slightly different value of \epsilon.

Thanks for pointing out the confusing and alternative formulation. For clarity, we have updated this to express exact probabilities of each outcome, instead of a proportionality.

Now, ε is the lapse probability equally distributed among all possible number of clusters from 1 to K_{max}.

754: not explicit -> no explicit
756: bit fit -> better fit?
1070: number of clusters -> number of items
1074: laod -> load
1115: cluster -> clusters
1117-1118: sample -> samples
1324: a the -> a
1487: creates a lapsed -> creates lapsed

Thank you for pointing out these typos. These are all corrected.

Reviewer #3 (Remarks to the Author):

We thank the authors for their responses to our comments. We especially appreciate figures S10 and S11, which have linked the parameters in the DEE to behavioral metrics and show that DEE does a better job than other model candidates to qualitatively capture people's behavior. Overall, we think the paper has been strengthened and clarified. However, despite this, we still have few remaining concerns with the interpretation of the results and its communication.

• In our initial review, we raised the concern that the observed bias may partly arise from the sequential structure of the task (i.e., reporting the cluster location and its properties in sequence). While the authors provide new evidence supporting that the effect generalizes across experimental conditions and variants, there are still some remaining questions regarding the origins of some of the primary effects. For example, the authors note that it was possible for participants to effectively eliminate a cluster by setting its weight to zero – however, the fact that participants never do so could be interpreted as evidence for them conditioning subsequent inference on this initial response, which would be a form of the sequential effects that we were concerned about. Basically, we worry that sequence based explanations exist for many of the individual results and assuming that these cannot be rejected directly through analytic approaches, they should be discussed in a more balanced fashion.

Beyond the sequential effects driven by the task design, it is also quite possible that individuals undergo a series of cognitive decisions, leading to sequential effects related to internal, rather than external, decisions processes. This applies to the 2AFC task, as participants may internally engage in a sequential evaluation before committing to a binary choice. We are not requesting that the authors directly resolve this issue, as doing so would likely require substantial and creative changes to the task design. However, we believe the newly added discussion paragraph should be more cautiously framed and more explicitly acknowledge that some of the observed effects may stem from (mental) sequential cluster reporting.

We thank the reviewer for clarifying the concern and for the encouragement to discuss sequential effects. In the density estimation tasks (Experiments 1-3), it is not possible for participants to report a structured density in a single action, so sequential effects are possible. But, as implicitly agreed by the reviewer's, there is no behavioural sequentiality in the distribution recognition tasks (in Experiments 4-8) where the

decision is reported via a single button press, and thus biases from sequential reporting alone cannot explain the biases there.

The reviewer's further suggestion that some form of sequential cognitive (tacit) decisions, even in single-click decisions, may cause the observed biases raises an intriguing possibility. Although it is in general challenging to verify or reject such a thought process, our aleatoric component can be viewed as capturing the said process, and we have determined the best cluster manipulation strategy from other possibilities (p.31, below Equation S5). However, other rational components (e.g. CRP, GMM), when equipped with this aleatoric component, could not explain the data as well. Thus, the main factors that best accounted for the data are not in the sequential decision.

We have updated the additional discussion paragraph and encourage future work to explore this direction (p. 15).

> … Further, in Experiment 5, the options provided were presented as raw samples comparable to those of the stimulus, making judgments further immune to potential distortions in the decision phase. What is more, our aleatoric component can be regarded as a model for sequential cognitive decisions, and is the best from a few different cluster manipulation strategies. Meanwhile, other rational components could not account for the data as well as the DEE when they were augmented with this aleatoric component. In future studies, the aleatoric component could be extended to include other cognitive processes to further investigate any sequential decision biases, possibly under a task where participants are shown a target density (no dentistry estimation) for which they then need to sequentially report.

• It would still be valuable to mention prospect theory in the manuscript. Although the authors provide several reasons for why it may not apply, we see little downside to briefly citing and discussing it. Given the conceptual overlap, some readers (including ourselves) are likely to draw connections between the present work and prospect theory, and an explicit clarification of the similarities and differences would therefore be helpful.

We have now cited Tversky & Kahneman's (1992) prospect theory and briefly described it (p. 8).

> …, a form of distortion similar to that applied to probabilities in decision under risk (e.g., Tversky and Kahneman 1992)

• Our concern regarding inconsistency in human behavior has not been fully addressed. The authors note that “our model could reproduce some of the variability exhibited in human structure learning reasonably; residual mismatches remain.” While we do not expect the current model to fully account for these inconsistencies, we believe this point warrants further discussion. For example, what specific residual mismatches remain? What mechanisms might plausibly give rise to the observed inconsistencies in behavior?

We agree to clarify the main residual inconsistencies (with reference to Figure S3D and more) so that future work can focus on these aspects. Figure S3D was referenced in the quoted statement, which invites readers to see the behavioral inconsistencies (p. 17).

> On capturing within-participant behavioral variability, although our model could reproduce some of the variability exhibited in human structure learning reasonably (Figure S3D), residual mismatches remain. For example, the DEE model exhibited greater trial-to-trial variability in cluster number reports than our participants when presented with identical stimulus sequences, suggesting that additional perceptual or cognitive mechanisms may be needed to account for the consistency observed in sequential structure learning. One possibility is that the brain infers structural information by integrating multiple recent observations held in another memory buffer (Radulescu et al. 2019), rather than updating beliefs based solely on the most recent observation. Such multi-observation integration could stabilize the structure learning process and reduce inferential variability. Future studies could query more reports per sequence from participants and vary the sequence length more systematically (e.g., Findling et al. 2019; Drugowitsch et al. 2016) to differentiate and model these noise components more precisely.

Reviewer #4 (Remarks to the Author):

I co-reviewed this manuscript with one of the reviewers who provided the listed reports. This is part of the Nature Communications initiative to facilitate training in peer review and to provide appropriate recognition for Early Career Researchers who co-review manuscripts.

> We thank you for providing helpful feedback.

Reviewers have no further comments.

Reviewer #2 (Remarks to the Author):

Fine, accept!

Reviewer #3 (Remarks to the Author):

The authors have fully addressed our concerns.
